# Genome-wide identification of Brassicaceae *B-BOX* genes and molecular characterization of their transcriptional responses to various nutrient stresses in allotetraploid rapeseed

**DOI:** 10.1186/s12870-021-03043-0

**Published:** 2021-06-24

**Authors:** Li-wei Zheng, Sheng-jie Ma, Ting Zhou, Cai-peng Yue, Ying-peng Hua, Jin-yong Huang

**Affiliations:** School of Agricultural Sciences, Zhengzhou University, Zhengzhou, 450001 China

**Keywords:** Allotetraploid rapeseed, BBX, Brassicaceae, Gene family, Molecular characteristics, Nutrient stress, Transcriptional responses

## Abstract

**Background:**

*B-box* (*BBX*) genes play important roles in plant growth regulation and responses to abiotic stresses. The plant growth and yield production of allotetraploid rapeseed is usually hindered by diverse nutrient stresses. However, no systematic analysis of Brassicaceae *BBXs* and the roles of *BBXs* in the regulation of nutrient stress responses have not been identified and characterized previously.

**Results:**

In this study, a total of 536 *BBXs* were identified from nine brassicaceae species, including 32 *AtBBXs*, 66 *BnaBBXs*, 41 *BoBBXs*, 43 *BrBBXs*, 26 *CrBBXs*, 81 *CsBBXs*, 52 *BnBBXs*, 93 *BjBBXs*, and 102 *BcBBXs*. Syntenic analysis showed that great differences in the gene number of Brassicaceae *BBXs* might be caused by genome duplication. The *BBXs* were respectively divided into five subclasses according to their phylogenetic relationships and conserved domains, indicating their diversified functions. Promoter *cis*-element analysis showed that *BBXs* probably participated in diverse stress responses. Protein-protein interactions between BnaBBXs indicated their functions in flower induction. The expression profiles of *BnaBBXs* were investigated in rapeseed plants under boron deficiency, boron toxicity, nitrate limitation, phosphate shortage, potassium starvation, ammonium excess, cadmium toxicity, and salt stress conditions using RNA-seq data. The results showed that different *BnaBBXs* showed differential transcriptional responses to nutrient stresses, and some of them were simultaneously responsive to diverse nutrient stresses.

**Conclusions:**

Taken together, the findings investigated in this study provided rich resources for studying Brassicaceae *BBX* gene family and enriched potential clues in the genetic improvement of crop stress resistance.

**Supplementary Information:**

The online version contains supplementary material available at 10.1186/s12870-021-03043-0.

## Background

B-box proteins (BBXs) are one of most important zinc finger transcription factors (TFs) owing to their essential roles in plant growth and development [1]. BBXs contain one to two conserved B-box domains [B-box 1 (B1) and B-box 2 (B2)] at the N-terminus, and some of them also share additional CONSTANS, CO-like, and TIMING of CAB1 (CCT) domain at the C-terminus [2]. BBX proteins are classified into five classes according to the number of B-box and CCT. In the classes I and II, BBXs consist of two B-boxes and a CCT, and B2 shows differences in the amino acid sequence between the two types of BBXs. Class III BBXs have one B1 and CCT domains, and class IV have both B1 and B2. However, in the class V BBXs, only B1 is identified [2].

BBXs have been reported to participate in photomorphogenesis, flower induction, shade avoidance, phytohormone signals, and stress responses [3, 4]. The hypocotyls of Arabidopsis *bbx4* mutant seedlings are longer than those of wild type under red light [5, 6], and *bbx2*, *bbx20*, and *bbx21* show long hypocotyls under both red and blue lights [5, 7]. However, the hypocotyl growth is inhibited by red, far red, and blue lights in *bbx24*, *bbx25* and *bbx32* [8–10]. *BBXs* are involved in regulating flowering by affecting photoperiodism. In Arabidopsis, AtBBX1 can activate *FLOWERING LOCUS T* (*FT*) by binding to its promoter under a long day time [11, 12]. AtBBX4 is likely to interact with AtBBX32, and they commonly control the *FT* expression [13]. Some *BBXs* are involved in gibberellic acid (GA), cytokinin (6-BA), and sucrose-mediated flowering induction in apples [14]. Shade avoidance usually leads to competitive plant growth, including hypocotyl and stem elongation, leaf angle increase, branch number decrease, and early blooming [15, 16]. *AtBBX1*, *AtBBX2*, *AtBBX21*, and *AtBBX22* participate in plant cell elongation under shade [17, 18]. *BBXs* play important roles in auxin (indoleacetic acid), GA, abscisic acid (ABA), and brassinosteroid (BR) signal transduction [19]. AtBBX21 and ABA insensitive 5 (ABI5) form a protein complex to participate in light-mediated ABA signals [20]. AtBBX18 is reported to increase the GA activity, and then promote hypocotyl growth [21]. BR signal TF BRASSINAZOLE-RESISTANT 1 (BZR1)-mediated hypocotyl elongation is repressed by AtBBX20 [22]. The crosstalk between OsBBX proteins (OsBBX8, OsBBX27, and OsBBX30) and the IAA signal is found in rice [23]. BBX proteins are also involved in the responses of plants to both biotic and abiotic stress stimuli in plants. *AtBBX18* can be induced by heat and shares a closed relationship with heat resistance. Overexpressing *AtBBX24* increases tolerance of Arabidopsis to salt [24]. A recent study suggested that *BBXs* are likely to participate in drought, cold, salt, and heavy metal stresses in rice, and several *BBXs* are involved in responses to hormonal signals [25]. A rice *BBX* gene, *OsCOL9*, enhances the resistance of rice to blast by mediating salicylic acid (SA) and ethylene (ETH) signals [26].

N is an essential macronutrient for seed yield and protein synthesis [27]. Nitrate (NO_3_^−^) and ammonium (NH_4_^+^) are two inorganic forms of N nutrients that can be absorbed by plants. However, most plant species cannot tolerate NH_4_^+^ excess, showing toxicity symptoms [28]. In agriculture, P is an essential nutrient for plant growth and development, and it has been widely used as fertilizers. The interaction between P and N can be predicted from the responses of N-responsive genes to P availability [29]. K is involved in chlorophyll biosynthesis and plant photosynthesis, and it can enhance the resistance of crops to drought, disease and cold [30]. Though micronutrients are the trace elements that are required for high plant, micronutrient deficiency can cause undesirable growth. For example, B plays important roles in cell wall synthesis, pollen tube elongation, carbohydrate transport, and N metabolism, and B deficiency often results in decreased crop production [31].

In Brassicaceae, several nutrient-related gene families have been identified, such as *amino acid* (*AA*) *permease* (*AAP*) and *aquaporin* (*AQP*) [32, 33]. However, the information about *BBX* genes is limited and their roles in nutrient regulation are not identified. In this study, a comprehensive study of *BBX* genes was performed in nine Brassicaceae species, including *Arabidopsis thaliana* (*A. thaliana*), *Brassica napus* (*B. napus*), *Brassica oleracea* (*B. oleracea*)*, Brassica rapa* (*B. rapa*), *Capsella rubella* (*C. rubella*), *Camelina sativa* (*C. sativa*), *Brassica nigra* (*B. nigra*), *Brassica juncea* (*B. juncea*) and *Brassica carinata* (*B. carinata*). The phylogenetic relationships, synteny, genomic structures, conserved domains, promoter sequences and protein protein interaction networks about *BBX* genes were analyzed. In addition, their responses to N limitation, P starvation, K shortage, and B deficiency, and B excess, NH_4_^+^ (A) toxicity, Cd treatment, and salt stress were investigated in allotetraploid rapeseed.

## Results

### Genome-wide identification of *BBX* genes and their phylogenetic analysis in Brassicaceae

**Fig. 1:**
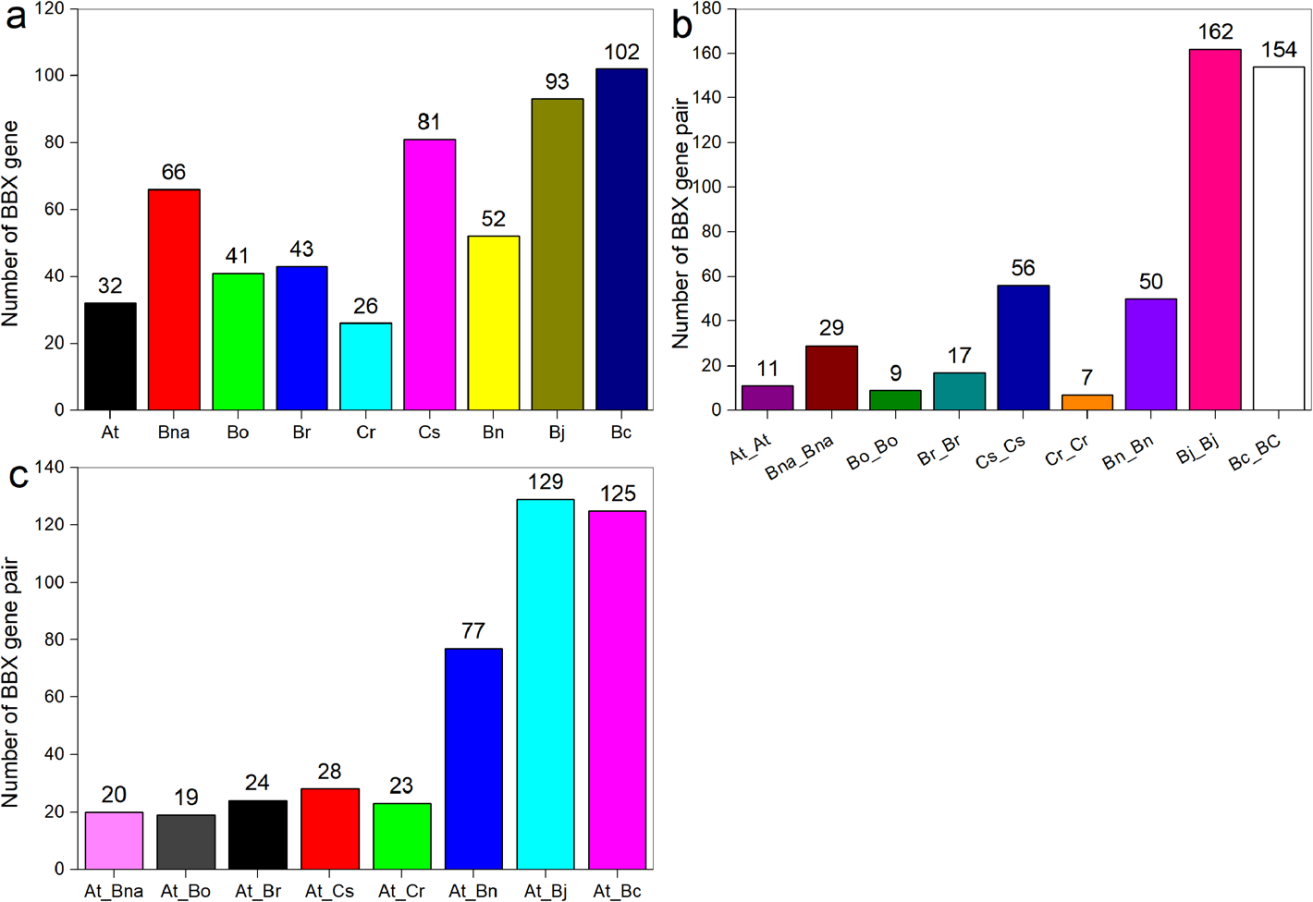
The gene number analysis of Brassicaceae *BBX*. **a** The number of *BBX* genes in *Arabidopsis* (At), *Brassica napus* (Bna), *Brassica oleracea* (Bo), *Brassica rapa* (Br), *Capsella rubella* (Cr), and *Camelina sativa* (Cs). **b** The number of *BBX* family gene pair among each Brassicaceae specie. **c** The number of BBX gene pair between *Arabidopsis* and each Brassicaceae specie

In the present study, a total of 536 *BBX* genes were identified (Fig. 1a), including 32, 66, 41, 43, 26, 81, 52, 93 and 102 *BBX* genes in *A. thaliana*, *B. napus*, *B. oleracea*, *B. rapa, C. rubella, C. sativa, B. nigra*, *B. juncea*, and *B. carinata*, respectively (Fig. 1a). Considering better understanding and consistency, they were named according to their location on chromosomes (Figure S1). The *BBX* genes were distributed on most chromosomes of each genome. Further, 12 *BBX* genes were identified in corresponding scaffolds, and the genome of *C. rubella* was assembled only to the scaffold level. For example, *BnaBBX1–26* were present respectively on chromosomes A01–10 and C01–09, but *BnaBBX52*–*66* were located on random chromosomes due to the deficiency of *B. napus* (allotetraploid rapeseed) genome assembly (Figure S1–1); Each *B. oleracea* chromosome, except for chromosome10, contained about four *BoBBXs*, whereas only one gene (*BoBBX30*) was present on chromosome 08 (Figure S1–2); All *B. rapa* chromosomes included *BrBBX* genes, and seven *BrBBXs* were identified on chromosomes A01 and A07; however, only *BrBBX16* in A04 and *BrBBX43* in scaffold000467 were found (Figure S1–3); In *C. rubella* scaffolds 1–4 and 6–8, 26 *CrBBXs* were found (Figure S1–4); *CsBBX1–80* were noted on 19 of 20 chromosomes, and *CsBBX81* was present in scaffold00495 (Figure S1–5).

**Fig. 2:**
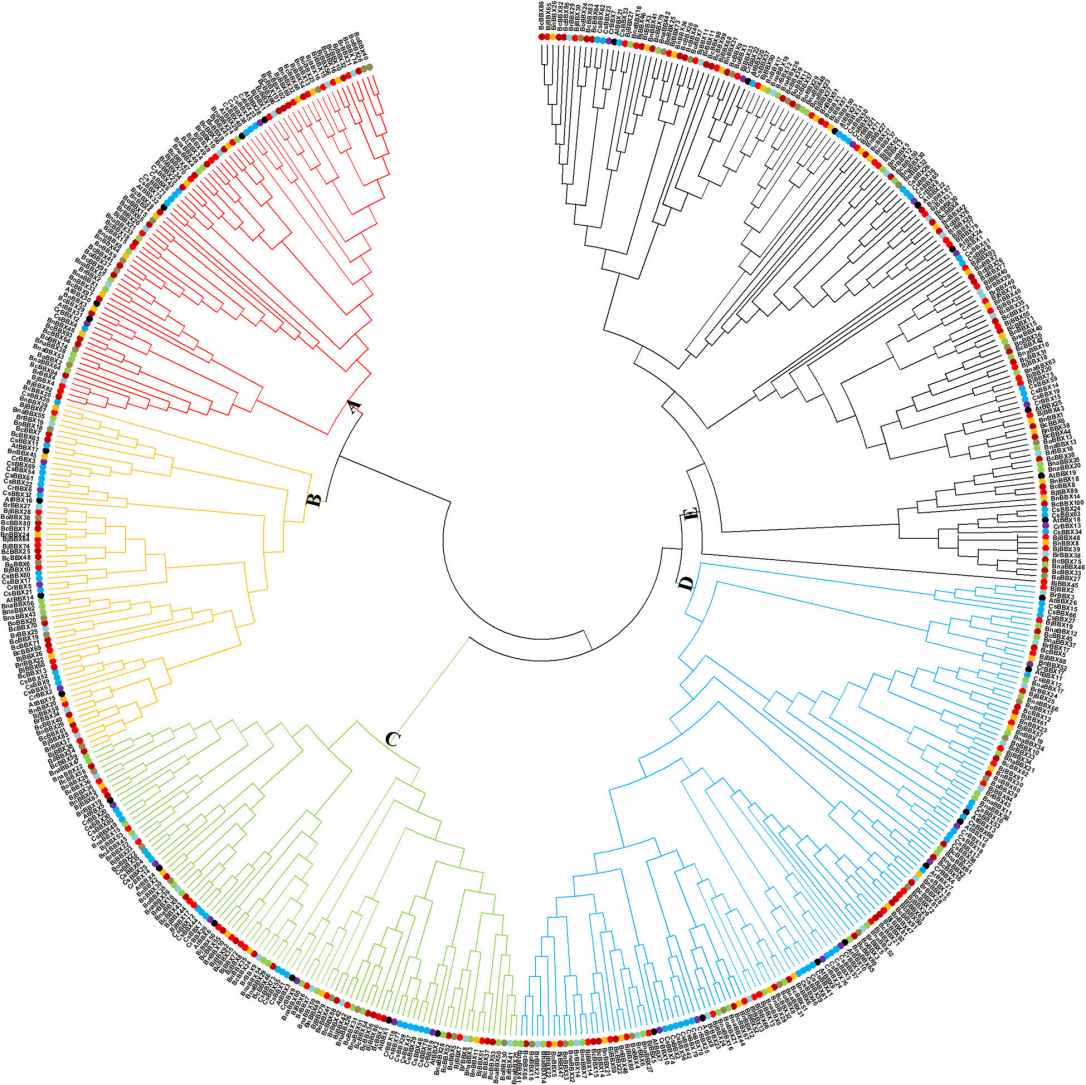
Phylogenetic analysis of *BBX* genes. Each Brassicaceae specie *BBX* genes were represented by colored circles

The unrooted maximum-likelihood phylogenetic tree classified Brassicaceae *BBXs* into five subclasses to identify putative orthologs (Fig. 2). Subgroup A comprised 8 *CsBBXs*, 2 *CrBBXs*, 10 *BnaBBXs*, 7 *BrBBXs*, 5 *AtBBXs*, 10 *BnBBXs*, 14 *BjBBXs*, and 30 *BcBBXs*. Subgroup B comprised 4 *AtBBXs* and 61 other species *BBXs*. A large number of *BBXs* were clustered together in subgroup C, and most of them were in gene pairs. The maximum number of *BBX* genes was observed in subfamily D. *CrBBX10*-*CsBBX74-CsBBX2-CsBBX56*, *CrBBX25*-*CsBBX42*-*CsBBX70*-*CsBBX79*, *CrBBX4-CsBBX16*, *CsBBX39-CsBBX41-CsBBX46-CsBBX81*, *CsBBX3-CsBBX76-CsBBX57-CrBBX11*, *CsBBX12*, *CsBBX18- CrBBX16* and *CsBBX68*, respectively, shared a closed evolutionary relationship with *AtBBX7, AtBBX8, AtBBX27, AtBBX9*, *AtBBX10*, *AtBBX11*, *AtBBX12*, and *AtBBX13* in subclass D. Subgroup E contained about 25% of *AtBBXs*, 22% of *BnaBBXs*, 24% of *BoBBXs*, 25% of *BrBBXs,* 26% of *CrBBXs*, 21% of *CsBBXs*, 36% of *BnBBXs*, 32% of *BjBBXs* and 30% of *BcBBXs*.

### Conserved domain and gene structure analysis of BBXs

BBXs were thought to be functionally or structurally significant in protein sequences. The output of “Batch Web CD-Search Tool” showed that all BBX proteins contained at least one B-box domain (Figure S2). The length of *BBX* genes in proteins and coding sequence (CDS) were identified to better analyze the BBX protein structure (Table S1). Proteins and CDSs were respectively from 97 to 876 amino acids (aa) and 294 to 2631 bp, respectively, and most of them were 400 aa long. About half of BnaBBXs contained approximately 400 aa, and these proteins included both B-box and CCT domains. Short BnaBBXs contained one or two B-box domains; Moreover, an SOG2 superfamily domain, which is involved in cell separation and cytokinesis, was identified in BnaBBX36 (Figure S2–1). There were two B-boxes (B1 and B2) and a CCT in most of BoBBXs, whereas the short proteins (such as BoBBX1 and BoBBX2) shared only one B-box. BoBBX5 and BoBBX11 separately included acetyl esterase (Aes) and SOG2 (Figure S2–2). Six BrBBXs (BrBBX1–4, BrBBX10, BrBBX15, BrBBX20, and BrBBX31) shared one B-box, while the other BrBBXs were made up of two domains (two B-boxes or a B-box and a CCT). Also, BrBBX28 shared two other domains [small ubiquitin-like modifier (UbI_SUMO_like) and CCCH-type Zn-finger (CTH1) superfamily] (Figure S2–3). At least two domains were found in all CrBBXs, except for CrBBX12 and CrBBX23; no additional domain was identified in CrBBXs (Figure S2–4). In terms of CsBBXs, the conserved domains of BBXs were also uncovered. Several domains (CsBBX7, CsBBX35 and CsBBX73) included double B-box and CCT; moreover, extra protein of the unknown function (DUF4621) domain was identified in CsBBX22 (Figure S2–5). B-box and CCT domains were identified in all BnBBX, besides, there were UbI_SUMO_like and CTH1 in BnBBX25 (Figure S2–6). Except for B-box and CCT, PLN02929 (PLN02929), Glyco_hydro_47 (Glycosyl hydrolase family 47), and Cupredoxin domains (involved in intermolecular electron transfer reactions) were also found in several BjBBX proteins (Figure S2–7). In terms of BcBBXs, it was worth noting that BCBBX6 contained a bzip_plant_RF2 domain (Figure S2–8). The protein sequences of conserved domains of BBXs were further analyzed in the Pfam database to determine their conserved domains (Figure S3). As predicted, conserved B-boxes and CCT were found in BBXs, but with exceptions. For example, BnaBBX1, BnaBBX4, BoBBX1, BrBBX1–2, CrBBX23, CsBBX43, BnBBX8, BjBBX1, and BcBBX4 contained only a B-box. However, no motifs were identified in many BBX proteins, including BrBBX2, BrBBX8, BoBBX2, BoBBX9, BrBBX3–4, CrBBX4, CrBBX12, CsBBX4, CsBBX10, BnBBX3, BjBBX2, BcBBX1, and so on, in the Pfam database (data not shown). This might be due to the delayed update of the Pfam database.

**Fig. 3:**
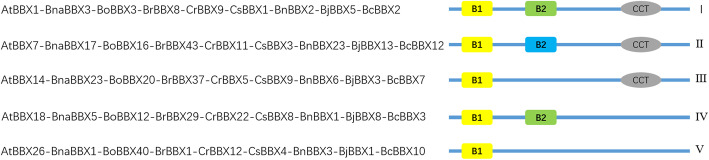
Diagrammatic shows protein structures of the representatives of each specie *BBX* gene family

**Fig. 4:**
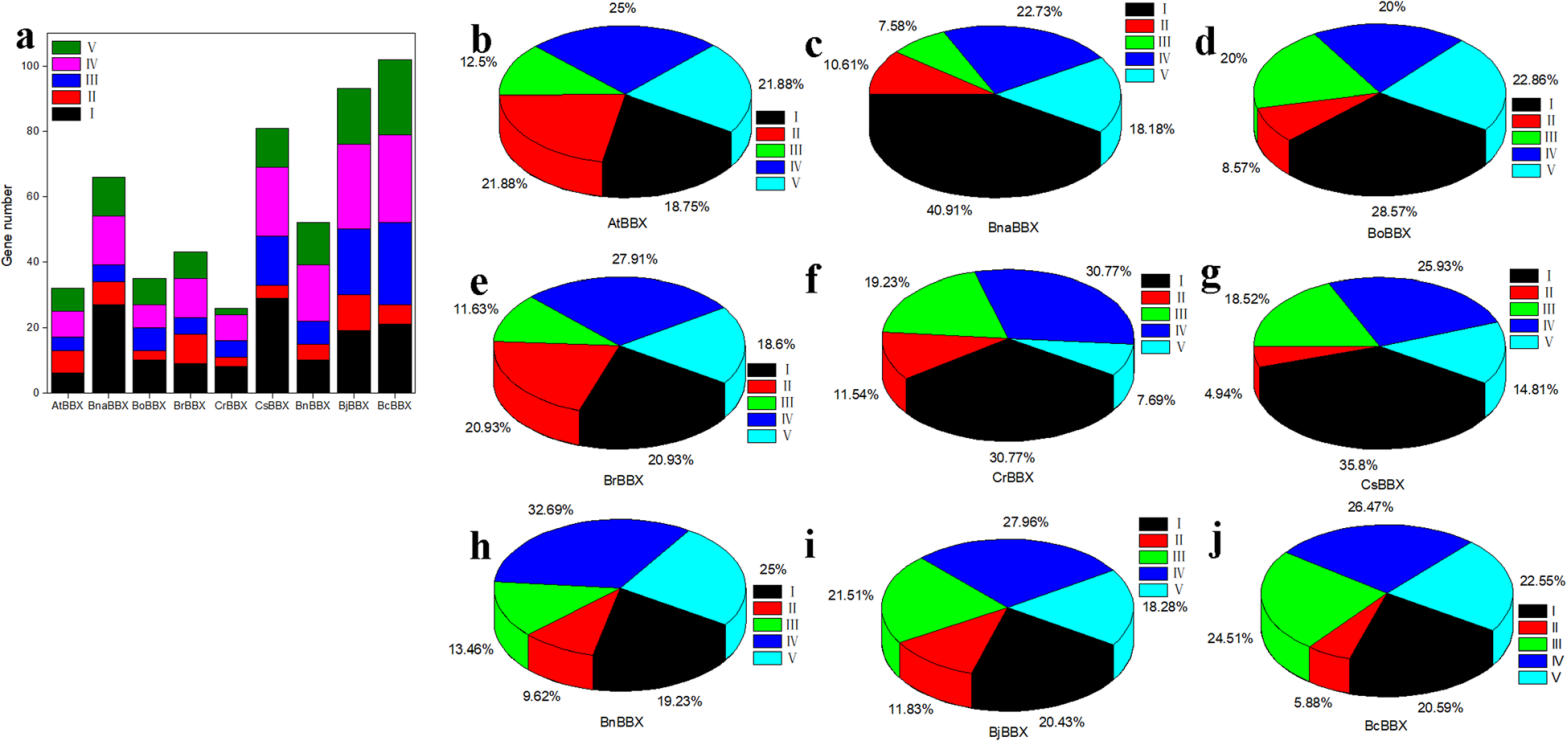
Comparison of structures of BBX protein. **a** The number of *BBX* genes in each structure. **b-g** Proportion of protein structures represented by I, II, III, IV, and V in each Brassicaceae specie *BBX* genes

According to a previous study [2], BBXs could be divided into five types. The protein types of Brassicaceae BBXs are listed in Table S1, and the representatives of each type are shown in Fig. 3. For example, the representatives of type I contained AtBBX1, BnaBBX3, BoBBX3, BrBBX8, CrBBX9, CsBBX1, BnBBX2, BjBBX5 and BcBBX2. Structure I-V comprised 6, 7, 4, 8, and 7 AtBBXs; 5–27 BnaBBXs were classified into five structures; The number of BoBBXs in structures I-V were from 3 to 10;, 5 to 12 BrBBXs were present in each type; The number of each type of CrBBXs was from 2 to 10. In class I-V, 29, 4, 15, 21, and 12 CsBBXs were identified, respectively (Fig. 4a). In *Arabidopsis*, most (25%) of *AtBBX* genes were in type IV, and a few ones belonged to type III (Fig. 4b). Most of *BBXs* among *B. napus*, *B. oleracea*, *C. rubella*, *C. sativa*, *B. nigra*, *B.juncea* and *B. carinata*, were in type I or IV, while a few *BBXs* were in type II or III (Fig. 4c-e and g-j). The number of BrBBXs in each type was in the order: IV = I > III > II > V (Fig. 4f).

Gene structures were involved in gene evolution. Therefore, a detailed *BBX* structure analysis was performed (Figure S4). Most of *BnaBBXs* shared more than one CDS, with the exceptions of *BnaBBX1*, *BnaBBX3–4*, *BnaBBX8*, *BnaBBX24–25*, *BnaBBX28*, *BnaBBX33*, *BnaBBX38*, *BnaBBX49–50*, *BnaBBX53*, *BnaBBX57*, *BnaBBX59–60*, and *BnaBBX64* (Figure S4–1). The length of *BnaBBXs* ranged from 100 to 6500 bp. More than 94% of *BoBBX* genes contained at least two CDSs. The length of a majority of *BoBBXs* was from about 800 to 1800 bp, and long *BoBBX2* and *BoBBX18* were mainly due to their non-CDS (Figure S4–2). About half of *BrBBXs* were constructed by one or two CDSs, and *BrBBX28* was the longest gene (Figure S4–3). Among *CrBBXs*, long untranslated regions (UTRs) of *CrBBX8* and *CrBBX18–20* were interesting (Figure S4–4). About 90% of CSBBX genes contained at least one intron (Figure S4–5). Most of *BnBBX* and *BjBBX* genes were less than 2.0 kb in length, while *BnBBX14*, *BnBBX25*, *BjBBX7*, *BjBBX31*, *BjBBX45*, *BjBBX67*, *BjBBX71*, and *BjBBX73* contained long introns (Figures S4–6 and S4–7). All *BcBBXs*, except for *BcBBX18*, *BcBBX20*, *BcBBX28*, *BcBBX29, BcBBX34*, *BcBBX37, BcBBX52, BcBBX53*, *BcBBX56*, and so on, shared one to six introns (Figure S4–8).

### Synteny and promoter analysis of *BBX* genes

Gene expansion occurs during the evolution of species. This study investigated their segment duplication in duplicated blocks within each genome to identify the expansion patterns of *BBX* genes in Brassicaceae species. A total of 495 pairs of *BBXs* were identified within nine genomes, including 11 pairs of *AtBBXs*, 29 pairs of *BnaBBXs*, 9 pairs of *BoBBXs*, 17 pairs of *BrBBXs*, 56 pairs of *CsBBXs*, 7 pairs of *CrBBXs*, 50 pairs of *BnBBXs*, 162 pairs of *BjBBXs*, and 154 pairs of *BcBBXs* (Fig. 1b). The duplicated *BBXs* in gene pairs were evenly distributed on most of chromosomes in each species (Figure S5). For example, almost all of *B. napus* chromosomes contained *BnaBBX* genes, and several chromosomes (chrA01, chrA03, chrA04, chrA07, chrC01, chrA02_random, chrA07_random, and chrC01_random) shared only one gene pair (Figure S5–2). Seven of nine *B. oleracea* chromosomes contained *BoBBX* genes in pairs, and 2, 1, 3, 2, 3, 1, and 1 *BoBBXs* were, respectively, found on chromosomes C01, C02, C03, C05, C07, C08, and C09 (Figure S5–3). The values of nonsynonymous (Ka) and synonymous substitution (Ks) were calculated to understand selection pressure among the aforementioned *BBX* gene pairs during the evolutionary process (Table S2). The Ka values of *BBX* gene pairs ranged from 0 (*CsBBX50*-*CsBBX31*, *CsBBX63-CsBBX34*, and *BjBBX17*-*BjBBX44*) to 0.45 (CsBBX37-CsBBX33) with an average of 0.14, and their Ks values ranged from 0.0086 (*CsBBX43*-*CsBBX6*) to 5.02 (*CsBBX37*-*CsBBX33*) with an average of 0.65. Then, Ka/Ks values of these gene pairs were calculated. They were all found to be less than 1.0, indicating that these genes might experience negative selection.

Besides, the syntenic maps of *AtBBXs* and each of Brassicaceae *BBXs* were also created. Further, 20, 19, 24, 28, 23, 77, 129, and 125 gene pairs were identified in *A. thaliana*-*B. napus*, *A. thaliana*-*B. oleracea*, *A. thaliana*-*B. rapa*, *A. thaliana*-*C. sativa*, *A. thaliana*-*C. rubella*, *A. thaliana*-*B. nigra*, *A. thaliana*-*B.juncea*, and *A. thaliana*-*B. carinata* (Fig. 1c)*.* More than 60% *AtBBXs* were identified to be orthologous of *BnaBBXs*, which were distributed over 11 *B. napus* chromosomes (Figure S6–1). About half of *AtBBXs* and 14 *BoBBXs* were found in 19 gene pairs, and *A. thaliana* chromosome C09 and *B. oleracea* chromosome 4, respectively, contained most of *AtBBXs* and *BnaBBXs* (Figure S6–2). Twenty-four *A. thaliana*-*B. rapa BBX* gene pairs were identified from 13 chromosomes of two species. The duplicated *AtBBXs* were located on chromosomes 1–5, and the duplicated *BrBBXs* were located on chromosomes A01, A04, and A05–10 (Figure S6–3). Also, 23 *AtBBXs* and 15 *CrBBXs* were localized to duplicated genomic regions on five chromosomes and six scaffolds, respectively (Figure S6–4). Eighteen *CrBBX* genes on seven chromosomes and 28 *AtBBX* genes on five chromosomes were identified as syntenic genes (Figure S6–5). All genes in *A. thaliana*-*B. nigra*, *A. thaliana*-*B.juncea*, and *A. thaliana*-*B. carinata BBX* pairs were distributed over all their chromosomes (Figures S6–7, S6–8, and S6–9). The ratios of Ka/Ks were calculated to understand the divergence among orthologous gene pairs of *B. napus* and *Arabidopsis*, *B. oleracea* and *Arabidopsis*, *B. rapa* and *Arabidopsis*, *C. rubella* and *Arabidopsis*, *C. sativa* and *Arabidopsis*, *B. nigra* and *A. thaliana*, *B.juncea* and *A. thaliana*, and *B. carinata* and *A. thaliana* (Table S3)*.* The values of Ka/Ks were less than 0.7, and the average Ka/Ks ratio of gene pairs between two species was 0.21, which was smaller than that between single species. This indicated that negative selection dominated the evolution of Brassicaceae *BBX* genes.

The promoters of *BnaBBXs*, *BoBBXs*, *BrBBXs*, *CrBBXs*, *CsBBXs, BnBBXs*, *BjBBXs*, and *BcBBXs* were analyzed. Stress-related elements [i.e., methyl jasmonate (MeJA)-responsiveness element, defense and stress responsiveness element, ABA responsiveness element, low-temperature responsiveness element, SA responsiveness element and drought-inducibility element] were found (Figure S7). For example, in *BnaBBXs* and *BoBBXs*, most of *cis*-elements were related to abscisic acid and salicylic acid responsiveness; and other stress-related elements were also identified (Figures S7–1 and 7–2). Further, 2 to 17 ABA responsiveness elements were observed in *BrBBXs*, except for *BrBBX3*, *BrBBX11*, *BrBBX27*, and *BrBBX28* (Figure S7–3).

### Protein–protein interaction analysis of *BnaBBXs* and their responses to B

**Fig. 5:**
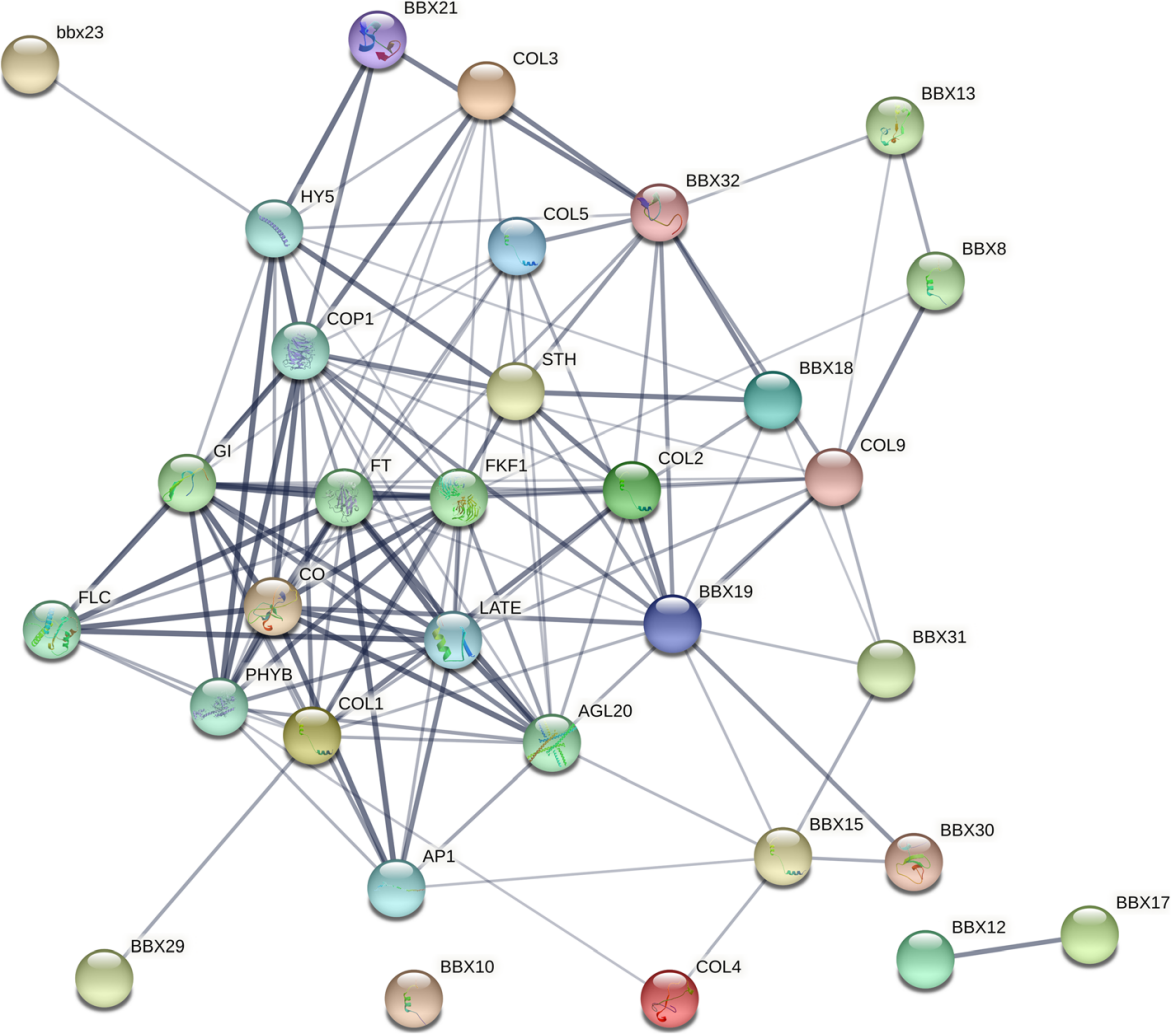
Protein-protein interaction networks of BnaBBX proteins

Protein–protein interactions have been proved to be effective in uncovering biochemical functions of targets [34]. Many BnaBBX proteins potentially interacted with each other, with exceptions of BnaBBX52, BnaBBX65, BnaBBX12, and BnaBBX55 (Fig. 5). Besides, other partners of BnaBBXs that were also identified included gigantea protein (GI), flavin-binding kelch repeat f box 1 (FKF1), FLOWERING LOCUS T (FT), MADS-box protein AGAMOUS-LIKE 20 (AGL20), FLOWERING LOCUS C (FLC), phytochrome B (PHYB), transducin/WD40 repeat-like superfamily protein constitutive photomorphogenesis protein 1 (COP1), basic leucine zipper (bZIP) transcription factor [Hypocotyl5 (HY5)], MADS-box transcription factor APETALA1 (AP1), and C2H2-like zinc finger protein LATE FLOWERING (LATE) (Fig. 5). Most of these patterns were involved in flowering. For example, FT is a mobile flower-promoting signal that promotes the transition of a plant from vegetative growth to flowering; In long days, CO interacting with FT promotes flowering; AGL20 acts downstream of FT to regulate growth phase transitions. LATE acts as a transcriptional repressor of flowering. AP1 is indispensable for the normal development of sepals and petals in flowers. The remaining patterns (FKF1, PHYB, COP1, and HY5) take part in light-dependent circadian cycles. In addition, AtCO (BnaBBX26 and BnaBBX51) can strongly interact with 11 proteins (GI, HY5, COP1, FT, FKF1, LATE, BBX19, AGAL20, AP1, PHYB, and FLC).

**Fig. 6:**
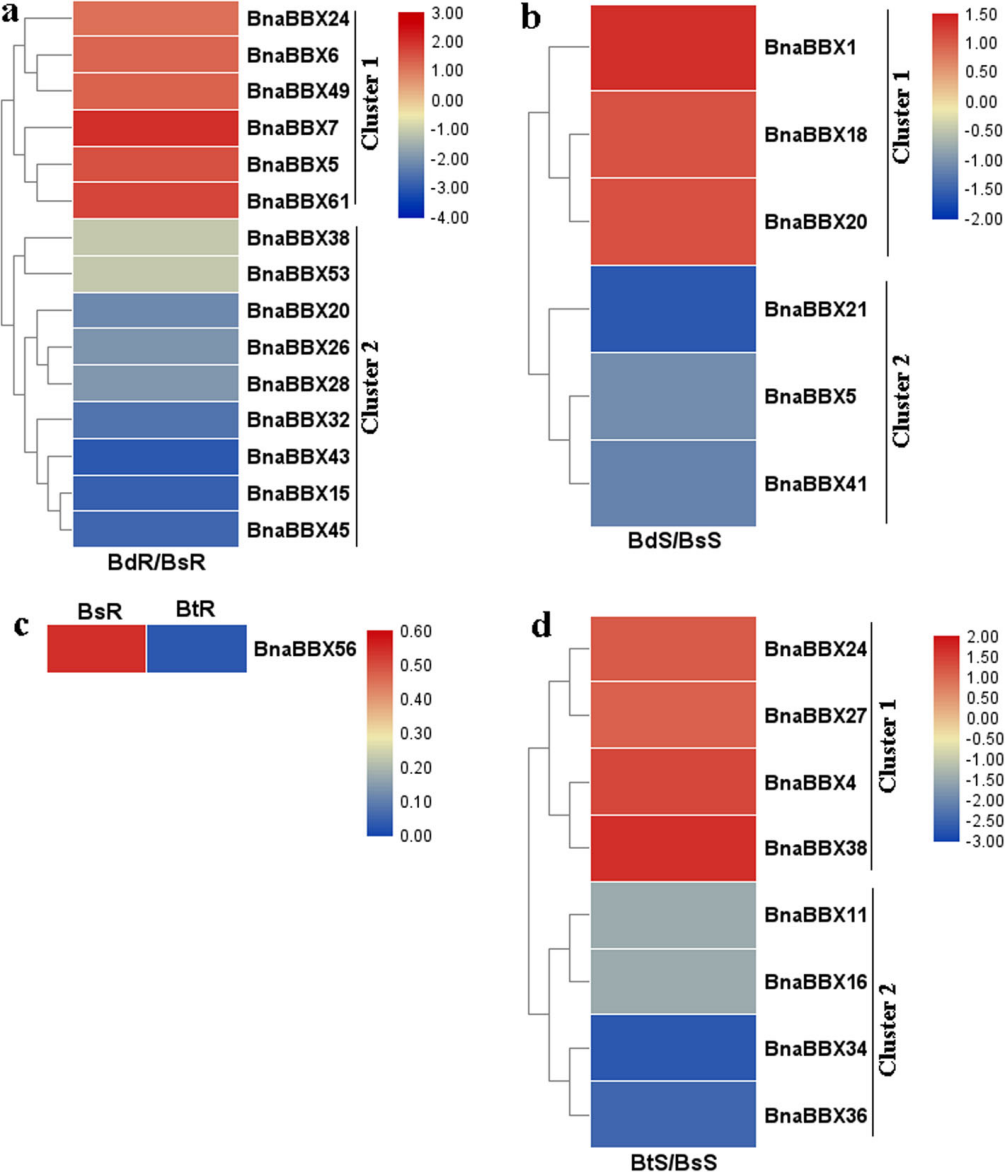
The expression patterns of *BnaBBX* genes under low and high B levels. **a** Expression analysis of *BnaBBXs* in root under low and normal B supply levels. **b** Expression analysis of *BnaBBXs* in shoot under low and normal B supply levels. **c** Expression analysis of *BnaBBXs* in root under excess and normal B supply levels. **d** Expression analysis of *BnaBBXs* in shoot under excess and normal B supply levels. Chart legend indicates the values of log2(fold-change)

B deficiency retards leaf and root growth but increases the number and length of root hairs in root tips [35]; and B toxicity is involved in leaf necrosis and primary root growth [35]. However, whether *BnaBBXs* take part in B-mediated plant growth is unknown. Differentially expressed *BnaBBXs* were identified under deficient- and excess-B conditions to elucidate the effects of B on *BnaBBX* gene expression (Fig. 6). In the roots, six *BnaBBXs*, especially *BnaBBX7*, were induced after low B treatment in cluster 1, while nine *BnaBBXs*, especially *BnaBBX15*, *BnaBBX43*, and *BnaBBX45*, were obviously repressed in cluster 2 (Fig. 6a). Six *BnaBBXs* were differentially expressed in shoots after B deficiency treatment; half of them were upregulated in cluster 1, while others were downregulated in cluster 2 (Fig. 6b). B toxicity also influenced *BnaBBX* expression (Fig. 6c and d). Only *BnaBBX56* was repressed by excess B in the roots (Fig. 6c). After B toxicity treatment, *BnaBBX24*, *BnaBBX27*, *BnaBBX4* and *BnaBBX38* were induced in the shoots, while another four *BnaBBXs* were inhibited (Fig. 6d).

### Effect of N, P, and K on the expression of *BnaBBX* genes

**Fig. 7:**
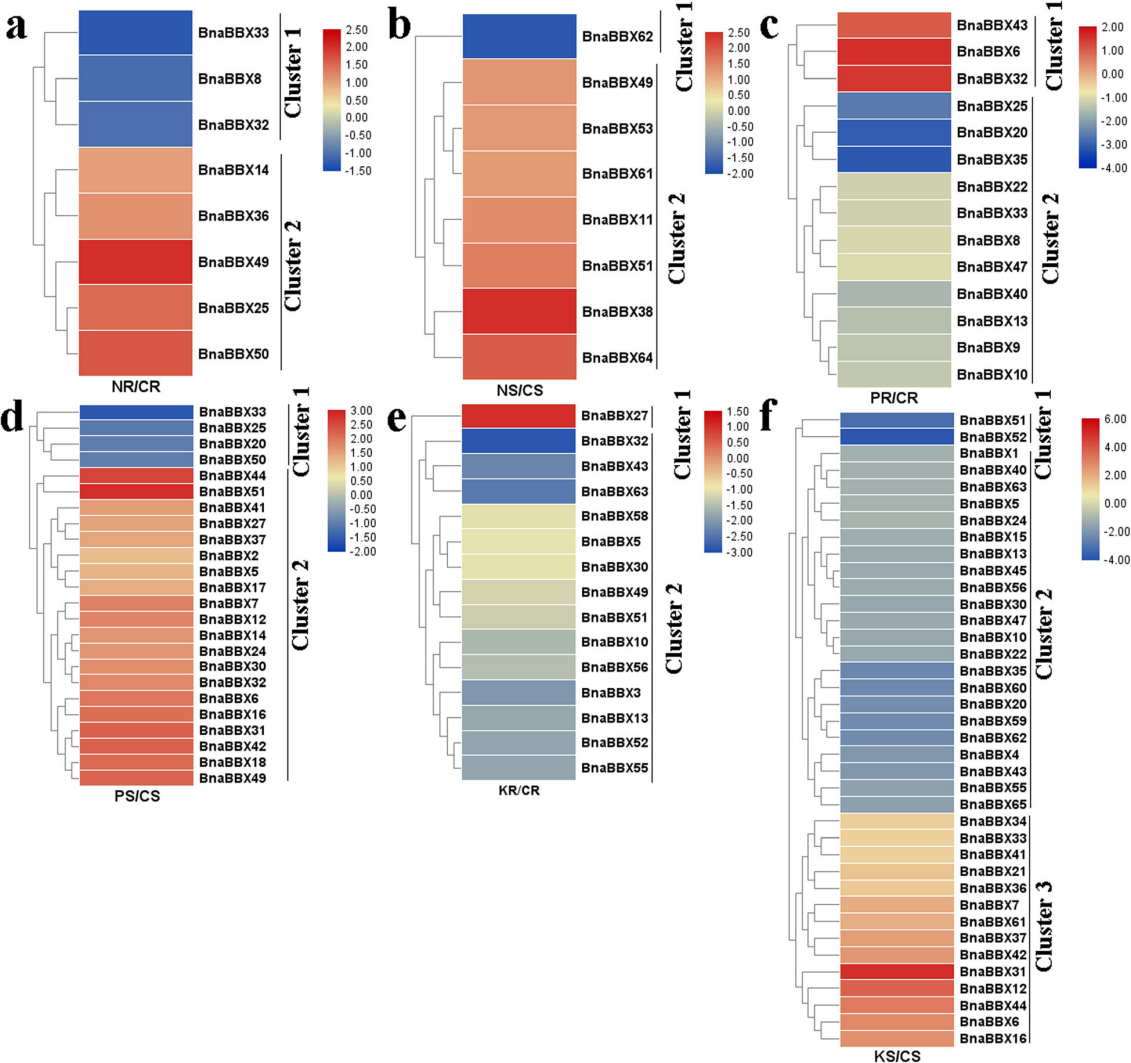
The expression patterns of *BnaBBX* genes under different N, P and K levels. **a** Expression analysis of *BnaBBXs* in root under low and normal N supply levels. **b** Expression analysis of *BnaBBXs* in shoot under low and normal N supply levels. **c** Expression analysis of *BnaBBXs* in root under low and normal P supply levels. **d** Expression analysis of *BnaBBXs* in shoot under low and normal K supply levels. **e** Expression analysis of *BnaBBXs* in root under low and normal K supply levels. **f** Expression analysis of *BnaBBXs* in shoot under low and normal K supply levels. Chart legend indicates the values of log2(fold-change)

N nutrients are highly demanded in rapeseed, but N use efficiency is low [36]. N limitation affects leaves, roots, nitrate (NO_3_^−^), total N and anthocyanin concentrations, and glutamine synthetase activity in *B. napus* [37]. However, the molecular mechanisms underlying the use of nitrogen by plants have not been fully understood, and the information about *BnaBBX* involvement in N metabolism is limited. The transcript levels of *BnaBBX* genes after low-N treatment were investigated to better understand the potential roles of *BnaBBXs* in assimilating N. Under N stress, the expression of eight *BnaBBXs* was significantly altered in the roots; *BnaBBX33*, *BnaBBX8*, and *BnaBBX32* were downregulated, but the remaining ones were upregulated (Fig. 7a). In the shoots, eight *BnaBBXs* responded to N stress; only *BnaBBX62* was controlled in the N-treated group, and seven *BnaBBXs* (*BnaBBX49*, *BnaBBX53, BnaBBX61*, *BnaBBX11*, *BnaBBX51*, *BnaBBX38*, and *BnaBBX64*) were obviously induced (Fig. 7b).

P is widely present in most fertilizers and is required for normal plant growth [38]. The expression of three *BnaBBXs* (*BnaBBX43*, *BnaBBX6*, and *BnaBBX32*) clearly increased in the roots after P stress treatment, but the expression of eleven, especially *BnaBBX20*, *BnaBBX25*, and *BnaBBX35,* was suppressed (Fig. 7c). In the shoots, more *BnaBBXs* were affected by P stress. The expression of *BnaBBX33*, *BnaBBX25*, *BnaBBX20*, and *BnaBBX50* was inhibited by P in cluster 1, while 20 *BnaBBXs*, especially *BnaBBX44* and *BnaBBX51*, showed higher expression levels under P deficiency in cluster 2 (Fig. 7d).

K is an important macronutrient for plant vegetative and reproductive growth [39]. In low K treated group, *BnaBBX27* was induced in the roots, but the expression of 14 *BnaBBXs* (*BnaBBX32*, *BnaBBX43, BnaBBX63, BnaBBX58, BnaBBX5, BnaBBX30*, *BnaBBX49*, *BnaBBX51*, *BnaBBX10, BnaBBX56, BnaBBX3*, *BnaBBX13*, *BnaBBX52* and *BnaBBX55*) were significantly decreased (Fig. 7e). In the shoots, K deficiency resulted in an obvious decrease in the expression of *BnaBBXs*; especially the expression of *BnaBBX51–52*, expressions in cluster 1 and 2 and that of 14 *BnaBBX* genes in cluster 3, particularly *BnaBBX31*, were increased (Fig. 7f).

### Effects of a, cd, and salt on the expression of *BnaBBX* genes

**Fig. 8:**
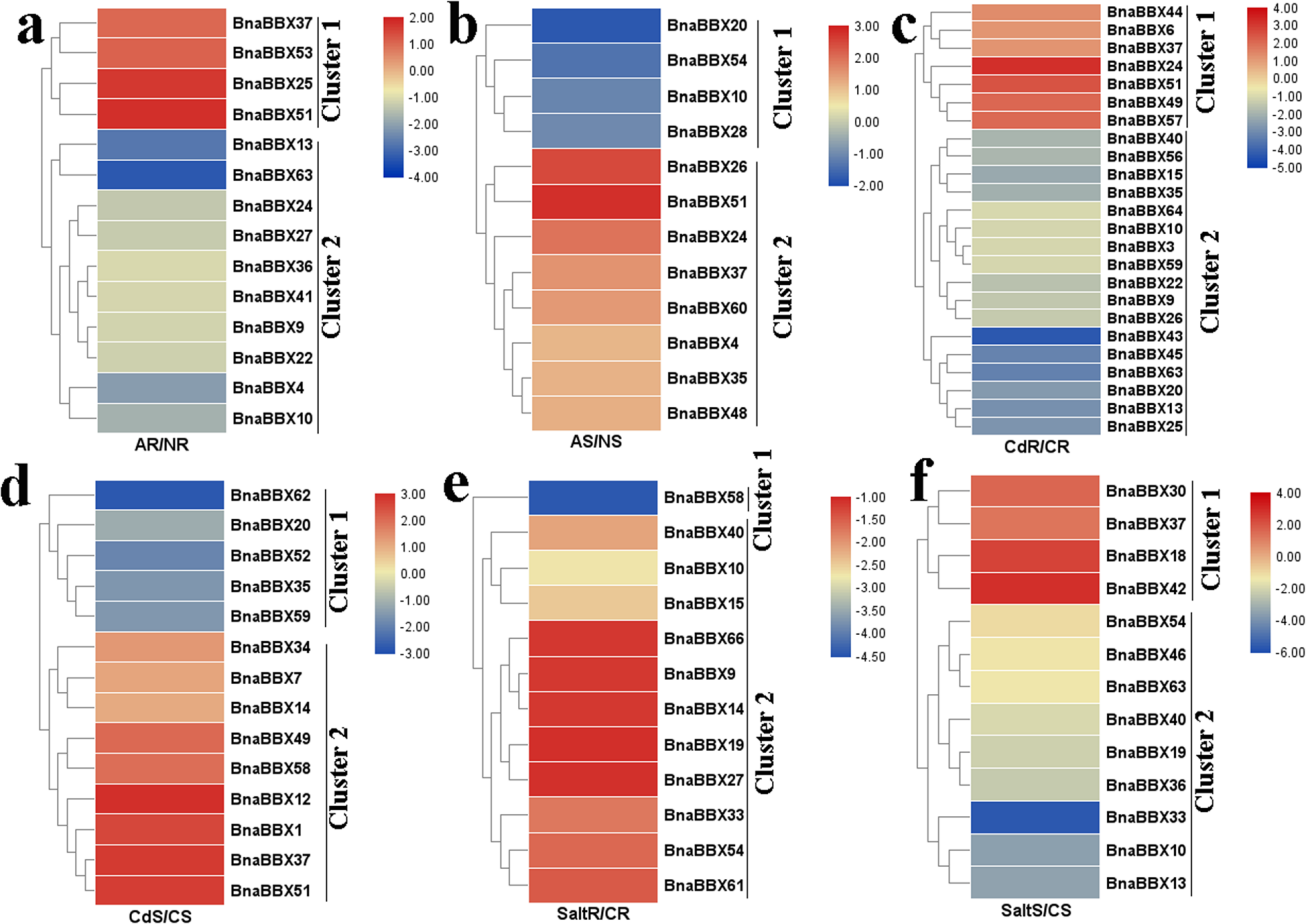
The expression patterns of *BnaBBX* genes under A, Cd and salt toxicity. **a** Expression analysis of *BnaBBXs* in root under A toxicity and A free. **b** Expression analysis of *BnaBBXs* in shoot under A toxicity and A free. **c** Expression analysis of *BnaBBXs* in root under Cd toxicity and Cd free. **d** Expression analysis of *BnaBBXs* in SHoot under Cd toxicity and Cd free. **e** Expression analysis of *BnaBBXs* in root under salt toxicity and salt free. **f** Expression analysis of *BnaBBXs* in shoot under salt toxicity and salt free. Chart legend indicates the values of log2(fold-change)

At proper supplies, A is a major inorganic N source for plant growth, whereas it causes toxicity at excess external supplies [40]. *BnaBBX* expression profiles were evaluated to understand their potential involvement in responses to A toxicity. A total of 14 *BnaBBXs* were differentially expressed in the roots relative to the condition of excess A (Fig. 8a). *BnaBBX37*, *BnaBBX53, BnaBBX25* and *BnaBBX51* were upregulated by A, while the expression levels of other genes were decreased. In the shoots, among differentially expressed 12 *BnaBBXs*, the expression of four *BnaBBXs* (*BnaBBX20*, *BnaBBX54, BnaBBX10* and *BnaBBX28*) were decreased after A toxicity, but eight *BnaBBXs* showed higher expression levels under A toxicity compared with the control condition (Fig. 8b).

Cd is a harmful heavy metal for plant growth and development. Rapeseed has been widely used to restore cadmium-contaminated soils. After Cd treatment, necrotic spots on the young leaves of seedlings were observed, and root hair growth was also affected [41]. However, the molecular mechanism underlying Cd toxicity resistance has not been fully revealed. In the roots, seven and 17 *BnaBBXs* exhibited higher or lower expression, respectively, in the Cd-treated group compared with the control group (Fig. 8c). In the shoots, nine *BnaBBXs* were significantly elevated under Cd toxicity in cluster 1, while Cd repressed the expression of *BnaBBX62*, *BnaBBX20, BnaBBX52, BnaBBX35,* and *BnaBBX59* in cluster 2 (Fig. 8d).

Salt stress constrains plant growth and clearly reduces rapeseed yield [42, 43]. The salt altered the expression of 25 *BnaBBXs* in the roots and shoots (Fig. 8e and f). Under salt stress, *BnaBBX58* showed a low expression level in the roots in cluster 1, but 11 *BnaBBXs* shared higher expression levels in cluster 2 (Fig. 8e). After salt treatment, the expression of *BnaBBX30*, *BnaBBX37, BnaBBX18*, and *BnaBBX42* was distinctly upregulated in the shoots, while the expression of nine *BnaBBXs* was obviously downregulated (Fig. 8f).

### Diverse responses of *BnaBBXs* to various nutrient stress signals

**Fig. 9:**
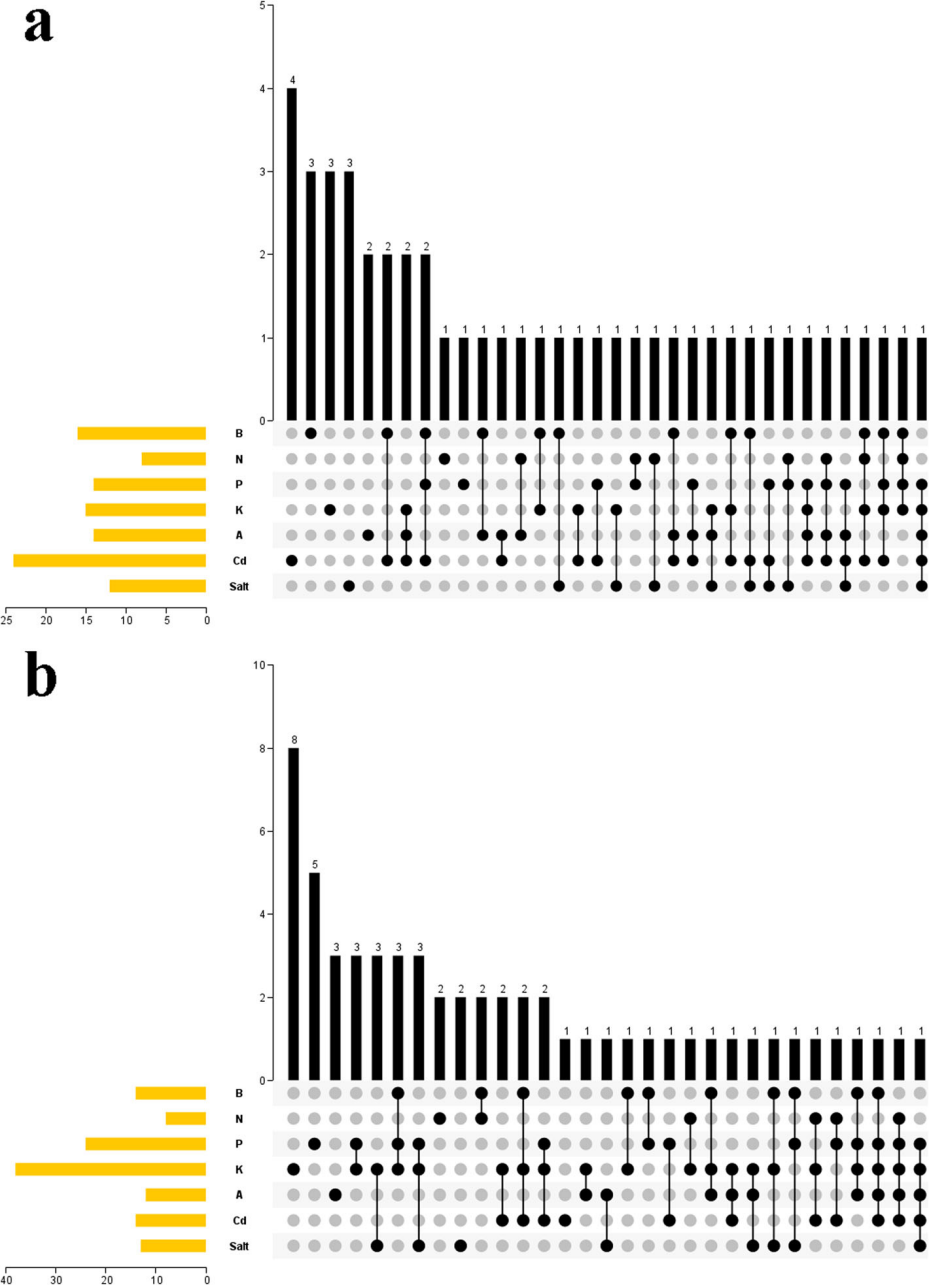
Venn diagram showing the transcriptional responses of *BnaBBXs* under diverse nutrient stresses. **a** The number of differentially expressed *BnaBBX* genes in root of *Brassica napus* under diverse nutrient stresses. **b** The number of differentially expressed *BnaBBX* genes in shoot of *Brassica napus* under diverse nutrient stresses. Chart legend indicates the values of log2(fold-change)

A Venn diagram was constructed to investigate whether *BnaBBXs* were responsive to diverse nutrient stresses simultaneously (Fig. 9a and b). Many *BnaBBX* genes were affected by more than one stress signal at the same time, and several ones only responded to only single nutrient stress (Fig. 9a and b, and Tables S4, and S5). For example, one (*BnaBBX10*) and three (*BnaBBX20*, *BnaBBX51* and *BnaBBX37*) *BnaBBXs* were separately regulated by five nutrient stresses in the roots and shoots at once. Six and one *BnaBBXs* were, respectively, responsive to four signals in roots and shoots. A total of 28 *BnaBBXs* simultaneously responded to three nutrient stresses in two tissues. About 26 *BnaBBXs* were simultaneously regulated by two stresses. However, some *BnaBBXs* (*BnaBBX44*, *BnaBBX38*, *BnaBBX55*, *BnaBBX54*, *BnaBBX41*, *BnaBBX53, BnaBBX64*, and so on) were only controlled by a single stress in the roots or shoots.

## Discussion

*BBX* family members have been reported to play critical roles in plant growth and stress responses [1, 5, 6]. However, the information about Brassicaceae *BBXs* is limited so far. In this study, 536 *BBX* genes were identified, including 32 *AtBBXs*, 66 *BnaBBXs*, 41 *BoBBXs*, 43 *BrBBXs*, 26 *CrBBXs*, 81 *CsBBXs*, 52 *BnBBXs*, 93 *BjBBXs*, and 102 *BcBBXs.* Their conserved domain, gene structure, gene phylogeny, promoter, and synteny analyses were completed. The BnaBBX protein-protein interaction network was constructed. Moreover, differential expression profiles of *BnaBBX* genes under N limitation, P shortage, K deficiency, B starvation, A excess, Cd toxicity, and salt stress were delineated. These results might provide an integrated insight into BBX family genes.

### Comparison of *BBX* genes among nine Brassicaceae species

The identification of 32 *BBX* genes has been systematically reported in *Arabidopsis* [2]. In this study, 66 *BnaBBXs*, 41 *BoBBXs*, 43 *BrBBXs*, 26 *CrBBXs*, 81 *CsBBXs*, 52 *BnBBXs*, 93 *BjBBXs*, and 102 *BcBBXs* were respectively identified (Fig. 1a). The number of *BnaBBXs*, *BoBBXs*, *BrBBXs*, *CrBBXs*, *CsBBXs*, *BnBBXs*, *BjBBXs*, and *BcBBXs* was nearly 2-, 1.2-, 1.3-, 0.8-, 2.5-, 1.6-, 2.9-, and 3.1-fold of *AtBBXs*, rspectively. Generally, gene duplication results in gene family expansion [44]. Then, orthologous *BBX* genes were detected in the syntenic maps. The 11 pairs of *AtBBXs*, 29 pairs of *BnaBBXs*, 9 pairs of *BoBBXs*, 17 pairs of *BrBBXs*, 7 pairs of *CrBBXs,* 56 pairs of *CsBBXs*, 50 pairs of *BnBBXs*, 162 pairs of *BjBBXs* and 154 pairs of *BcBBXs* were respectively identified (Fig. 1b). The result indicated that segmental gene duplication was likely to increase the expression of *BnaBBXs*, *BrBBXs*, *CsBBXs*, *BnBBXs*, *BjBBXs*, and *BcBBXs*. In addition, genome-wide duplication events that occurred million years ago contributed to chromosome reduplication. Therefore, whole-genome duplications were predicted to mainly induce the expansion and evolution of *BBX* family in *B. oleracea* and *C. rubella*.

These *BBXs* were belonged to six different subfamilies, and the gene number of each subfamily varied, indicating their different evolutionary patterns (Fig. 2). The syntenies between duplicated blocks in *Arabidopsis-B. napus*, *Arabidopsis-B. oleracea*, *Arabidopsis-B. rapa*, *Arabidopsis-C. rubella*, *Arabidopsis-C. sativa*, *Arabidopsis-B. nigra*, *Arabidopsis-B.juncea*, and *Arabidopsis-B. carinata* were also determined to predict the unknown functions of the *BnaBBXs*, *BoBBXs*, *BrBBXs*, *CrBBXs*, *CsBBXs*, *BnBBXs*, *BjBBXs* and *BcBBXs* according to *AtBBXs*. The 20, 19, 24, 28, 23, 77, 129 and 125 gene pairs were respectively found in *Arabidopsis-B. napus*, *Arabidopsis-B. oleracea*, *Arabidopsis-B. rapa*, *Arabidopsis-C. rubella*, *Arabidopsis-C. sativa*, *Arabidopsis-B. nigra*, *Arabidopsis-B.juncea*, and *Arabidopsis-B. carinata*, respectively (Fig. 1c). These *BnaBBX*, *BoBBX*, *BrBBX*, *CrBBX*, *CsBBX*, *BnBBX*, *BjBBX* and *BcBBX* genes in pairs were considered to originate from common ancestors with *AtBBXs*, indicating their similar functions with corresponding *Arabidopsis* ones. For example, *AtBBX4*, *AtBBX20*, *AtBBX24* and *AtBBX32* were involved in red-, far-red- or blue light-mediated photomorphogenesis [8, 10, 13, 18, 19]; Therefore, their homologous genes (*BrBBX40*-*BnBBX2*-*BjBBX17*-*BcBBX30*-*BcBBX43*, *CsBBX19*-*CrBBX15-BnBBX17-BjBBX68-BcBBX39-BcBBX9-BcBBX98*, *BnaBBX39-BnBBX7-BjBBX18-BcBBX11*, and *BoBBX16*-*BrBBX16*-*CsBBX35*-*CrBBX20*-*BnaBBX36*-*BoBBX24*-*BrBBX32*-*CsBBX33*-*CrBBX22- BnBBX4-BjBBX21-BcBBX18*) were likely to take part in plant photomorphogenesis.

Conserved domains were always associated with gene functions. Therefore, typical domains were investigated in *BBX* gene families. According to a previous study [2], *BBX* genes were divided into classes I-V (Fig. 3). BBXs in type IV shared the largest proportion among AtBBXs, BrBBXs, CrBBXs, BnBBXs, BjBBXs, and BcBBXs, while most of BnaBBX, BoBBX, CrBBXs, and CsBBXs were in type I (Fig. 4). Moreover, double B-boxes or CCT domains were found in CsBBX7, CsBBX35 and CsBBX73 (Figure S2–5). Different types of *BBX* genes showed different biological functions [2]. Therefore, the main roles of *BBX* genes may vary among different Brassicaceae species. Except for B-boxes and CCT domains, other domains were also found in several BBXs (Figure S2). For example, SOG2 superfamily was in BnaBBX36 and BoBBX11, Aes in BoBBX5, Ubl_SUMO_like and CTH1 superfamily in BrBBX28 and BnBBX25, and DUF4621 superfamily in CsBBX22. Also, some unknown domains were found in AtBBX proteins [5, 11, 45]. This result suggested that *BoBBX5*, *BoBBX11*, *CsBBX22*, *BrBBX28*, *BnaBBX36*, and *BnBBX25* shared other functions associated with these additional motifs.

### Putative functions of *BnaBBXs* in stress responses and flower induction

Large numbers of MeJA-responsiveness, defense and stress responsiveness, abscisic acid responsiveness, low-temperature responsiveness, SA responsiveness or drought-inducibility elements were found in five Brassicaceae *BBXs*, and ABA responsiveness elements were mainly present in all *BBXs* (Figure S7); ABA was involved in seed dormancy and germination, root growth, abiotic and biotic stress response, and inducing stomatal closure [1, 46, 47]. These findings indicated that *BBXs* were involved in stress tolerance, especially ABA-mediated biological processes.

The expression patterns of *BnaBBXs* were investigated to identify their functions in regulating various nutrient stresses. B, N, P, and K were nutrients required for plant growth; they had essential roles in diverse plant physiological pathways and oxidative stresses [33, 35, 36]. In the roots and shoots, 21 *BnaBBXs* were upregulated or downregulated by B limitation, and 9 genes were affected by B toxicity (Fig. 6). Eight *BnaBBXs* were altered in the upper and lower parts of allotetraploid rapeseed seedlings after N stress (Fig. 7a and b). Thirty-eight *BnaBBXs* were differentially expressed in response to P insufficiency (Fig. 7c and d). In the roots and shoots, many *BnaBBXs* were influenced by K stress (Fig. 7e and f). These results provided valuable information about *BnaBBXs* involvement in regulating nutrient assimilation; these genes were candidate regulators in the roots or shoots.

Various adverse environmental factors, including drought, salinity, heat, and cold, negatively affect plant growth and development [48, 49]. This study found that many *BnaBBX* genes were sensitive to A, Cd, and salt, and their expression was greatly altered by these stresses in the roots or shoots (Fig. 8). The transcriptional activity of some *OsBBX* genes was reported to be greatly affected by Cd [25]. The expression patterns of three *OsBBXs* were altered by salt stress [25]. *AtBBX24* promoted root growth under a high-salinity condition [24]. However, few studies focused on *BBX*-mediated A adaptation. The results of the present study indicated that, like *AtBBXs* and *OsBBXs*, *BnaBBXs* might be involved in response to Cd and salt stresses; also, they might possibly participate in A metabolism. Future studies should focus on the exact roles of the *BnaBBX* gene family in adaptation to these stresses.

In addition, this study investigated that many *BnaBBXs* were simultaneously responsive to diverse stresses (Fig. 9, Tables S4, and S5). For example, the expression of *BnaBBX10*, *BnaBBX37*, *BnaBBX51*, and *BnaBBX20* was affected by five nutrient stresses in the shoots or roots at the same time. Also, several *BnaBBX* genes were involved in four, three, or two stress signals in two parts of *B. napus*. According to the phylogenetic analysis, *BoBBX36* and *BcBBX42* were the homologous genes of *BnaBBX10*; *BnaBBX37*, *BnaBBX51*, and *BnaBBX20*, respectively, got together with *BrBBX17*, *BcBBX54*, and *BcBBX38* (Fig. 2). However, some genes were only under the control of a single nutrient stress. The afore-mentioned findings indicated that many *BnaBBXs* and their homologs played core roles in response to various nutrient stresses; and some were only particular in a specific stress. However, these need to be verified in the near future.

## Conclusions

This study was novel in performing the genome-wide analysis of genes belonging to the Brassicaceae *BBX* family. The information regarding their chromosomal location, conserved domain, gene structure, synteny, promoter sequence, and protein-protein interaction network was identified. *BnaBBX* expression profiles in response to B deficiency and toxicity, N limitation, P shortage, K starvation, A excess, Cd toxicity, and salt stress were delineated. A large number of *BnaBBXs* were involved in improving rapeseed resistance to nutrient stresses in the roots or shoots; some of them were identified as the core regulators in this process. The data generated in this study provided a comprehensive understanding of *BBX* gene family evolution and involvement of *BnaBBX* genes in stress responses and flower induction. Future studies should focus on validating the functions of *BBXs*.

## Methods

### Identification, conserved domain, chromosome location, phylogenetic relationship, and gene structure of *BBX* genes

Using known AtBBX protein sequences as queries, *B. napus*, *B. oleracea, B. rapa, C. rubella, C. sativa*, *B. nigra, B.juncea*, and *B. carinata* protein databases were searched using “Blast Several Sequences to a Big Database” in TBtools software [50], with an expected value (e-value) of e^− 5^. The conserved domains in BBXs were confirmed in Pfam (http://pfam.xfam.org/) and Batch Web CD-Search Tool (https://www.ncbi.nlm.nih.gov/Structure/bwrpsb/bwrpsb.cgi) databases and, respectively, visualized using “Visualize Pfam Domain Pattern” and “Visualize NCBI CDD Domain Pattern” tools [50]. The candidates lacking characteristic domains were eliminated from further analyses. With chromosome length and gene position files, *BBXs* genes were mapped onto chromosomes using “Gene Location Visualize (Advanced)” of TBtools [50].

For phylogenetic analysis, MEGA X was used to construct the phylogenetic tree by the maximum likelihood method. Protein sequences were aligned with default parameters using the ClustalW program. With generic feature format version 3 (gff3) files of BBXs, a Visualize Gene Structure (Basic) tool [50] was used to draw the gene structure diagram.

### Homology and synteny analysis, identification of *cis*-acting elements in promoters of *BBX* genes, BnaBBXs protein-protein interaction networks, and heatmap analyses

“One Step MCScanX” of TBtools [50] was used to analyze *BBX* duplication events with genome sequences and gff3 files. “Table Row Extract Or Filter”, “File Transformat For Microsyteny Viewer And Advanced Circos”, “Fasta stats”, and “File Merge For MCScanX” tools of TBtools software were used to visualize the synteny of *BBX* gene pairs according to a previous study [51]. Nonsynonymous (Ka) and synonymous (Ks) substitutions of each *BBX* gene pair were calculated using a Simple Ka/Ks Calculator (NG) [50]. The *cis*-elements in the 3000-bp genomic sequence upstream of the start codon were analyzed in the PlantCARE online tool (http://bioinformatics.psb.ugent.be/webtools/plantcare/html/), and “Simple Biosequence Viewer” of TBtools [50] was used for visualization. STRING 10 (http://string-db.org/) (option value > 0.800) was used to construct an interaction network, with interolog proteins from *Arabidopsis*, to analyze the relationship of BnaBBXs with other proteins. The heat maps were generated with the “HeatMap” tool of TBtools [50].

### Plant materials and treatments

Uniform *B. napus* (Zhongshuang 11) seedlings of 7-d old were transplanted into black plastic containers containing Hoagland nutrient solution (5.0 mM KNO_3_, 1.0 mM KH_2_PO_4_, 2.0 mM MgSO_4_·7H_2_O, 5.0 mM Ca (NO_3_)_2_·4H_2_O, 0.10 μM Na_2_MoO_4_·2H_2_O, 0.050 mM EDTA-Fe, 0.80 μM ZnSO_4_·7H_2_O, 9.0 μM MnCl_2_·4H_2_O, 0.30 μM CuSO_4_·5H_2_O, and 46 μM H_3_BO_3_). Before use for treatments, *B. napus* seedlings were cultivated for 10 days (d) in a chamber under a light intensity of 300–320 μmol m^− 2^ s^− 1^, a temperature of 25 °C daytime/22 °C night, a light period of 16-h photoperiod/8-h dark, and relative humidity of 70%.

#### B deficiency and toxicity treatments

Seventeen-day-old rapeseed seedlings were, respectively, cultivated in 0.25 μM and 1500 μM H_3_BO_3_ for 10 d in B deficiency- and excess-treated groups.

#### N, P, and K depletion treatments

*B. napus* seedlings, 17-d old, were cultivated in Hoagland nutrient solution, including 0.30 mM N, 5mΜ P, and 0.30 mM K for 3 d.

#### NH4^+^ toxicity treatment

Uniform Zhongshuang 11 seedlings, 7 d old, were first cultivated in Hoagland nutrient solution containing normal nitrate for 10 d. Subsequently, they were transferred to an N-free solution for 3 d. Finally, the plants were subjected to 9.0 mM NH_4_^+^ (excess NH_4_^+^) for 6 h.

#### Cd toxicity and salt treatments

The Cd- and salt-treated 17-day-old Zhongshuang 11 seedlings were, respectively, grown in a solution containing 10 μM CdCl_2_ for 12 h and 200 mM NaCl for 1 d. The seedlings in the control groups were cultivated in a normal solution for appropriate times based on the aforementioned treatments. Comparative transcriptomes were performed using roots and shoots from control and stress-treated plants in previous studies [27, 33, 35, 41, 52, 53]; the transcriptome data can be found in published studies. Differentially expressed genes are defined as those with *P* value < 0.05, false-discovery rate less than 0.05, and |log_2_(fold-change)| ≥ 1.

## Supplementary Information

**Additional file 1: Figure S1.** Chromosomal location of *BBX* genes on Brassicaceae chromosomes. **Figure S1–1** The location of *BBX* genes on *B. napus* chromosomes. **Figure S1–2** The location of *BBX* genes on *B. oleracea* chromosomes. **Figure S1–3** The location of *BBX* genes on *B. rapa* chromosomes and scaffold. **Figure S1–4** The location of *BBX* genes on *C. rubella* scaffolds. **Figure S1–5** The location of *BBX* genes on *C. sativa* chromosomes and scaffold. **Figure S1–6** Chromosomal location of *B. nigra BBX* genes*.* **Figure S1–7** Chromosomal location of *B.juncea BBX* genes*.* **Figure S1–8** Chromosomal location of *B. carinata BBX* genes*.*

**Additional file 2: Fig**ure **S2.** Identification and characterization of conserved domains in BBXs in NCBI database. **Figure S2–1** Identification and characterization of the conserved domains in BnaBBXs. **Figure S2–2** Identification and characterization of the conserved domains in BoBBXs. **Figure S2–3** Identification and characterization of the conserved domains in BrBBXs. **Figure S2–4** Identification and characterization of the conserved domains in CrBBXs. **Figure S2–5** Identification and characterization of the conserved domains in CsBBXs. **Figure S2–6** Identification and characterization of the conserved domains in BnBBXs. **Figure S2–7** Identification and characterization of the conserved domains in BjBBXs. **Figure S2–8** Identification and characterization of the conserved domains in BcBBXs.

**Additional file 3: Fig**ure **S3.** Identification and characterization of conserved domains in BBXs in pfam database. **Figure S3–1** Identification and characterization of the conserved domains in BnaBBXs. **Figure S3–2** Identification and characterization of the conserved domains in BoBBXs. **Figure S3–3** Identification and characterization of the conserved domains in BrBBXs. **Figure S3–4** Identification and characterization of the conserved domains in CrBBXs. **Figure S3–5** Identification and characterization of the conserved domains in CsBBXs. **Figure S3–6** Identification and characterization of the conserved domains in BnBBXs. **Figure S3–7** Identification and characterization of the conserved domains in BjBBXs. **Figure S3–8** Identification and characterization of the conserved domains in BcBBXs.

**Additional file 4: Fig**ure **S4.** Structure analysis of *BBX* genes. **Figure S4–1** Exon–intron structures of *BnaBBX* genes. **Figure S4–2** Exon–intron structures of *BoBBX* genes. **Figure S4–3** Exon–intron structures of *BrBBX* genes. **Figure S4–4** Exon–intron structures of *CrBBX* genes. **Figure S4–5** Exon–intron structures of *CsBBX* genes. **Figure S4–6** Exon–intron structures of *BnBBX* genes. **Figure S4–7** Exon–intron structures of *BjBBX* genes. **Figure S4–8** Exon–intron structures of *BcBBX* genes.

**Additional file 5: Fig**ure **S5.** Synteny of *BBX* genes in each Brassicaceae genome. **Figure S5–1** Synteny of *AtBBX* genes in *A. thaliana* genome. **Figure S5–2** Synteny of *BnaBBX* genes in *B. napus* genome. **Figure S5–3** Synteny of *BoBBX* genes in *B. oleracea* genome. **Figure S5–4** Synteny of *BrBBX* genes in *B. rapa* genome. **Figure S5–5** Synteny of *CrBBX* genes in *C. rubella* genome. **Figure S5–6** Synteny of *CsBBX* genes in *C. sativa* genome. **Figure S5–7** Synteny of *BnBBX* genes in *B. nigra* genome. **Figure S5–8** Synteny of *BjBBX* genes in *B.juncea* genome. S109, C668, C1065 and C306 respectively indicates Super_scaffold_109_3169392_4351964_52914_567609, Contig668_1_483987, Contig1065 and Contig306 in the B.juncea genome. **Figure S5–9** Synteny of *BcBBX* genes in *B. carinata* genome. J0021.1, J1585.1, J1599.1 and J1604.1 respectively indicates JAAMPC010000021.1, JAAMPC0100001585.1, JAAMPC0100001599.1 and JAAMPC0100001604.1 in the *B. carinata* genome.

**Additional file 6: Fig**ure **S6.** Synteny of *BBX* genes between *A. thaliana* and each other Brassicaceae. **Figure S6–1** Synteny of *BBX* genes between *A. thaliana* and *B. napus*. **Figure S6–2** Synteny of *BBX* genes between *A. thaliana* and *B. oleracea*. **Figure S6–3** Synteny of *BBX* genes between *A. thaliana* and *B. rapa*. **Figure S6–4** Synteny of *BBX* genes between *A. thaliana* and *C. rubella.* **Figure S6–5** Synteny of *BBX* genes between *A. thaliana* and *C. sativa.* **Figure S6–6** Synteny of *BBX* genes between *A. thaliana* and *B. nigra.* **Figure S6–7** Synteny of *BBX* genes between *A. thaliana* and *B.juncea.* S109, C668, C1065 and C306 respectively indicates Super_scaffold_109_3169392_4351964_52914_567609, Contig668_1_483987, Contig1065 and Contig306 in the *B.juncea* genome. **Figure S6–8** Synteny of *BBX* genes between *A. thaliana* and *B. carinata*. J0021.1, J1585.1, J1599.1 and J1604.1 respectively indicates JAAMPC010000021.1, J AAMPC010001585.1, J AAMPC010001599.1 and J AAMPC010001604.1 in *B. carinata* genome.

**Additional file 7: Fig**ure **S7.** Promoter analysis of *BBX* genes. **Figure S7–1** *Cis*-element identified in promoters of *BnaBBXs.* **Figure S7–2** *Cis*-element identified in promoters of *BoBBXs*. **Figure S7–3** *Cis*-element identified in promoters of *BrBBXs*. **Figure S7–4** *Cis*-element identified in promoters of *CrBBXs.* **Figure S7–5** *Cis*-element identified in promoters of *CsBBXs*. **Figure S7–7** *Cis*-element identified in promoters of *BjBBXs*. **Figure S7–8** *Cis*-element identified in promoters of *BcBBXs*.

**Additional file 8: Table S1.** Information about identified *BBX* genes. **Table S2.** Synteny analysis of Brassicaceae *BBX* genes. **Table S3.** Synteny analysis of *Arabidopsis* and each other Brassicaceae *BBX* genes. **Table S4.** Common DEGs in root among different classes. **Table S5.** Common DEGs in root among different classes.

## Data Availability

All the data and materials that are required to reproduce these findings can be shared by contacting the corresponding author, Dr. Ying-peng Hua (yingpenghua@zzu.edu.cn).

## Abbreviations

BBX: B-box
B: Boron
Cd: Cadmium
Pi: Phosphate
K: Potassium
*A. thaliana*: *Arabidopsis thaliana*
*B. napus*: *Brassica napus*
*B. oleracea*: *Brassica oleracea*
*B. rapa*: *Brassica rapa*
*C. rubella*: *Capsella rubella*
*C. sativa*: *Camelina sativa*
N: Nitrogen
A: Ammonium (NH_4_^+^)
NO_3_^−^: Nitrate
DEGs: Differentially expressed genes
