# Supplementary figures and images for: Genome-wide identification of Brassicaceae B-BOX genes and molecular characterization of their transcriptional responses to various nutrient stresses in allotetraploid rapeseed
Source: BMC Plant Biol. 2021 Jun 24;21:288. doi: 10.1186/s12870-021-03043-0 (PMC8223294; doi:10.1186/s12870-021-03043-0)

Fig. S1-1


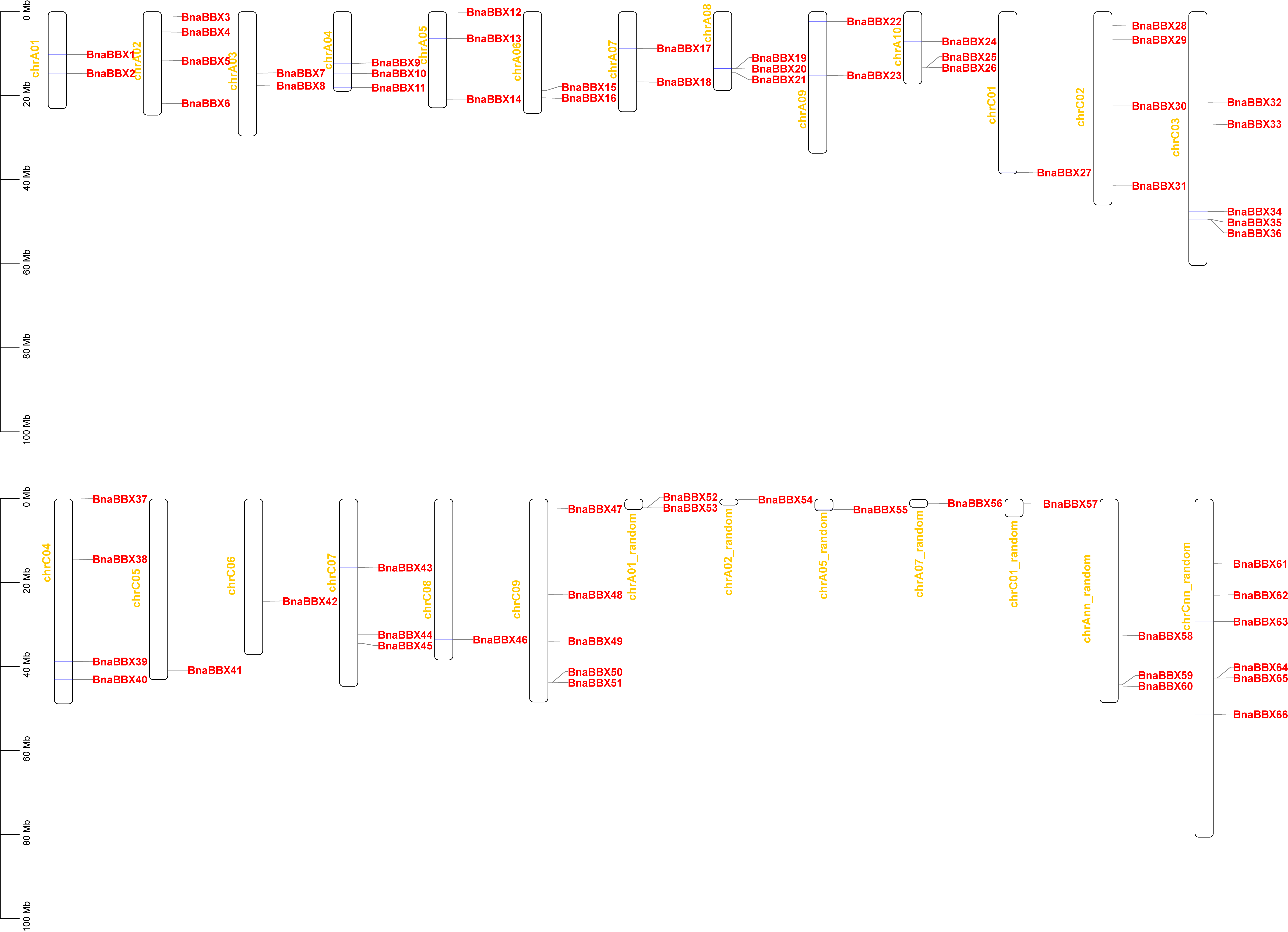


Fig. S1-2


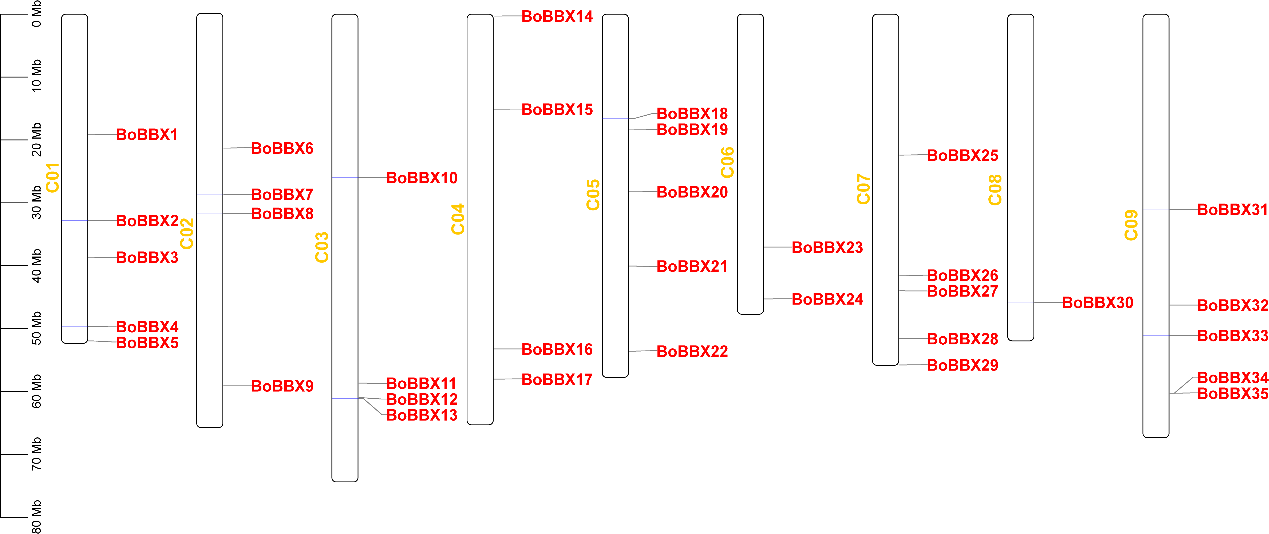


Fig. S1-3


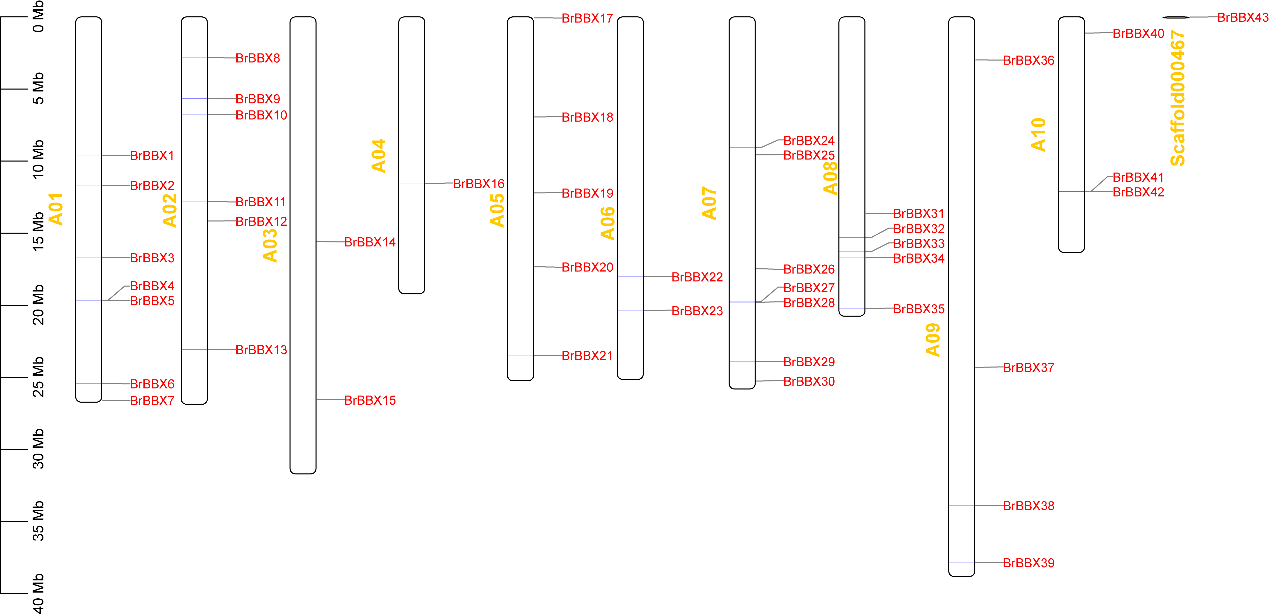


Fig. S1-4


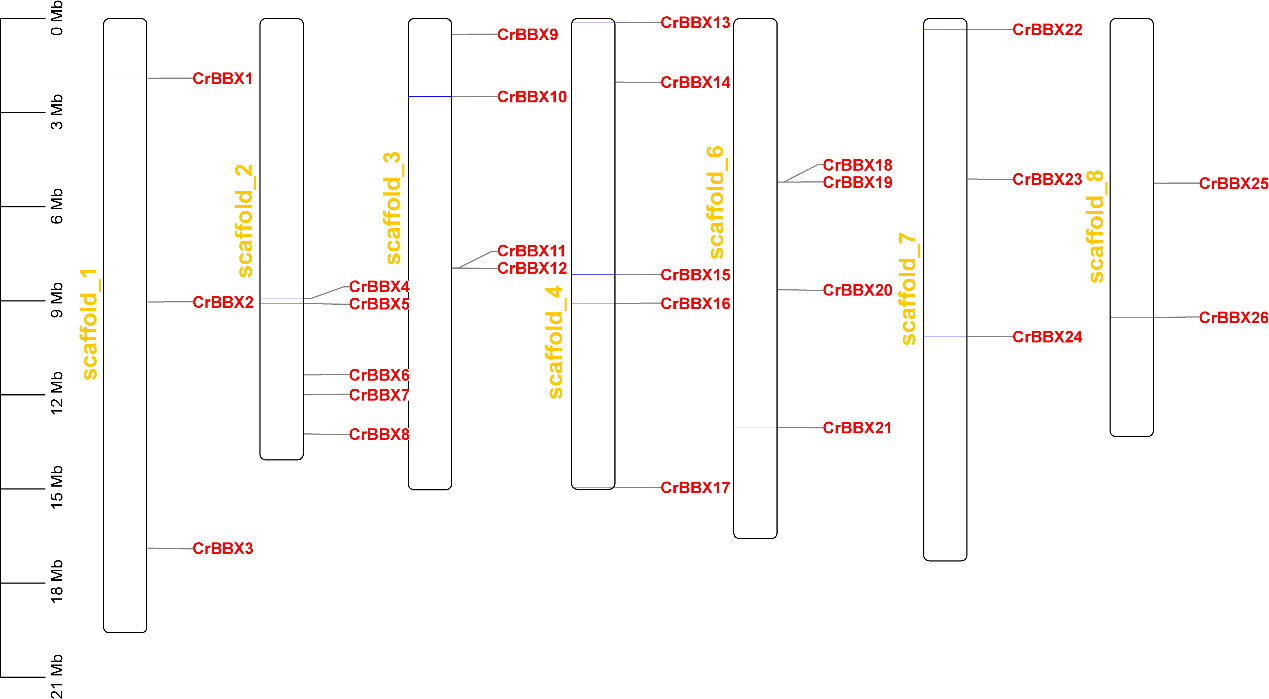


Fig. S1-5


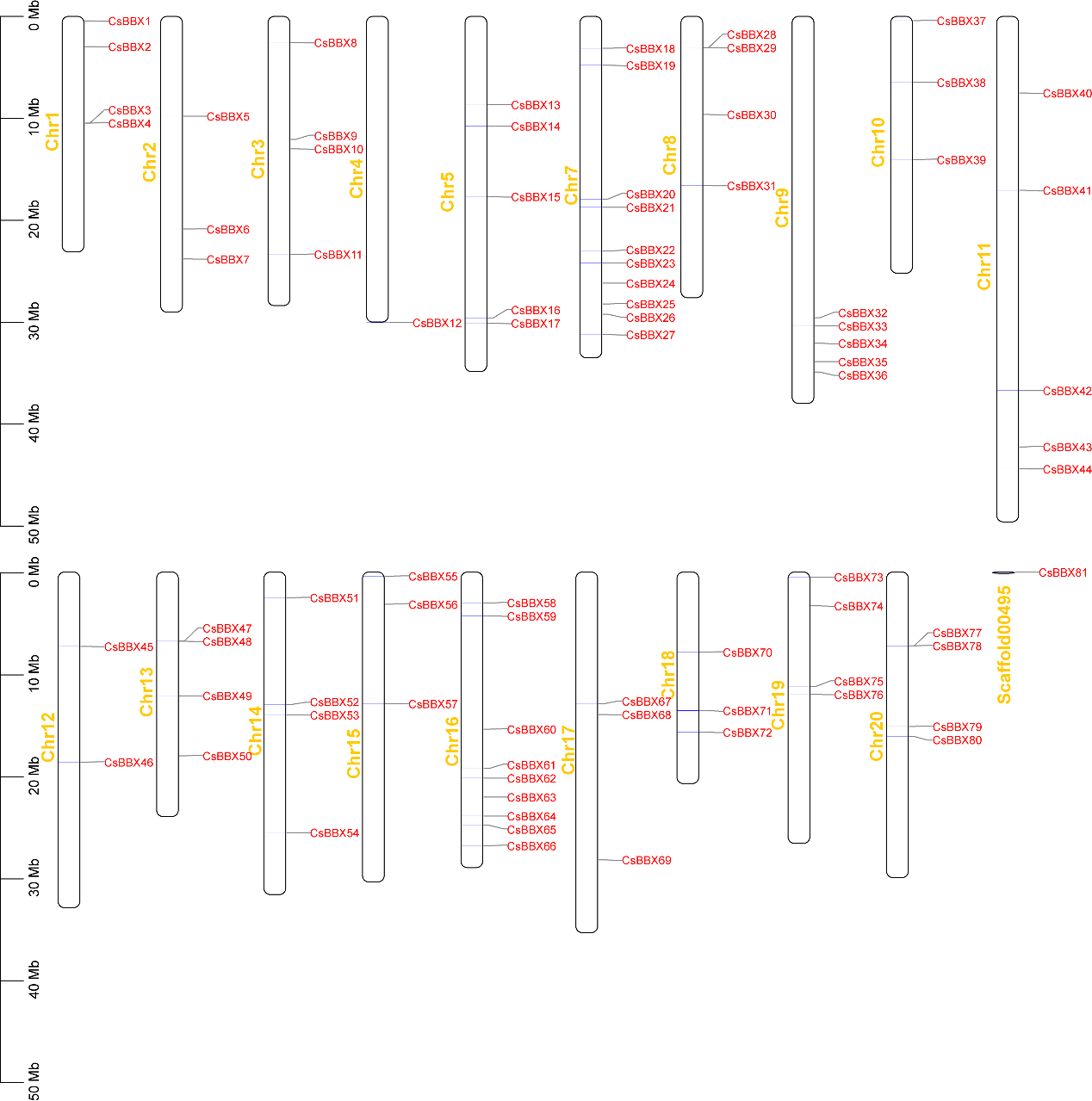


Fig. S1-6


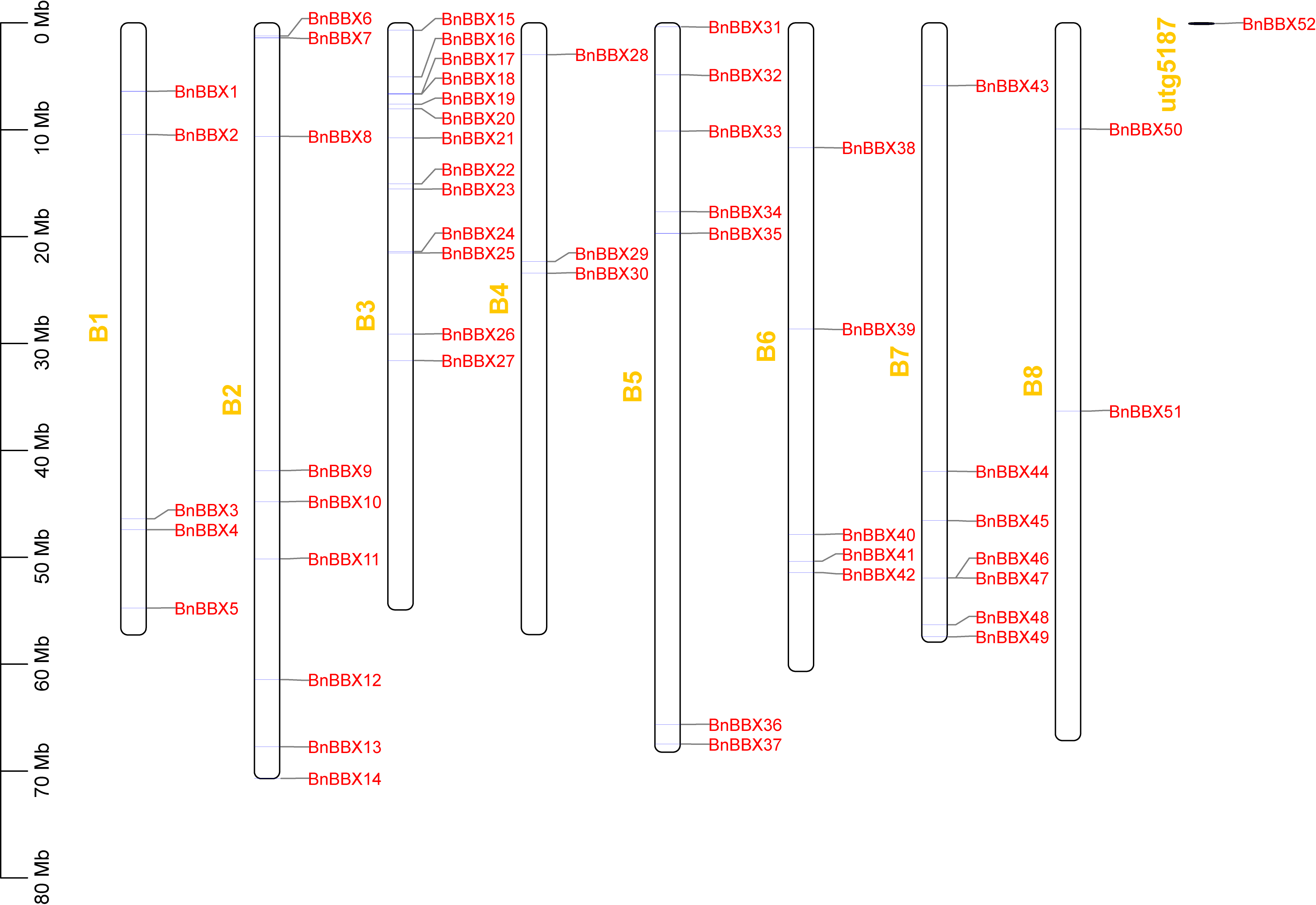


Fig. S1-7
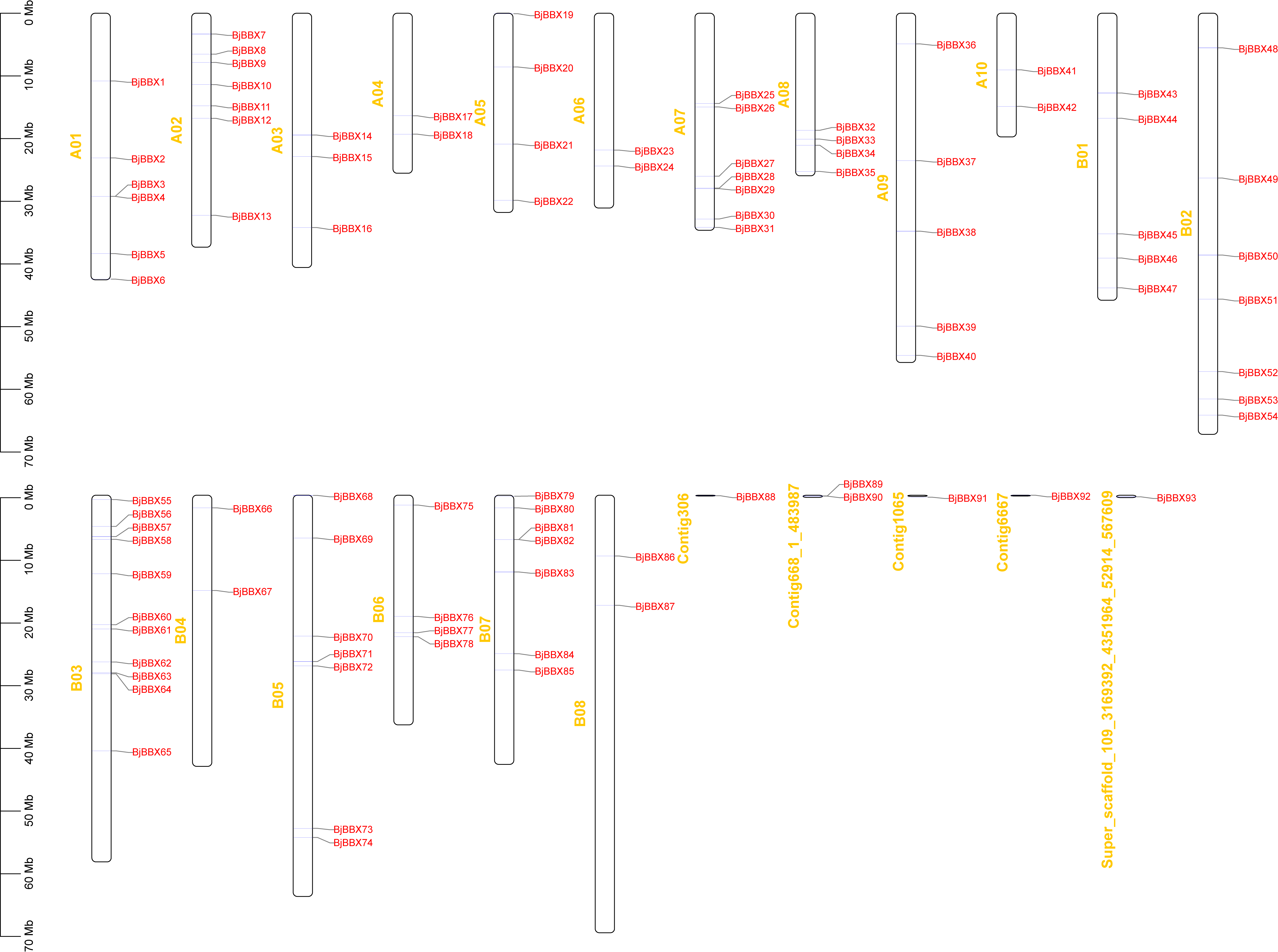


Fig. S1-8


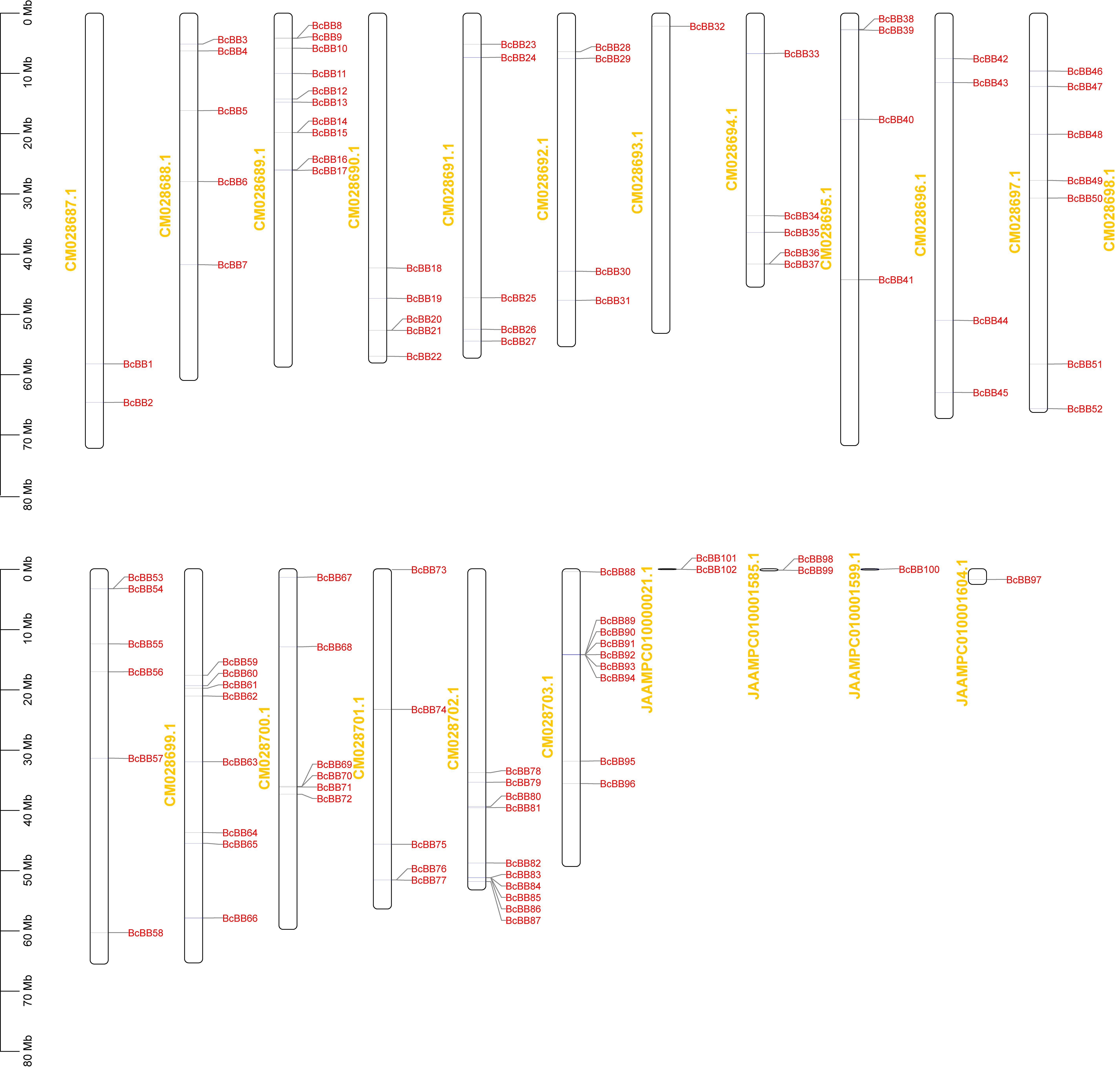

Supplement: Supplementary file 1 — Additional file 1: Figure S1. Chromosomal location of BBX genes on Brassicaceae chromosomes. Figure S1–1 The location of BBX genes on B. napus chromosomes. Figure S1–2 The location of BBX genes on B. oleracea chromosomes. Figure S1–3 The location of BBX genes on B. rapa chromosomes and scaffold. Figure S1–4 The location of BBX genes on C. rubella scaffolds. Figure S1–5 The location of BBX genes on C. sativa chromosomes and scaffold. Figure S1–6 Chromosomal location of B. nigra BBX genes. Figure S1–7 Chromosomal location of B.juncea BBX genes. Figure S1–8 Chromosomal location of B. carinata BBX genes. [file 12870_2021_3043_MOESM1_ESM.docx]

Fig. S2-1


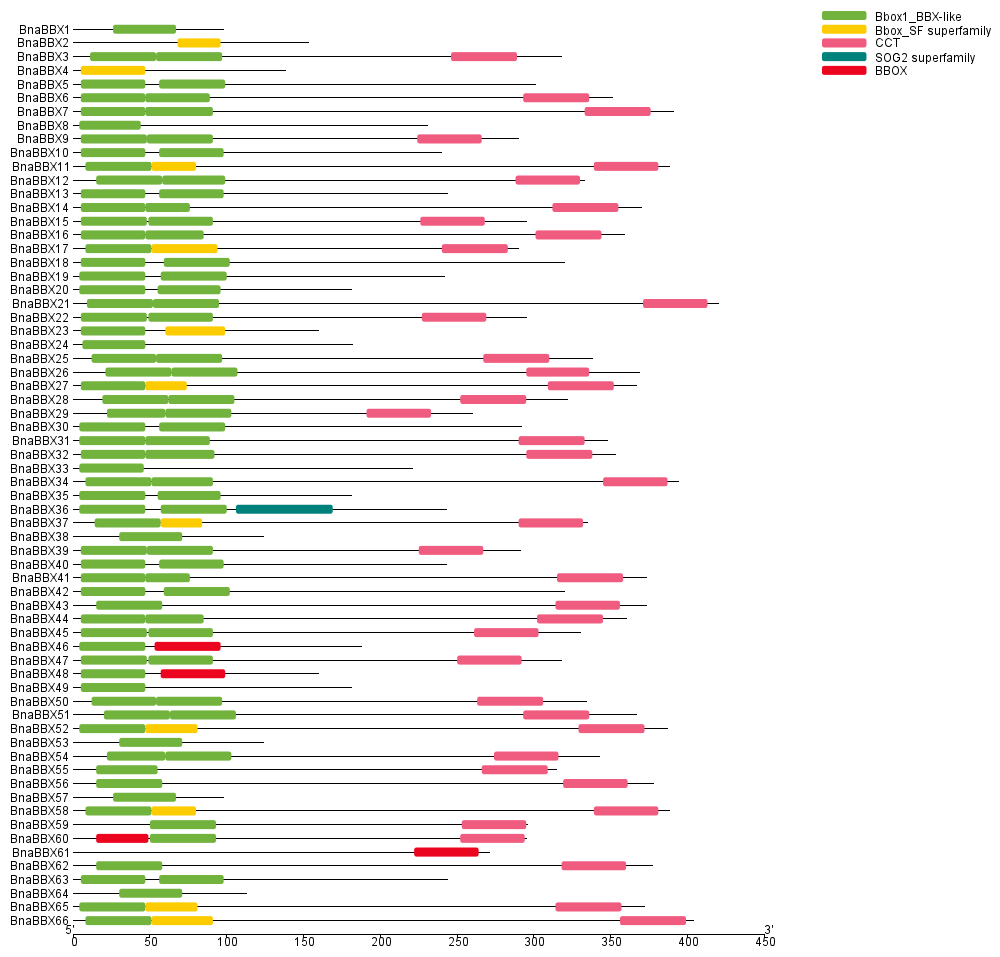


Fig. S2-2


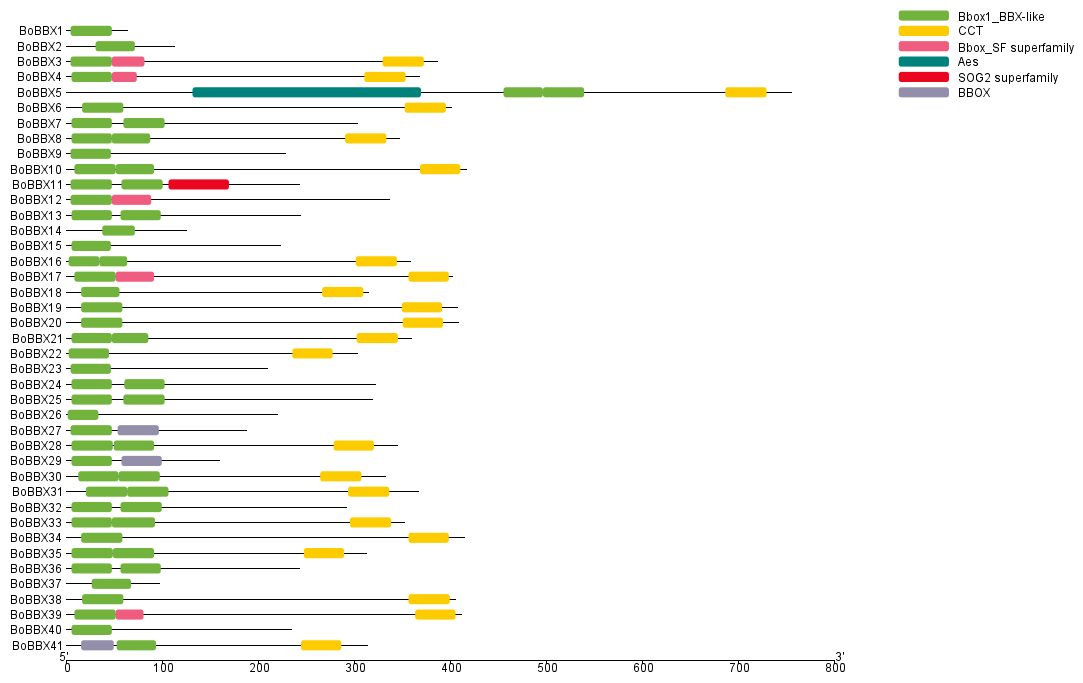


Fig. S2-3


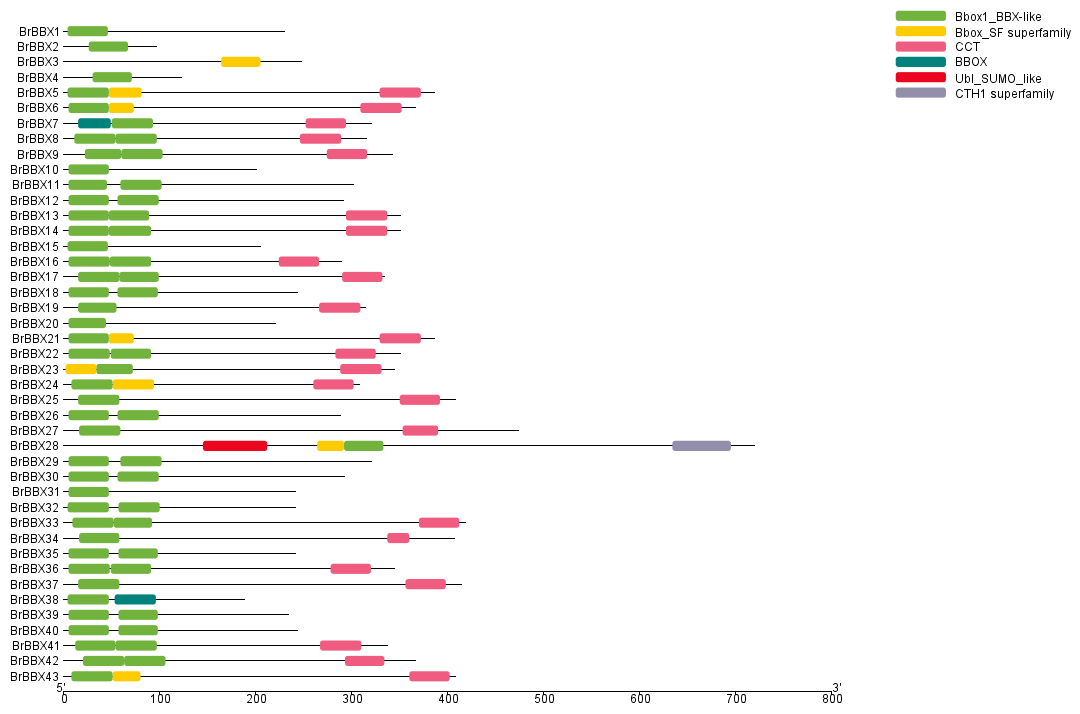


Fig. S2-4


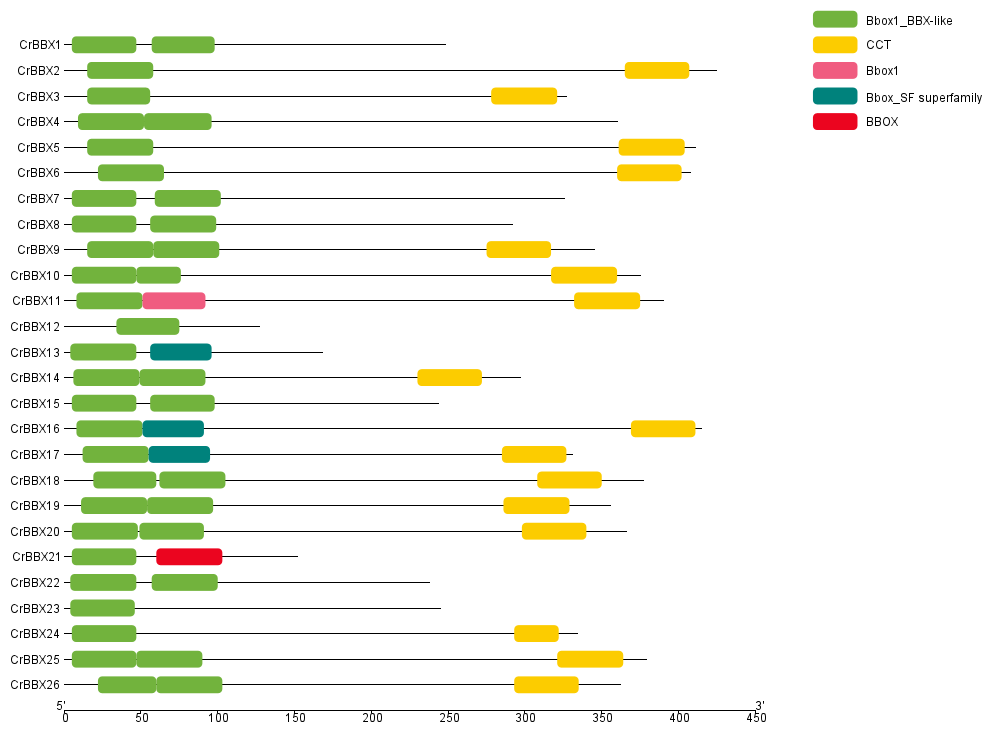


Fig. S2-5


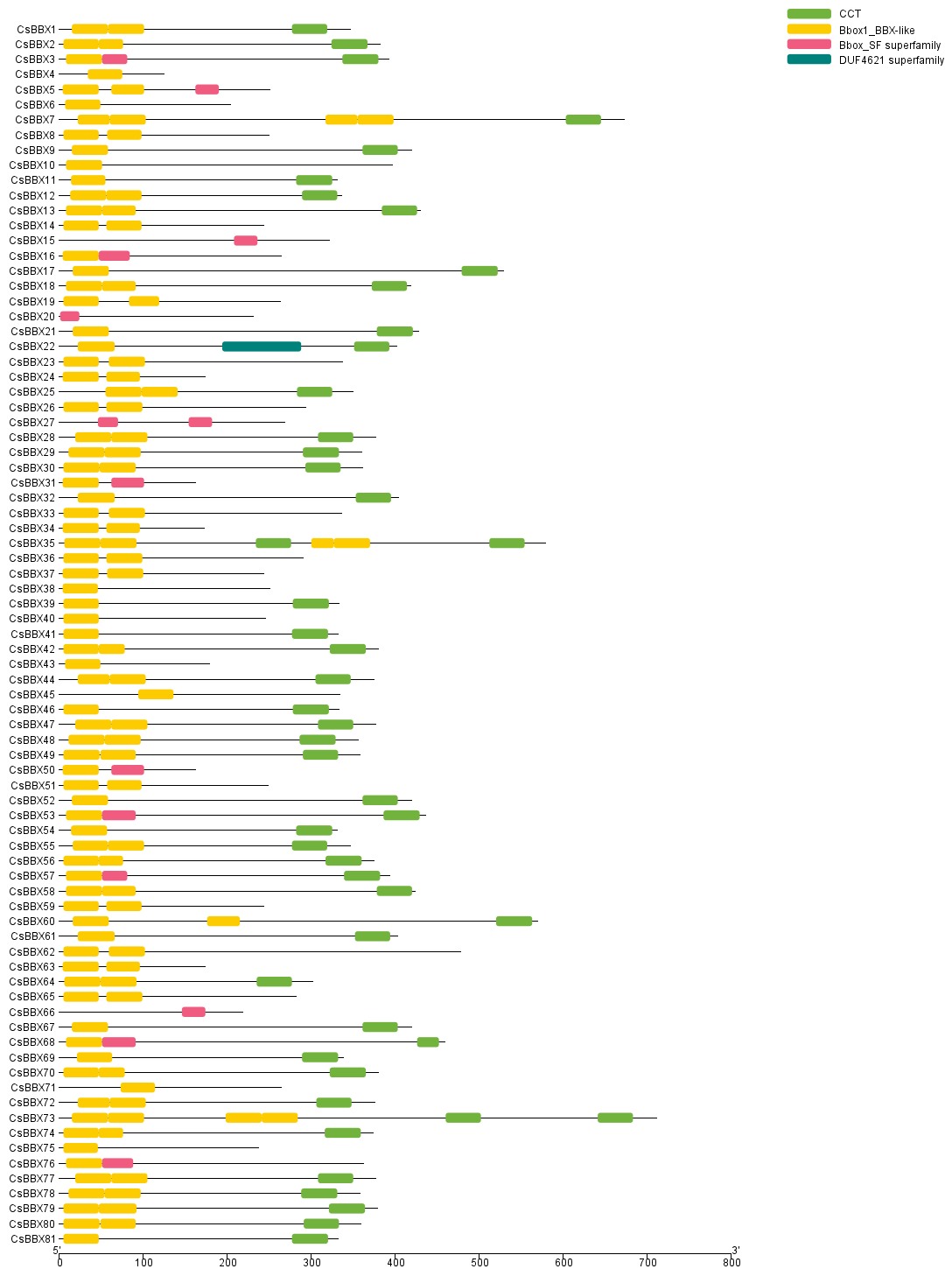


Fig. S2-6
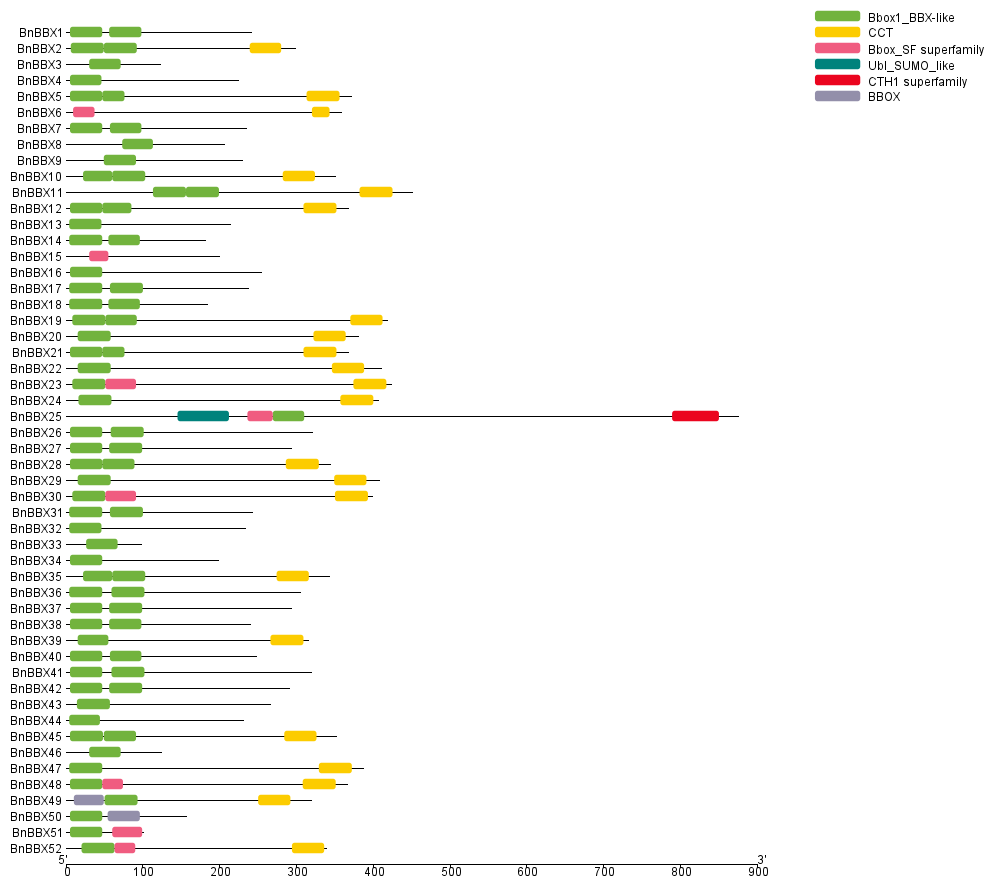


Fig. S2-7
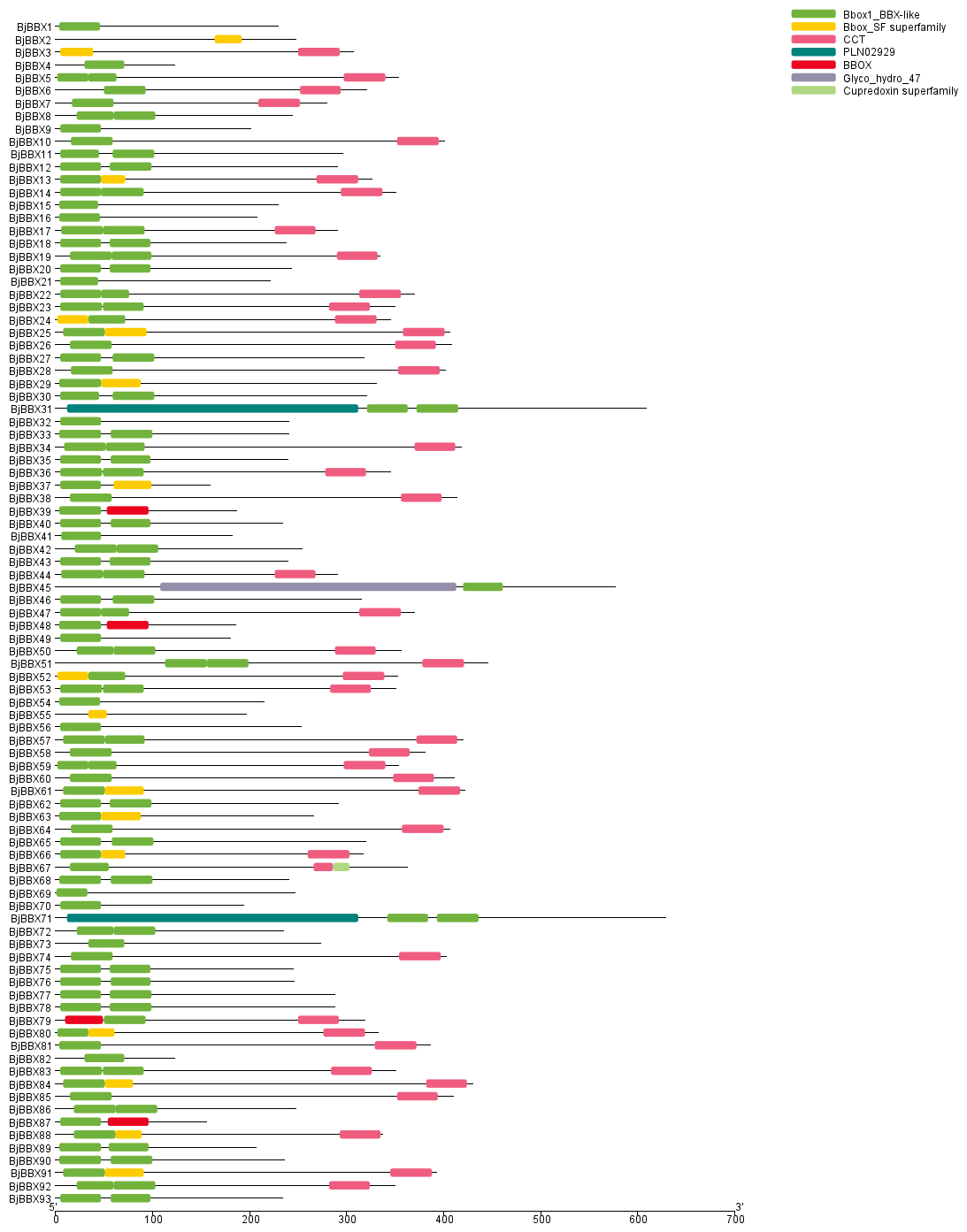


Fig. S2-8


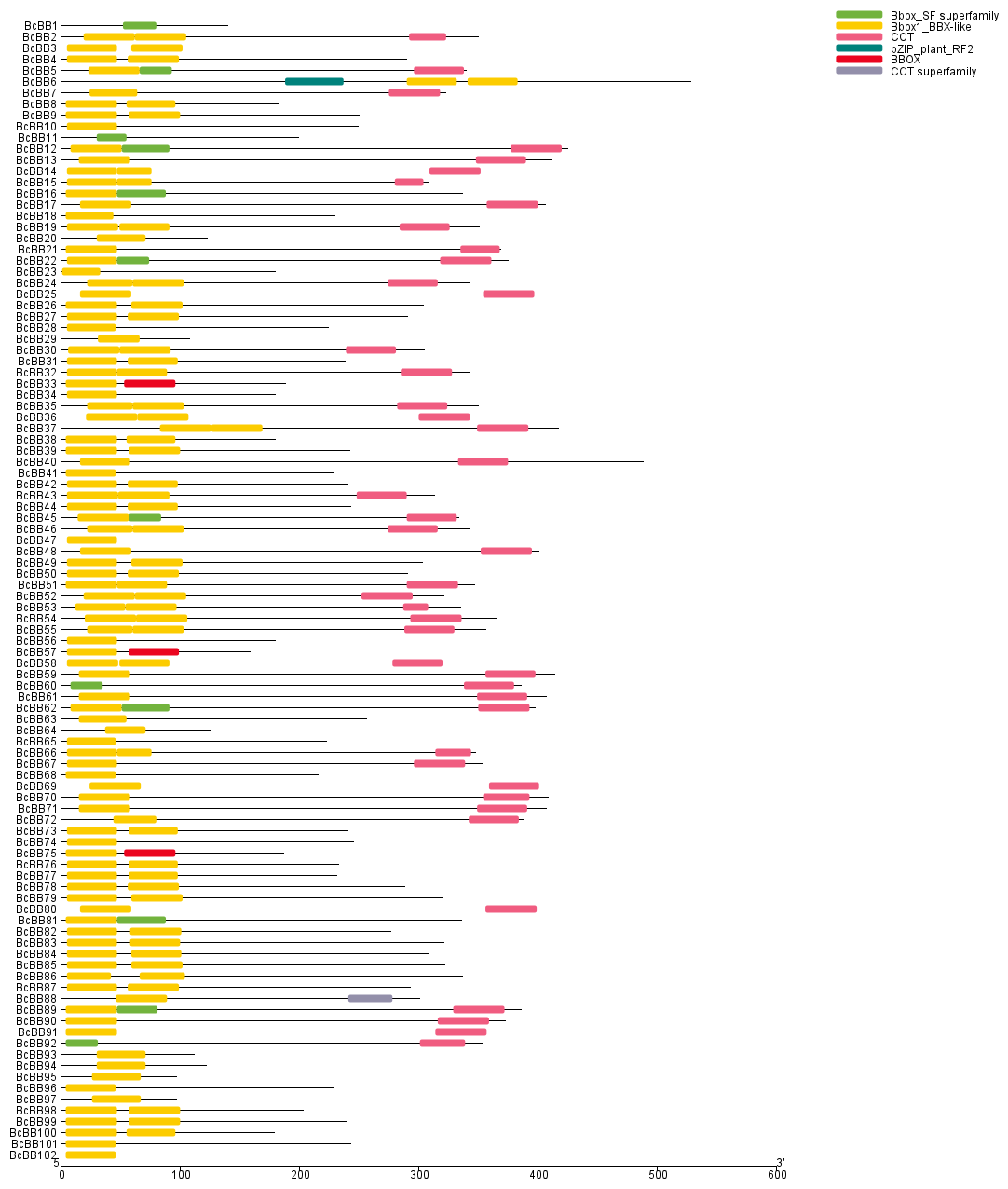

Supplement: Supplementary file 2 — Additional file 2: Figure S2. Identification and characterization of conserved domains in BBXs in NCBI database. Figure S2–1 Identification and characterization of the conserved domains in BnaBBXs. Figure S2–2 Identification and characterization of the conserved domains in BoBBXs. Figure S2–3 Identification and characterization of the conserved domains in BrBBXs. Figure S2–4 Identification and characterization of the conserved domains in CrBBXs. Figure S2–5 Identification and characterization of the conserved domains in CsBBXs. Figure S2–6 Identification and characterization of the conserved domains in BnBBXs. Figure S2–7 Identification and characterization of the conserved domains in BjBBXs. Figure S2–8 Identification and characterization of the conserved domains in BcBBXs. [file 12870_2021_3043_MOESM2_ESM.docx]

Fig. S3-1


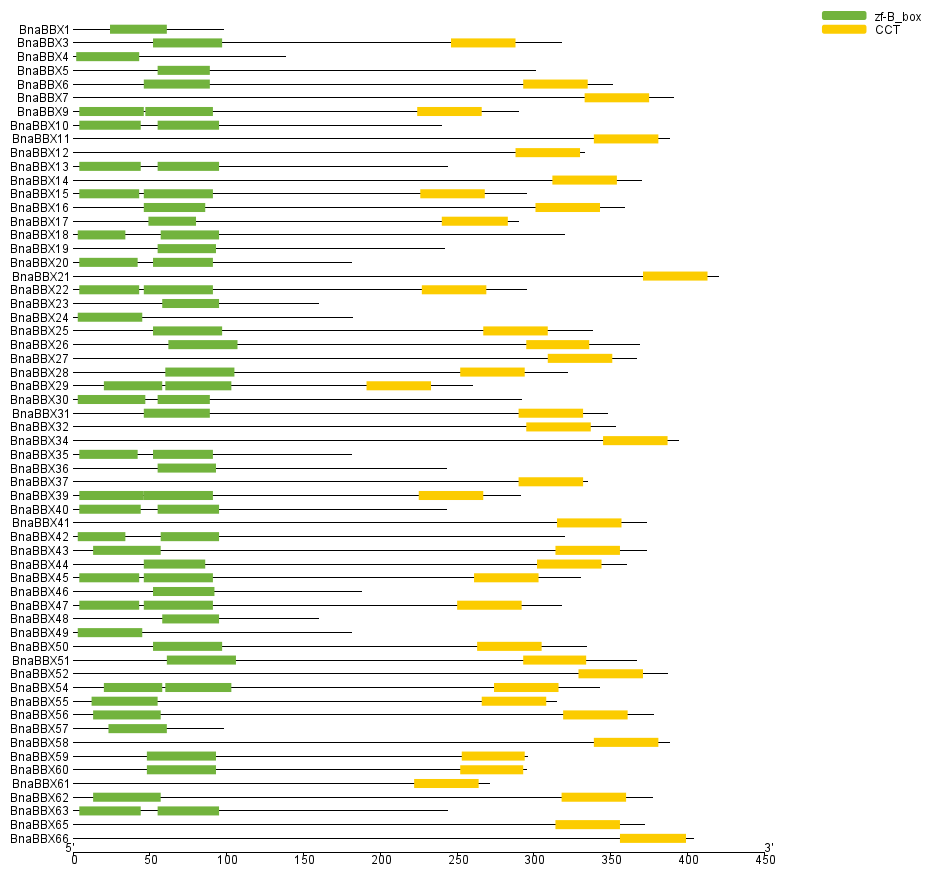


Fig. S3-2


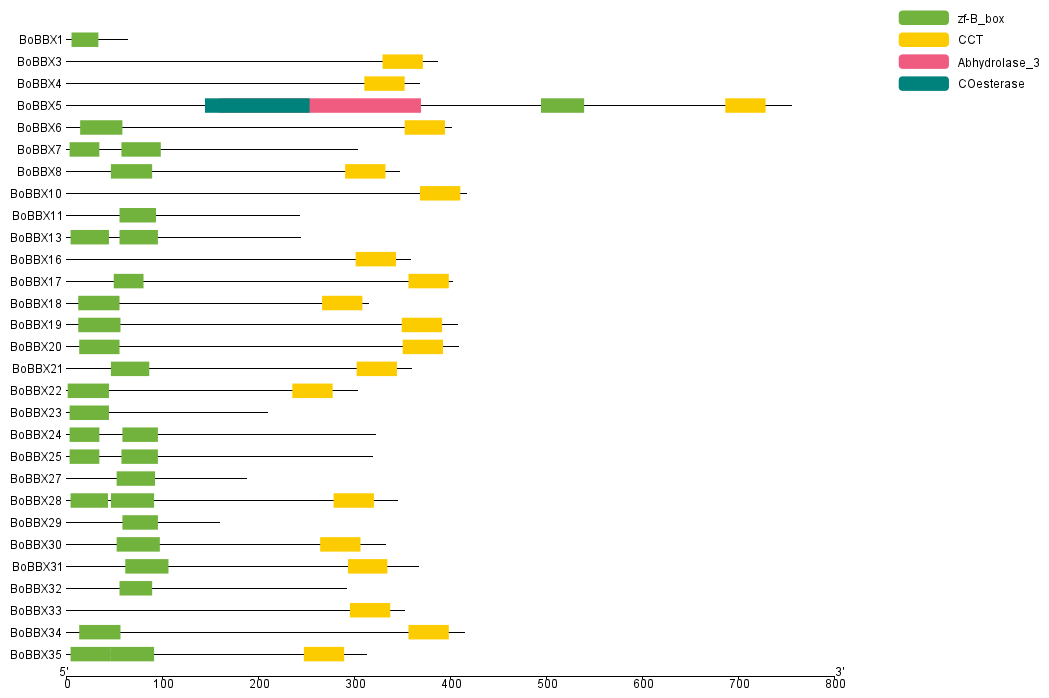


Fig. S3-3


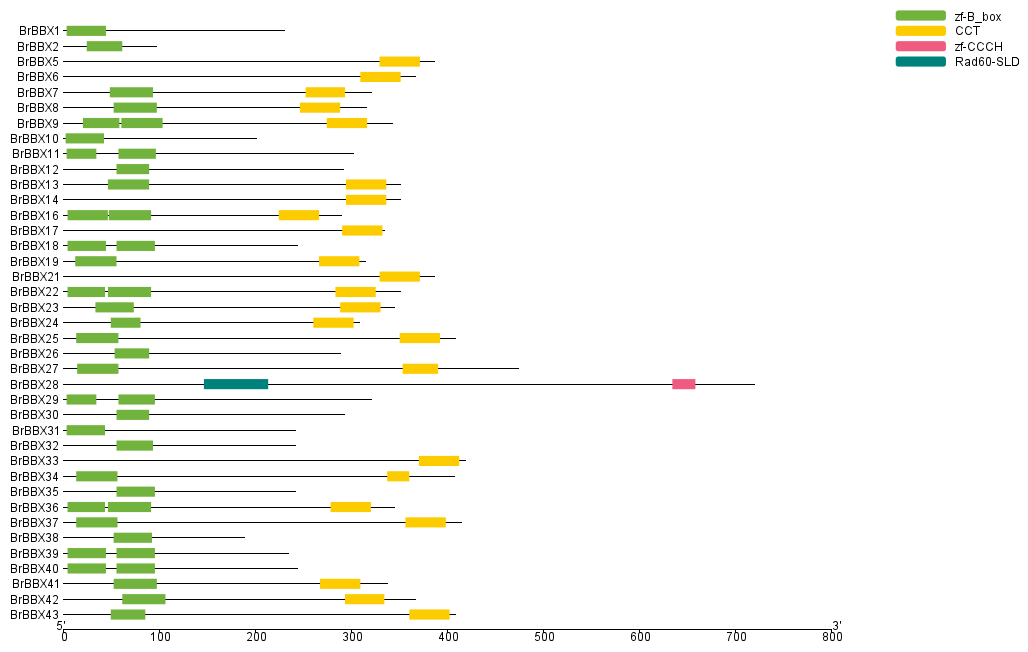


Fig. S3-4


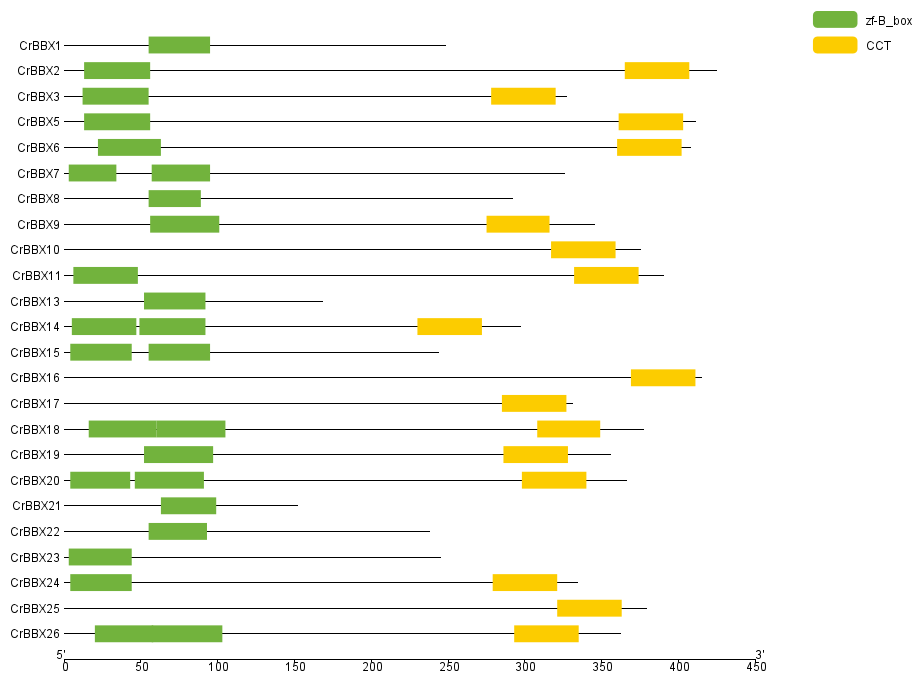


Fig. S3-5


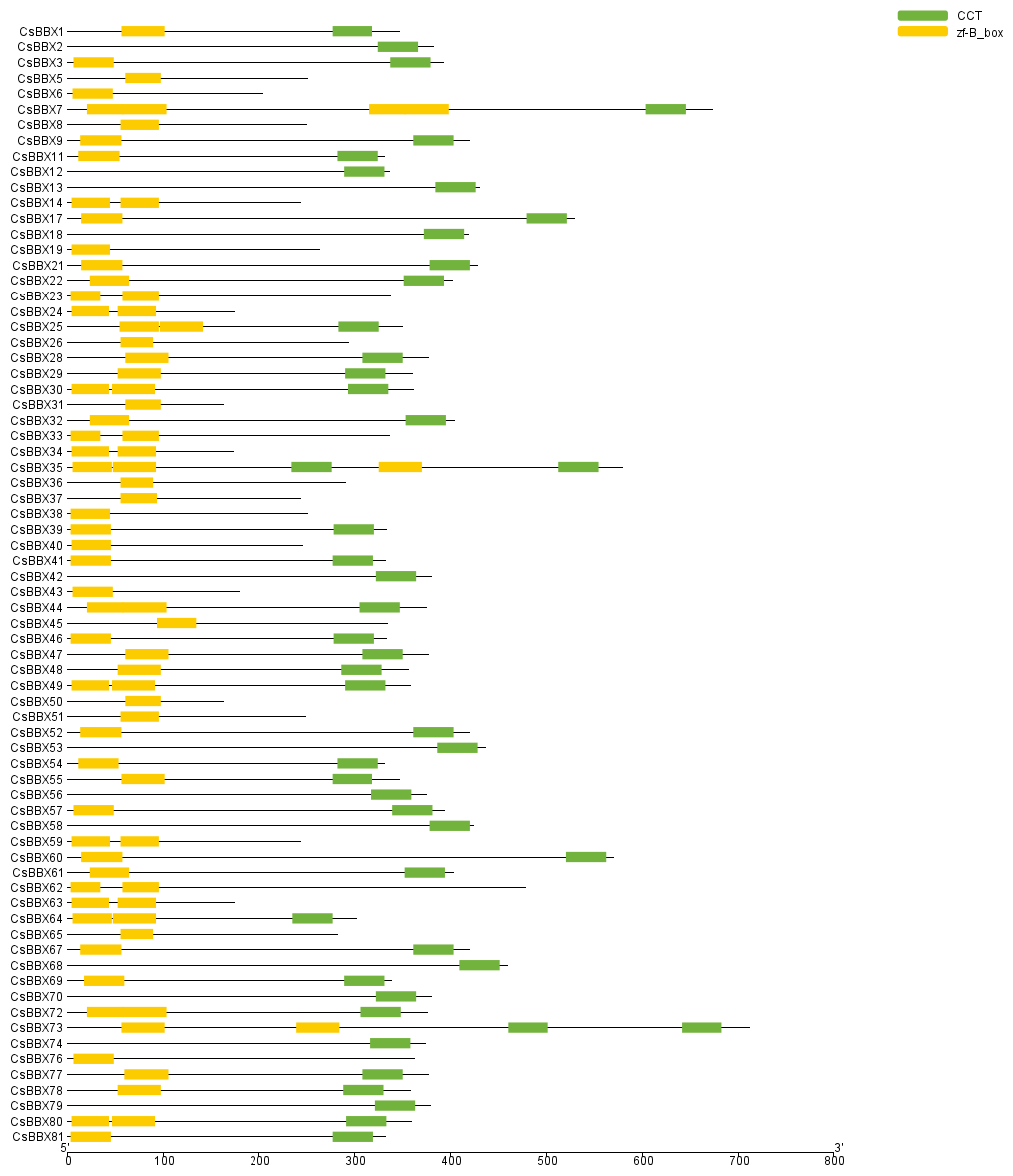


Fig. S3-6
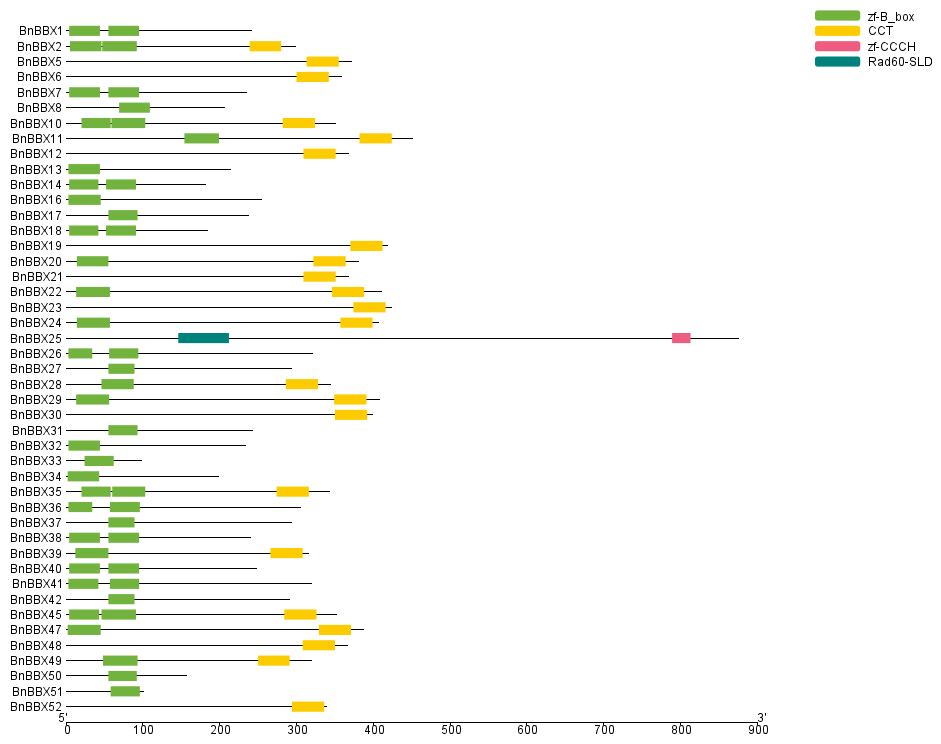


Fig. S3-7


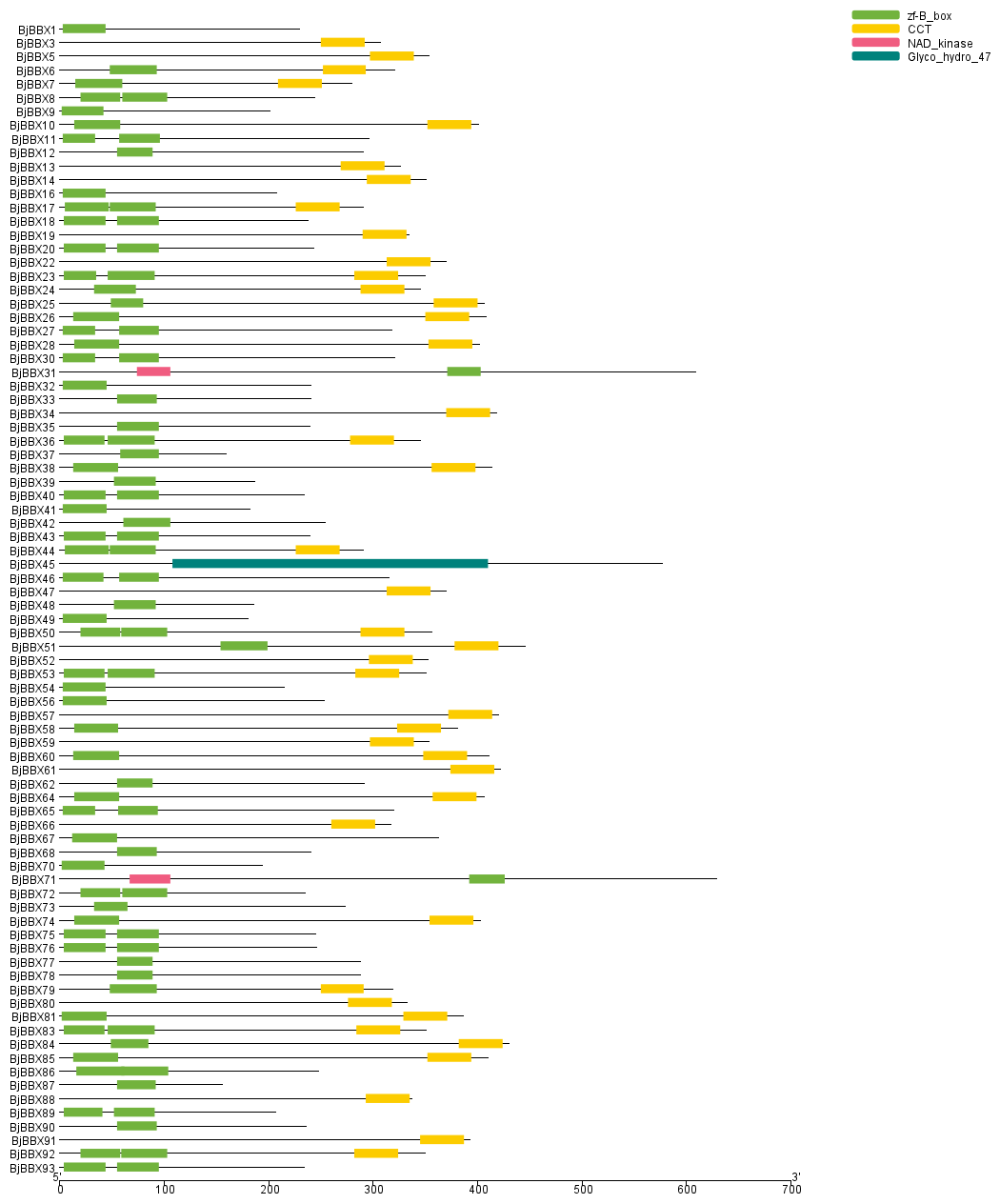


Fig. S3-8


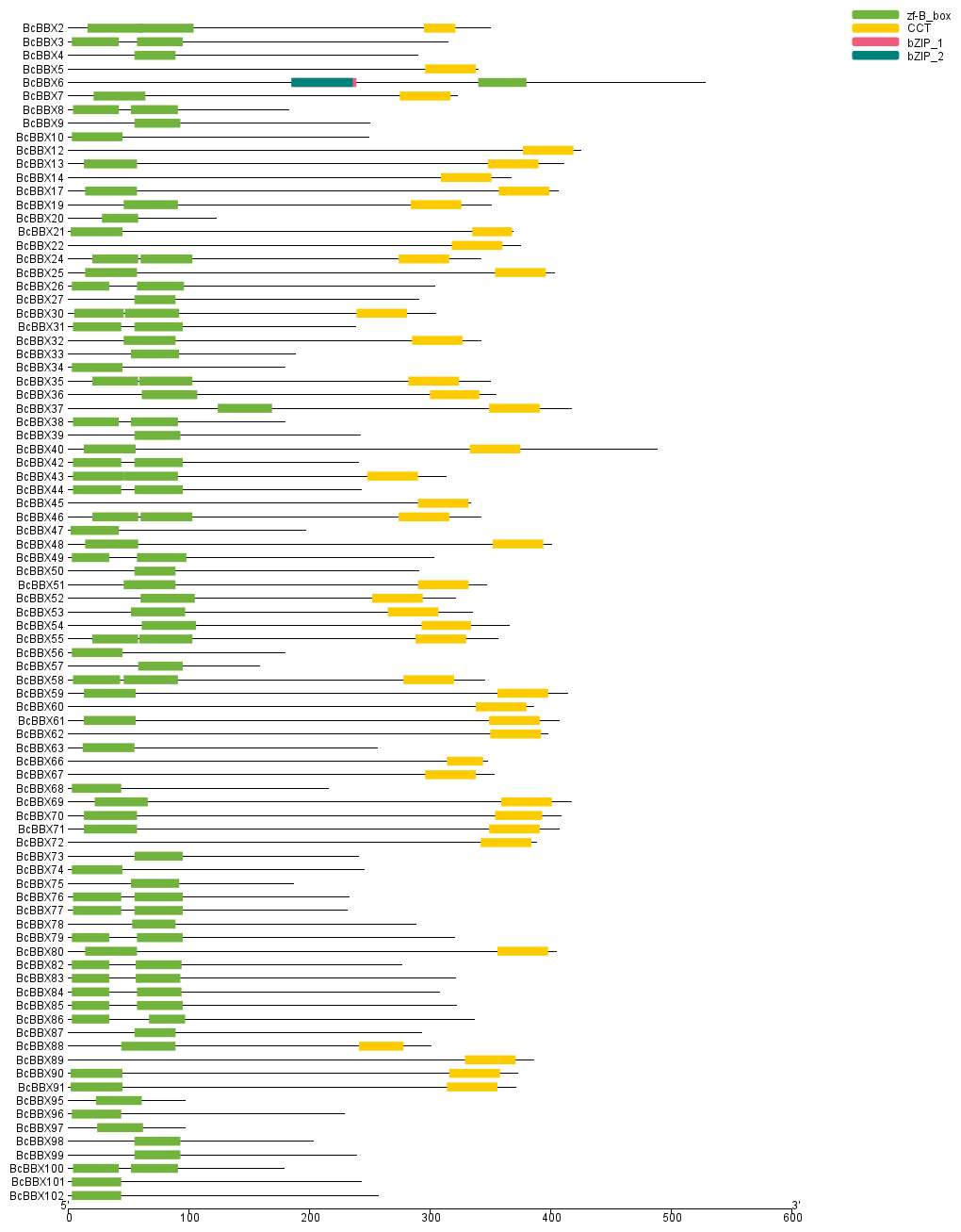

Supplement: Supplementary file 3 — Additional file 3: Figure S3. Identification and characterization of conserved domains in BBXs in pfam database. Figure S3–1 Identification and characterization of the conserved domains in BnaBBXs. Figure S3–2 Identification and characterization of the conserved domains in BoBBXs. Figure S3–3 Identification and characterization of the conserved domains in BrBBXs. Figure S3–4 Identification and characterization of the conserved domains in CrBBXs. Figure S3–5 Identification and characterization of the conserved domains in CsBBXs. Figure S3–6 Identification and characterization of the conserved domains in BnBBXs. Figure S3–7 Identification and characterization of the conserved domains in BjBBXs. Figure S3–8 Identification and characterization of the conserved domains in BcBBXs. [file 12870_2021_3043_MOESM3_ESM.docx]

Fig. S4-1


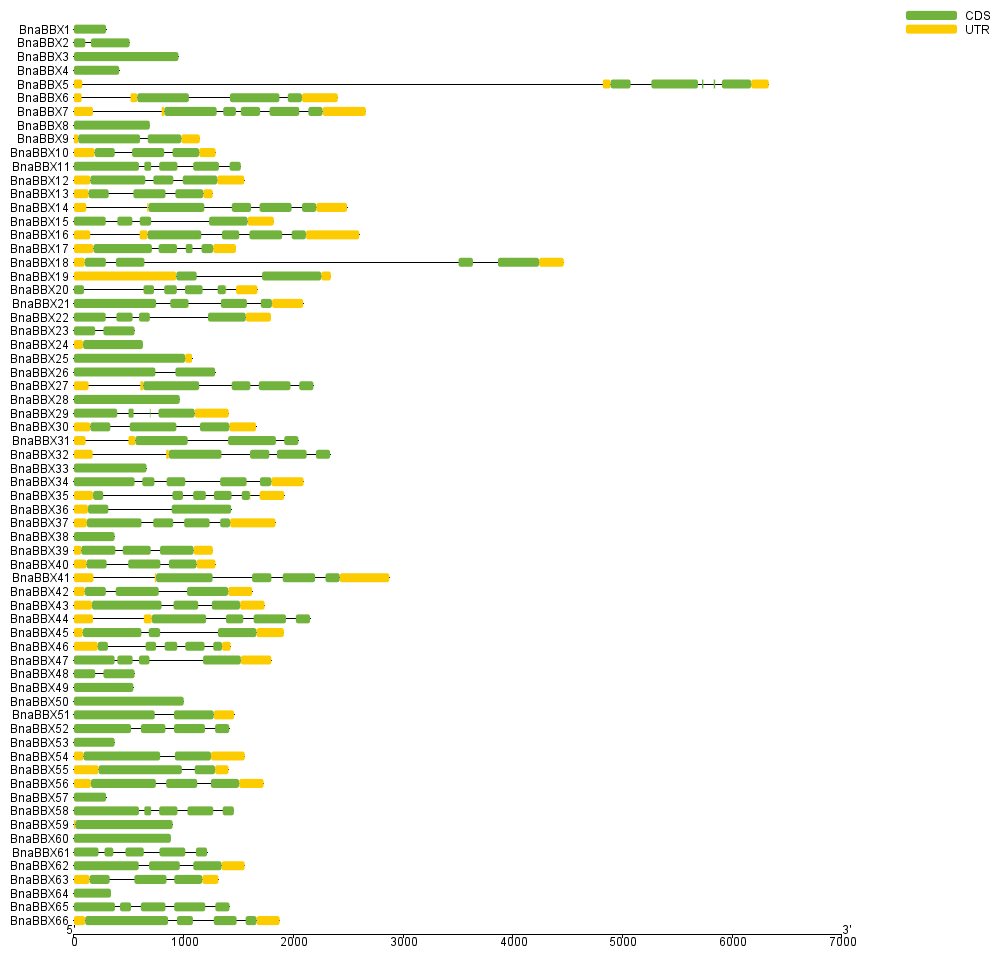


Fig. S4-2


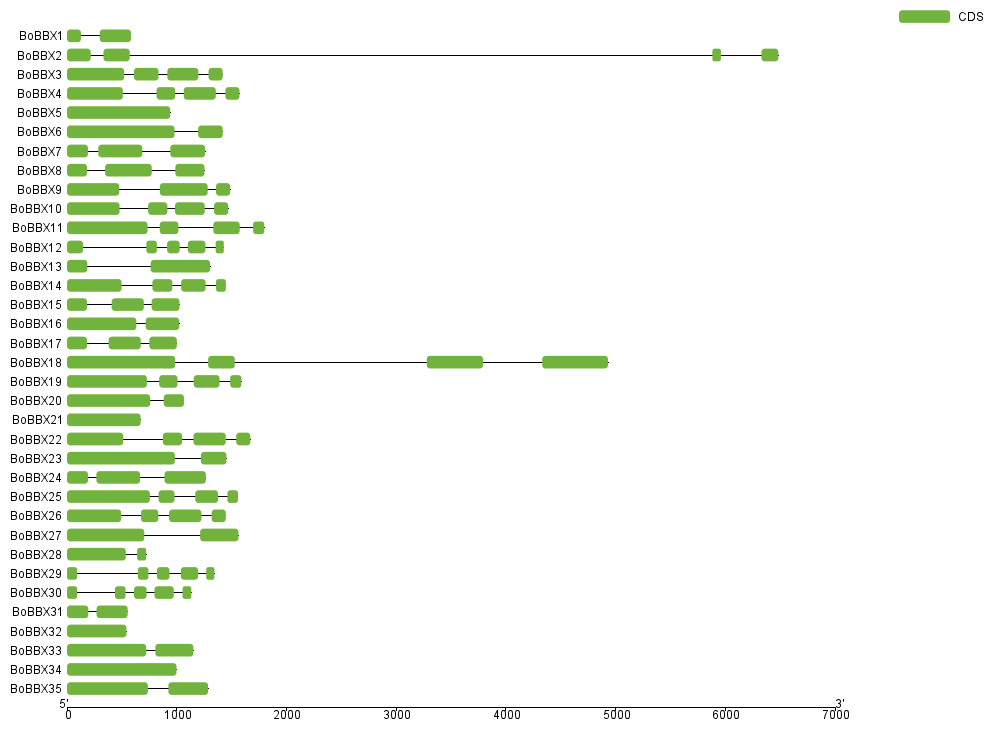


Fig. S4-3


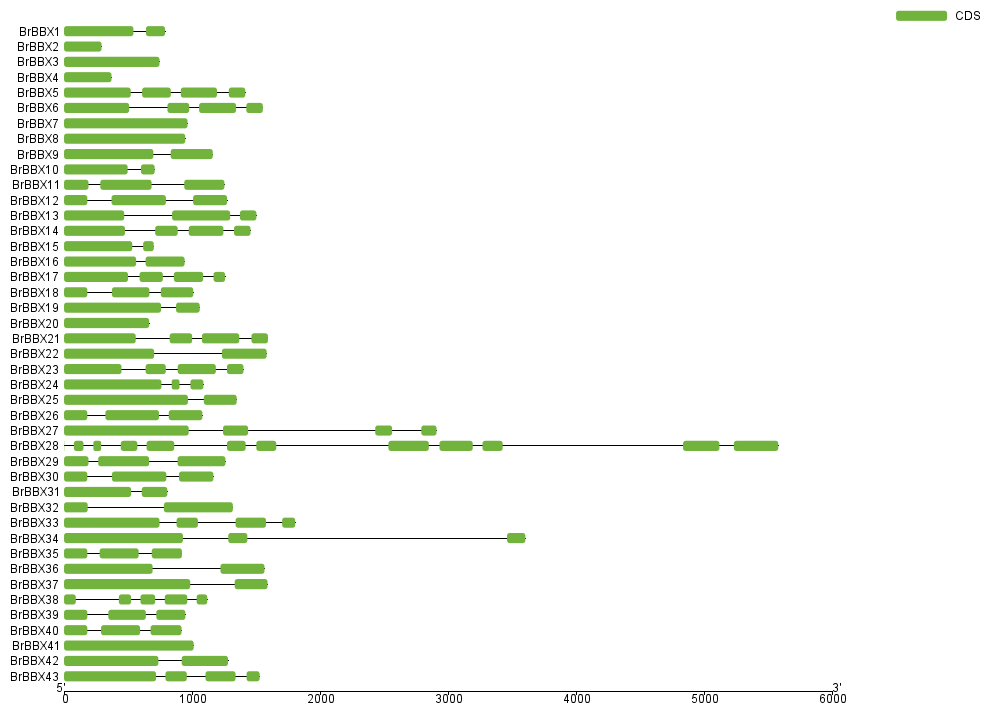


Fig. S4-4


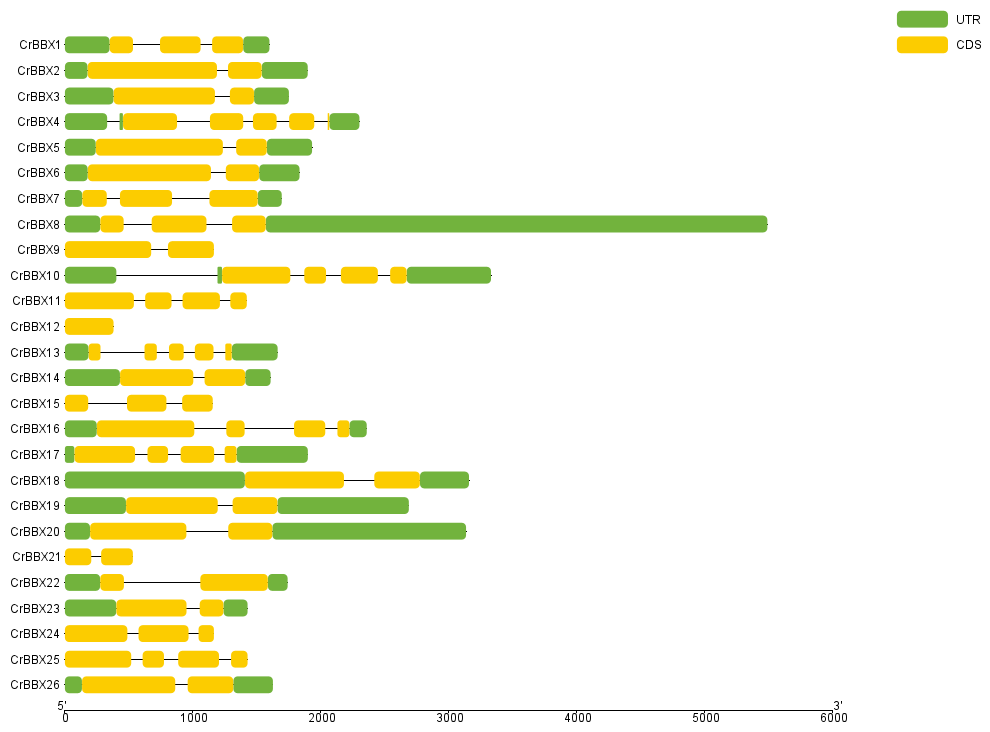


Fig. S4-5


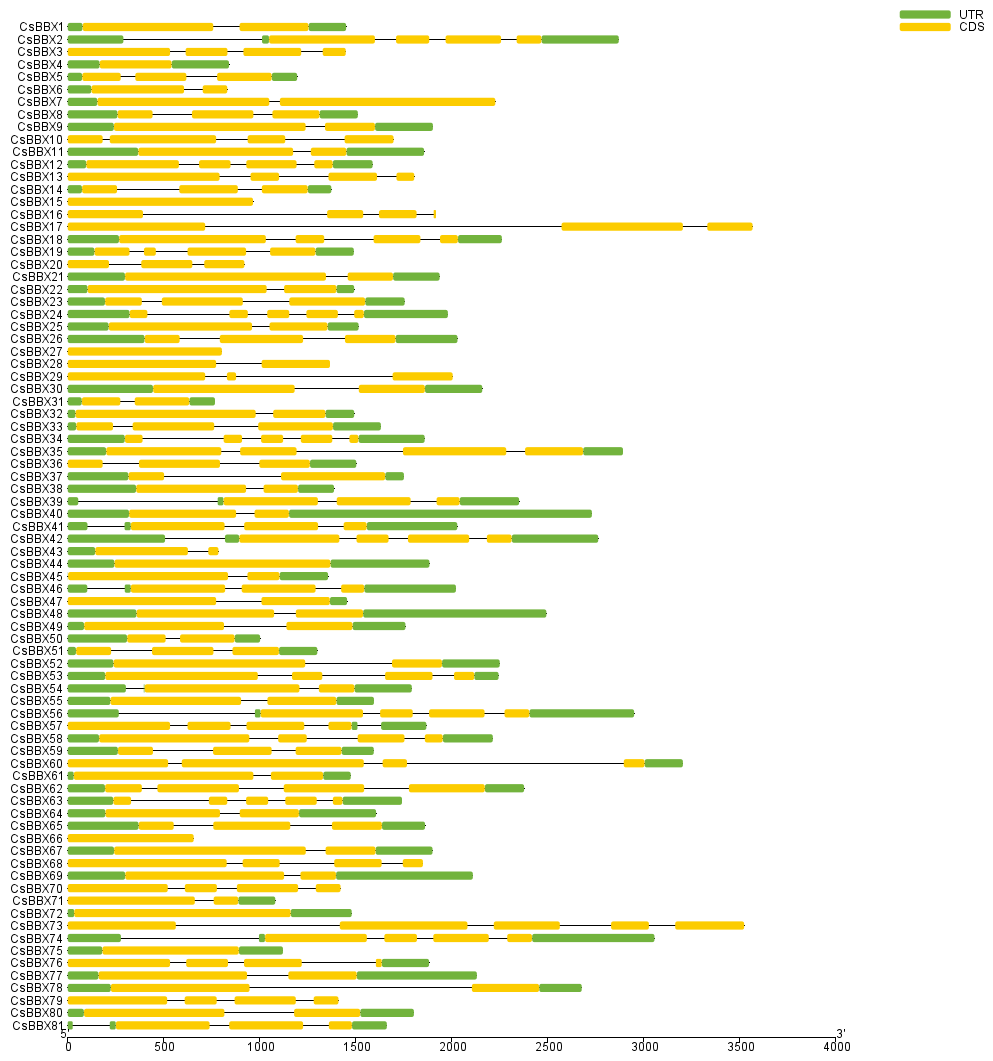


Fig. S4-6
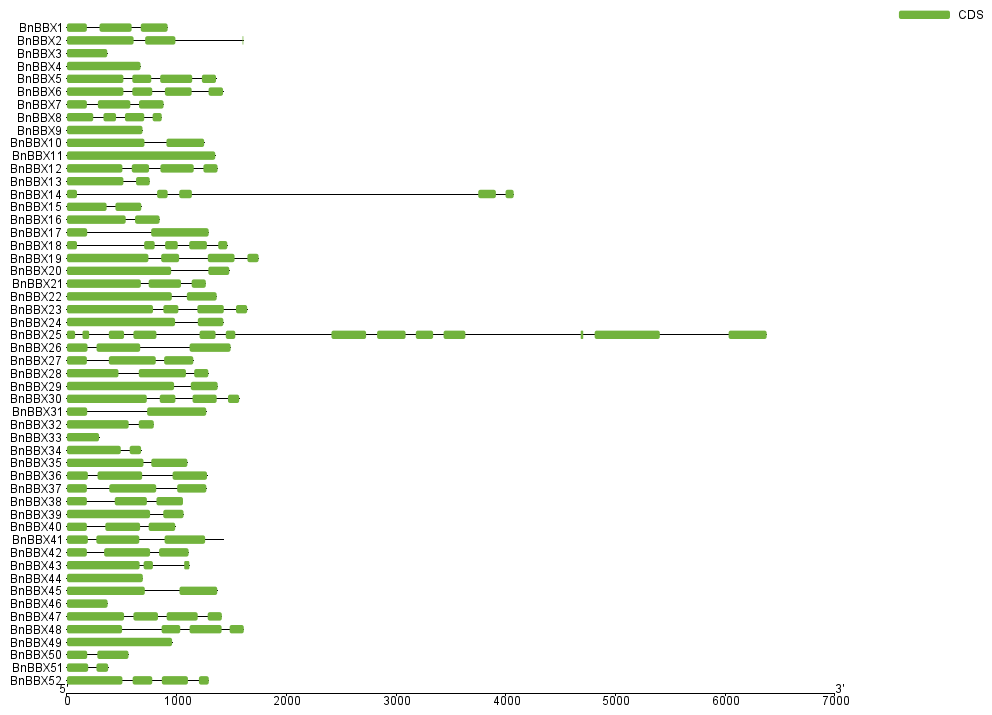


Fig. S4-7


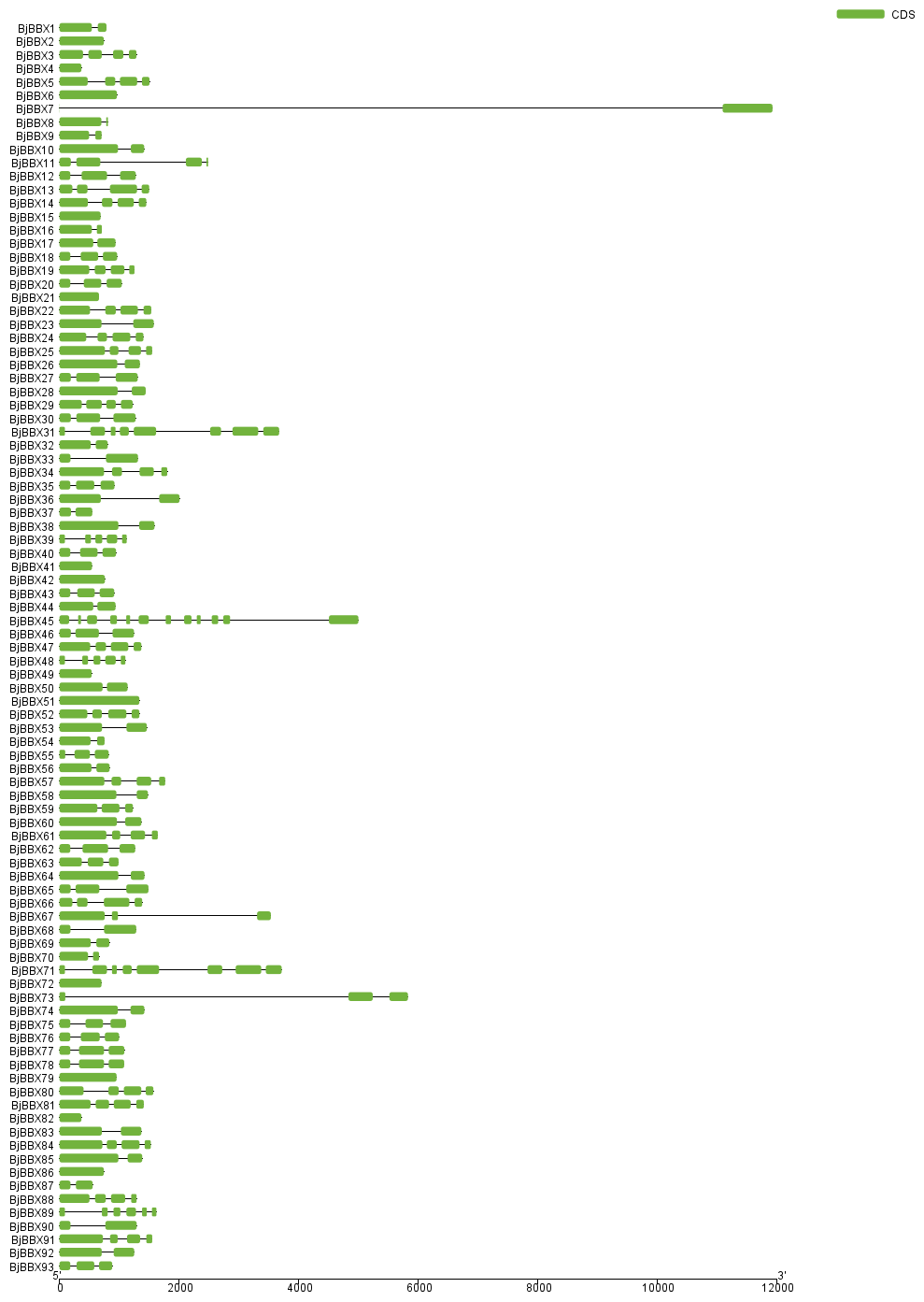


Fig. S4-8
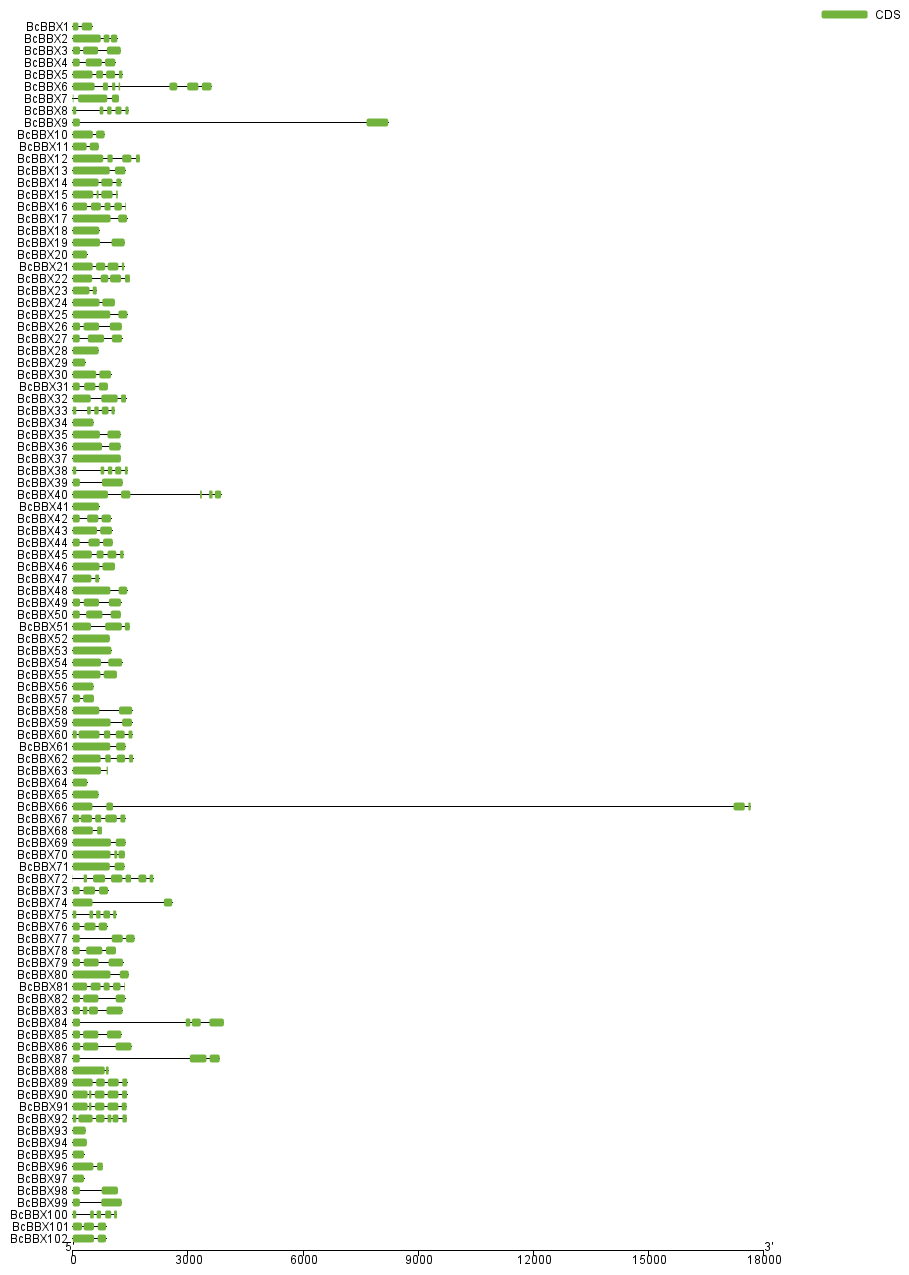

Supplement: Supplementary file 4 — Additional file 4: Figure S4. Structure analysis of BBX genes. Figure S4–1 Exon–intron structures of BnaBBX genes. Figure S4–2 Exon–intron structures of BoBBX genes. Figure S4–3 Exon–intron structures of BrBBX genes. Figure S4–4 Exon–intron structures of CrBBX genes. Figure S4–5 Exon–intron structures of CsBBX genes. Figure S4–6 Exon–intron structures of BnBBX genes. Figure S4–7 Exon–intron structures of BjBBX genes. Figure S4–8 Exon–intron structures of BcBBX genes. [file 12870_2021_3043_MOESM4_ESM.docx]

Fig. S5-1


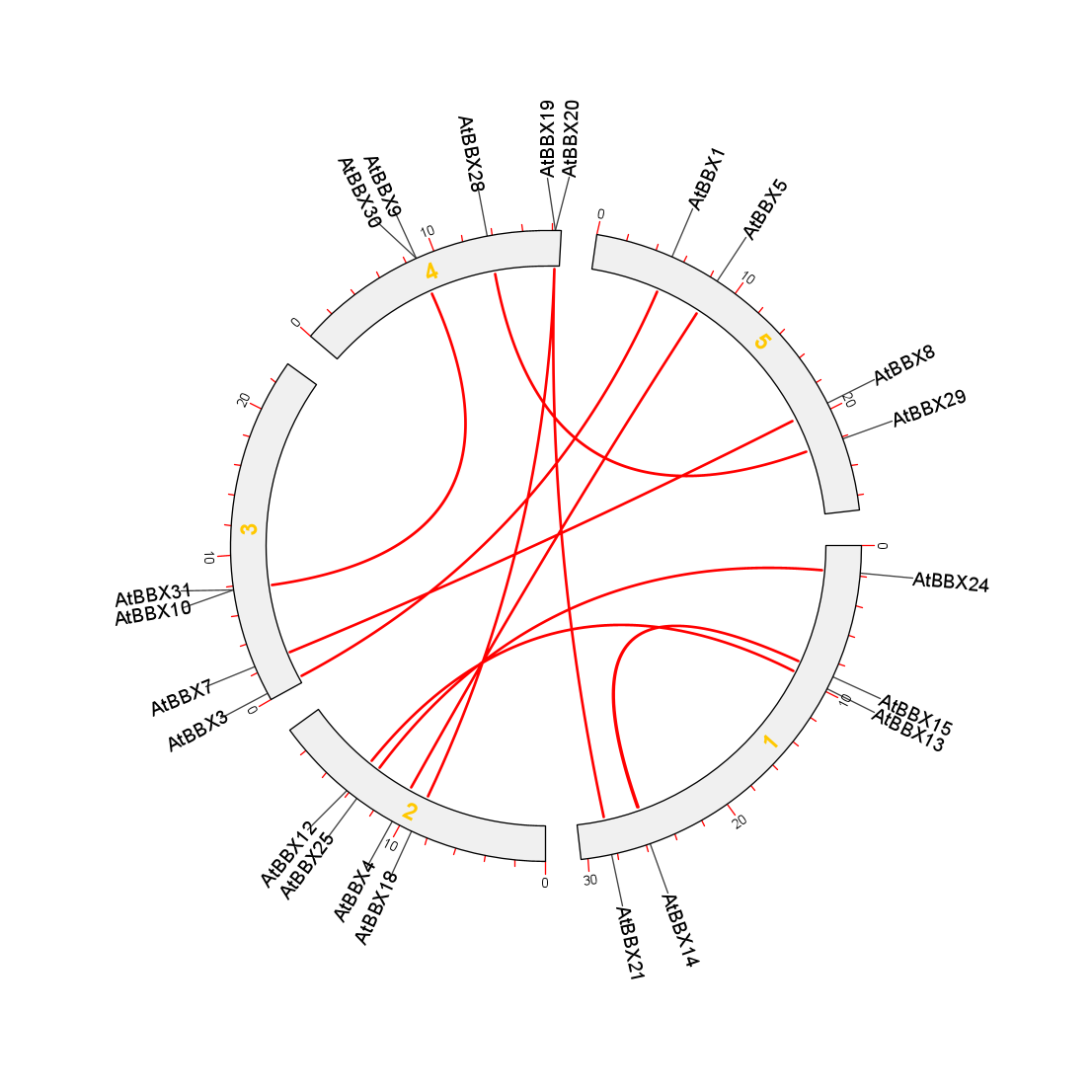


Fig. S5-2


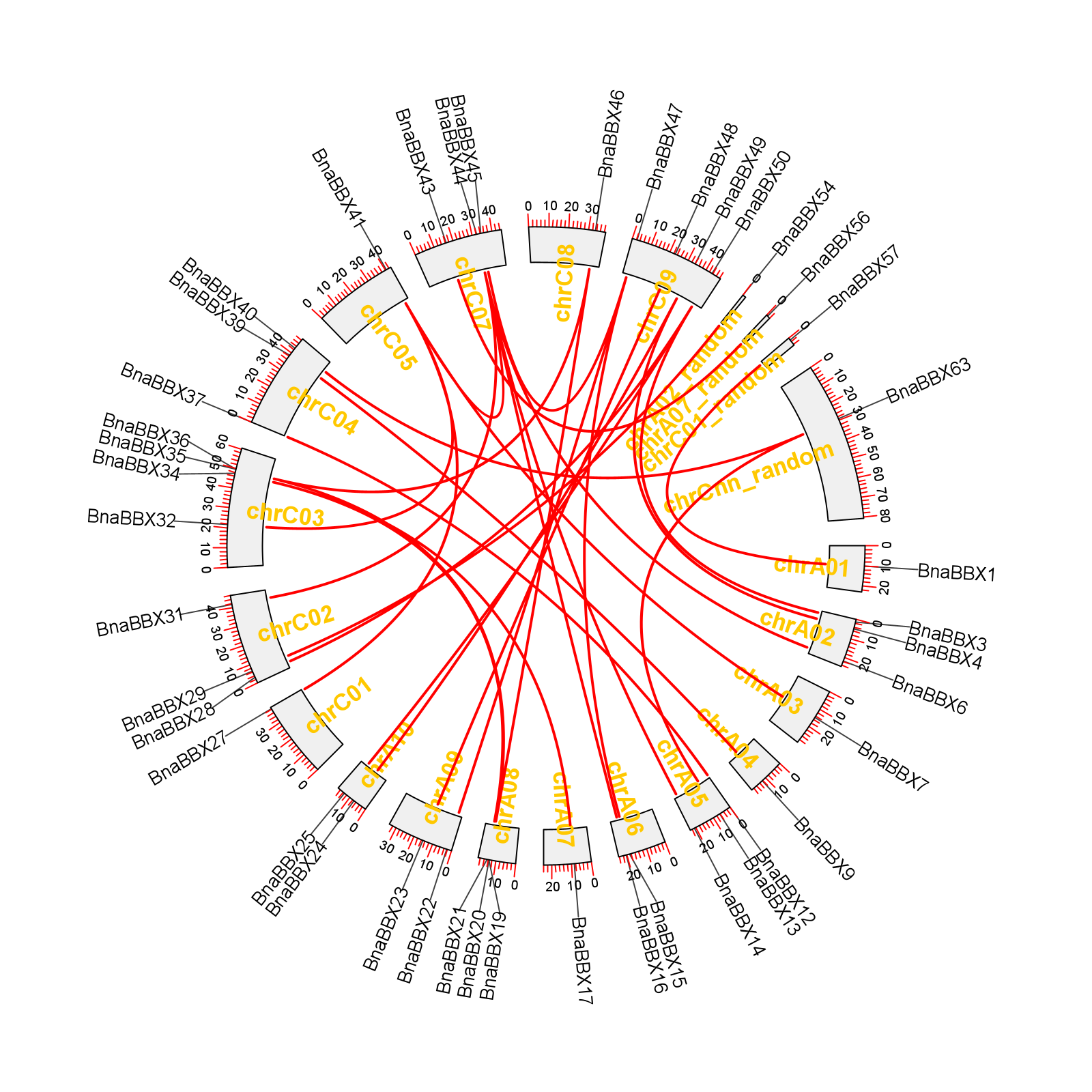


Fig. S5-3


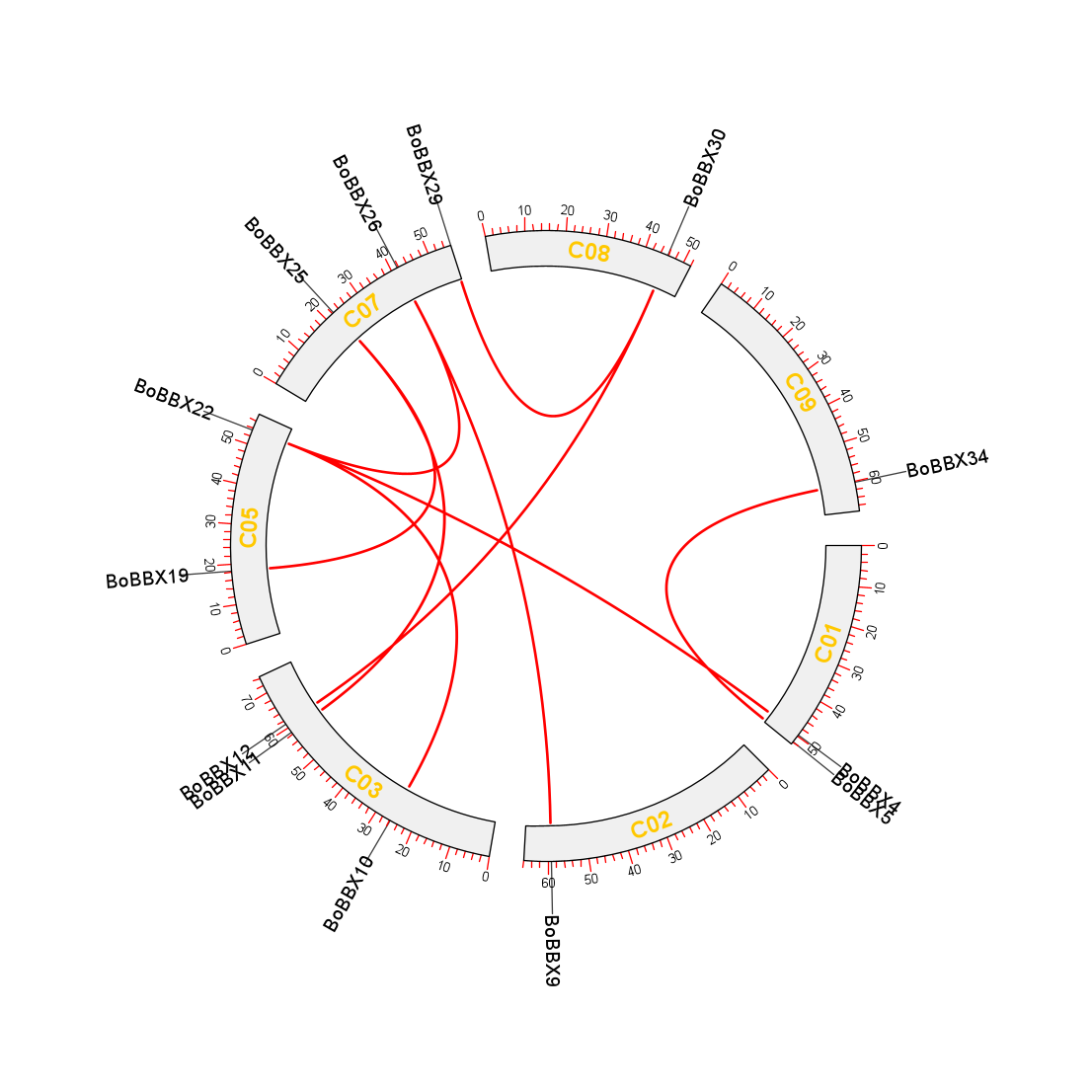


Fig. S5-4


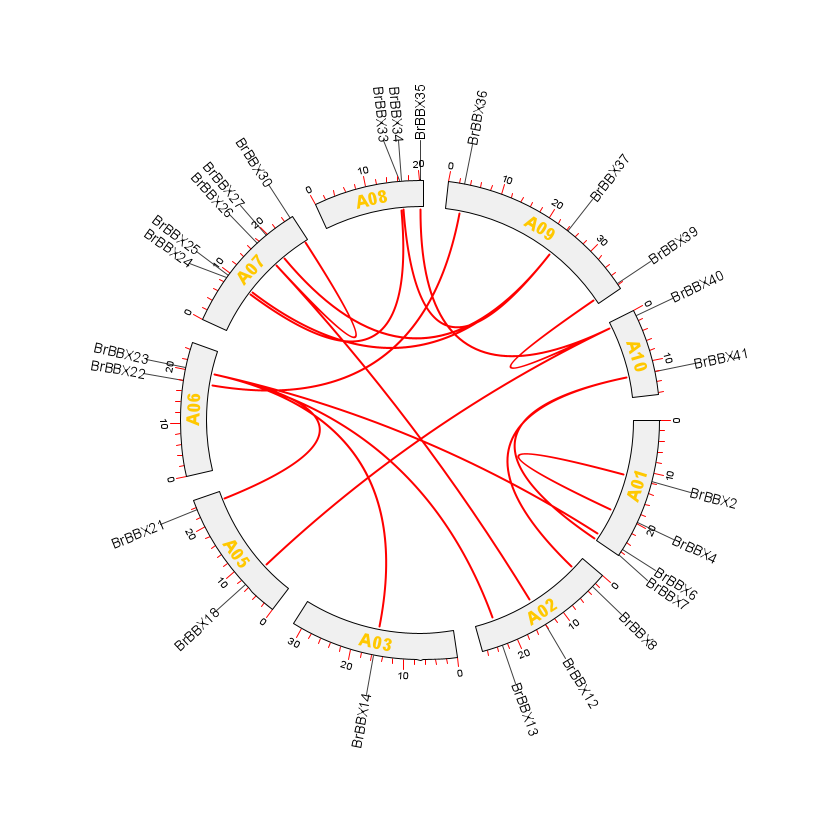


Fig. S5-5


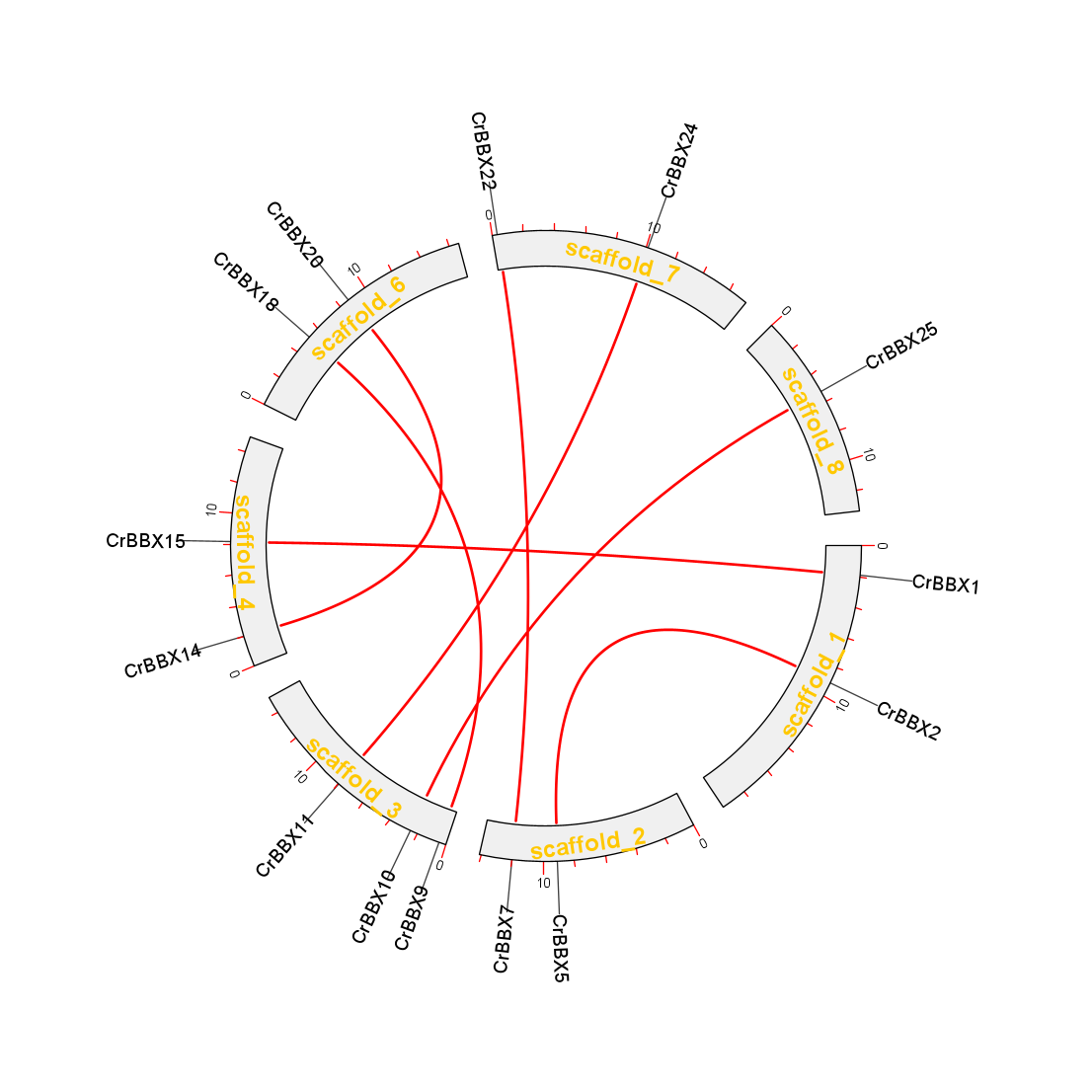


Fig. S5-6


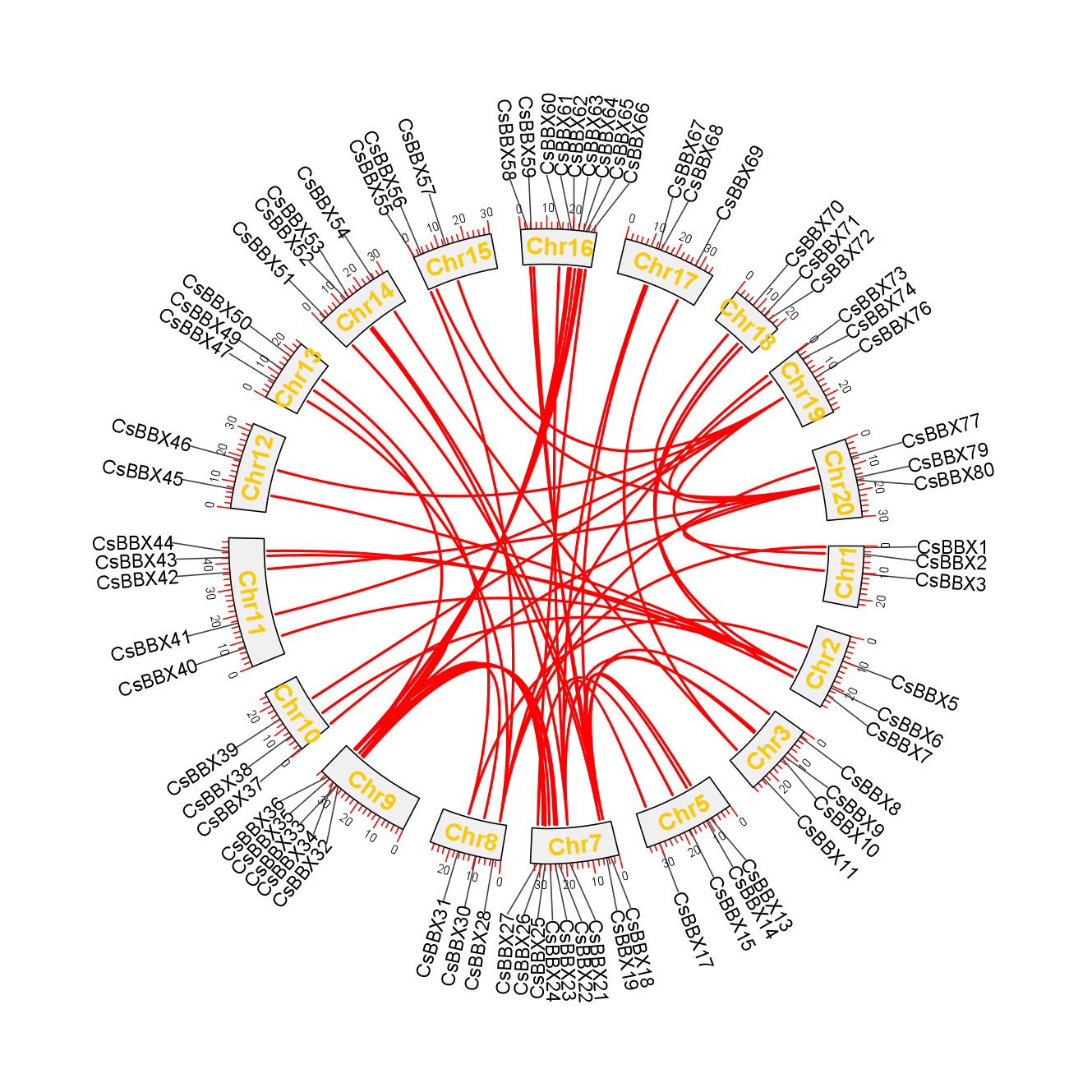


Fig. S5-7
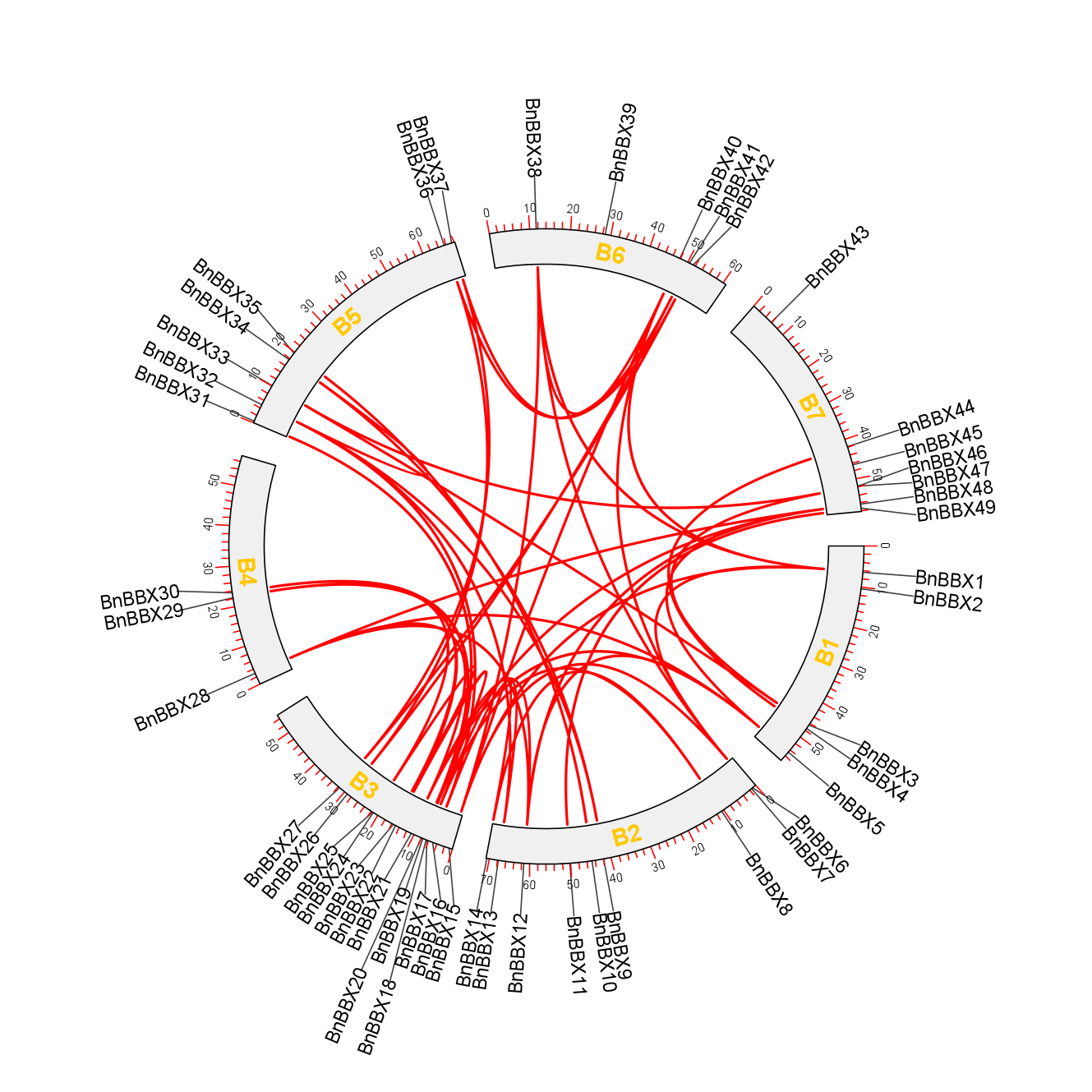


Fig. S5-8


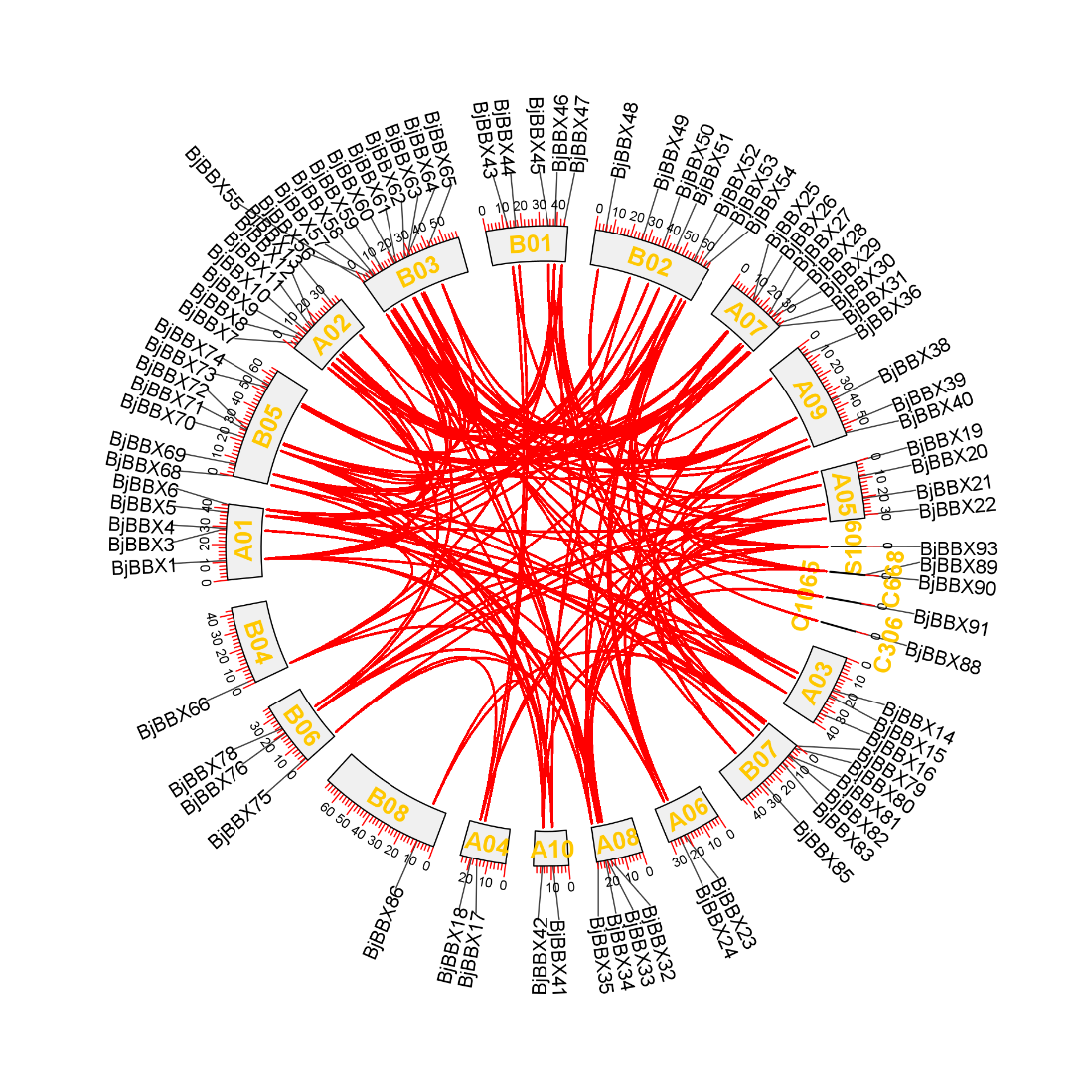


Fig. S5-9


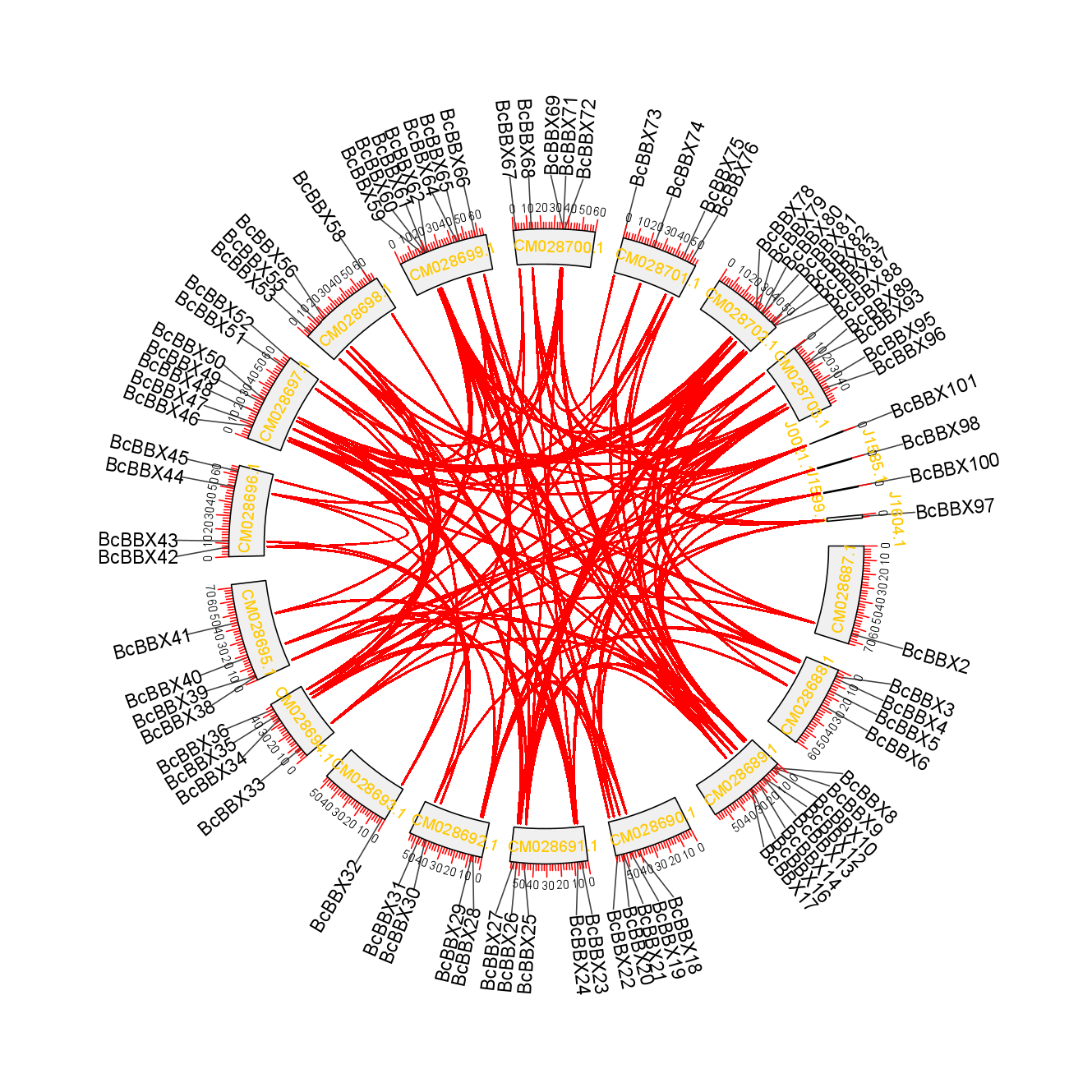

Supplement: Supplementary file 5 — Additional file 5: Figure S5. Synteny of BBX genes in each Brassicaceae genome. Figure S5–1 Synteny of AtBBX genes in A. thaliana genome. Figure S5–2 Synteny of BnaBBX genes in B. napus genome. Figure S5–3 Synteny of BoBBX genes in B. oleracea genome. Figure S5–4 Synteny of BrBBX genes in B. rapa genome. Figure S5–5 Synteny of CrBBX genes in C. rubella genome. Figure S5–6 Synteny of CsBBX genes in C. sativa genome. Figure S5–7 Synteny of BnBBX genes in B. nigra genome. Figure S5–8 Synteny of BjBBX genes in B.juncea genome. S109, C668, C1065 and C306 respectively indicates Super_scaffold_109_3169392_4351964_52914_567609, Contig668_1_483987, Contig1065 and Contig306 in the B.juncea genome. Figure S5–9 Synteny of BcBBX genes in B. carinata genome. J0021.1, J1585.1, J1599.1 and J1604.1 respectively indicates JAAMPC010000021.1, JAAMPC0100001585.1, JAAMPC0100001599.1 and JAAMPC0100001604.1 in the B. carinata genome. [file 12870_2021_3043_MOESM5_ESM.docx]

Fig. S6-1


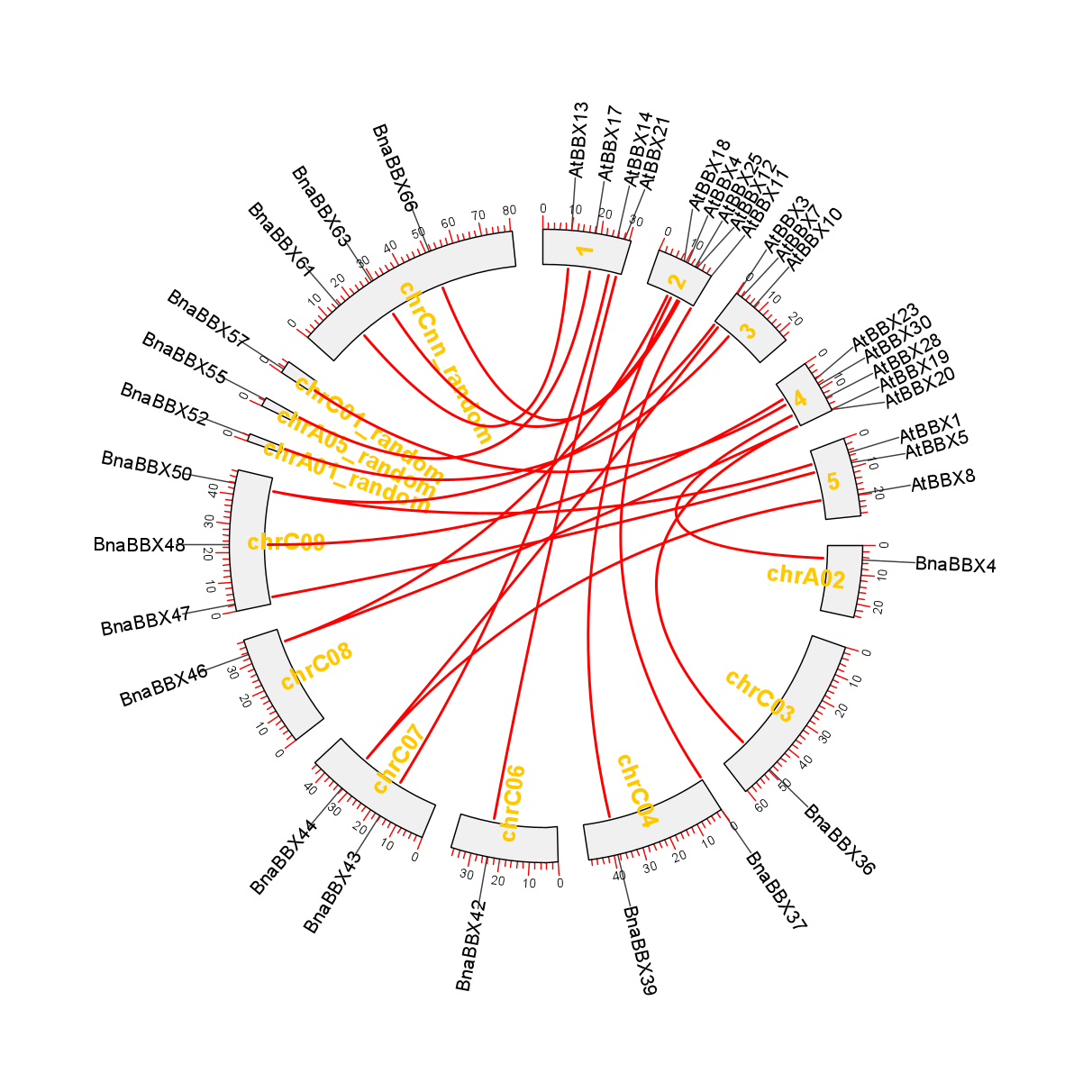


Fig. S6-2


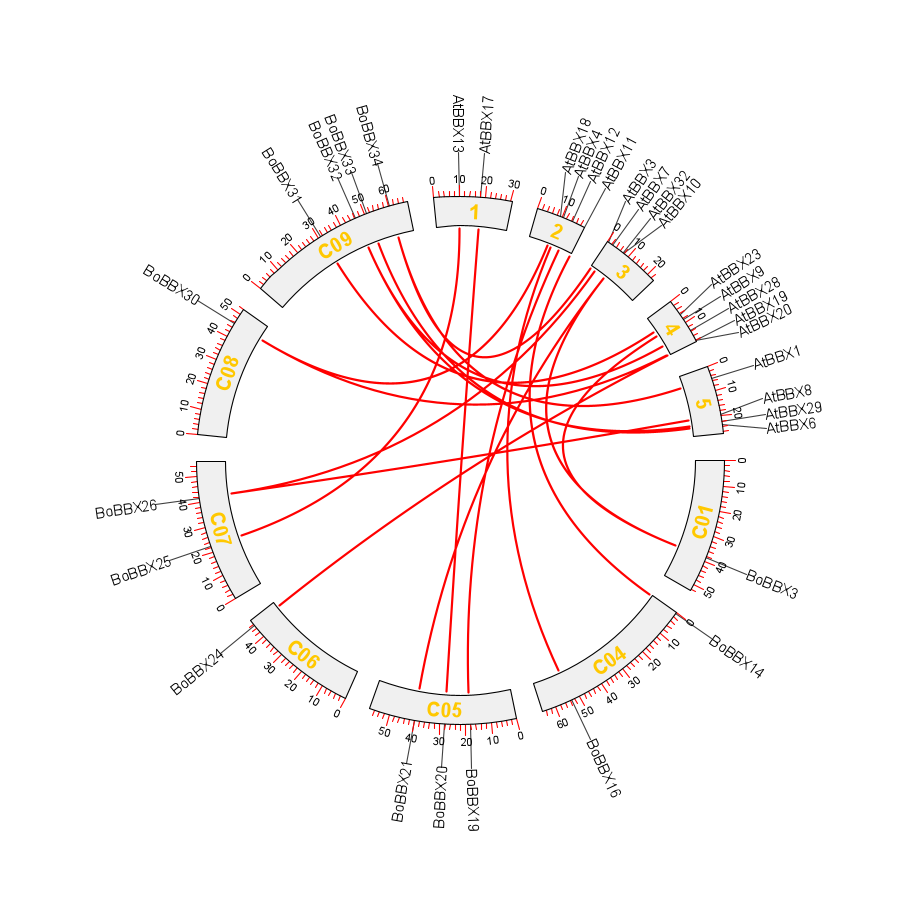


Fig. S6-3


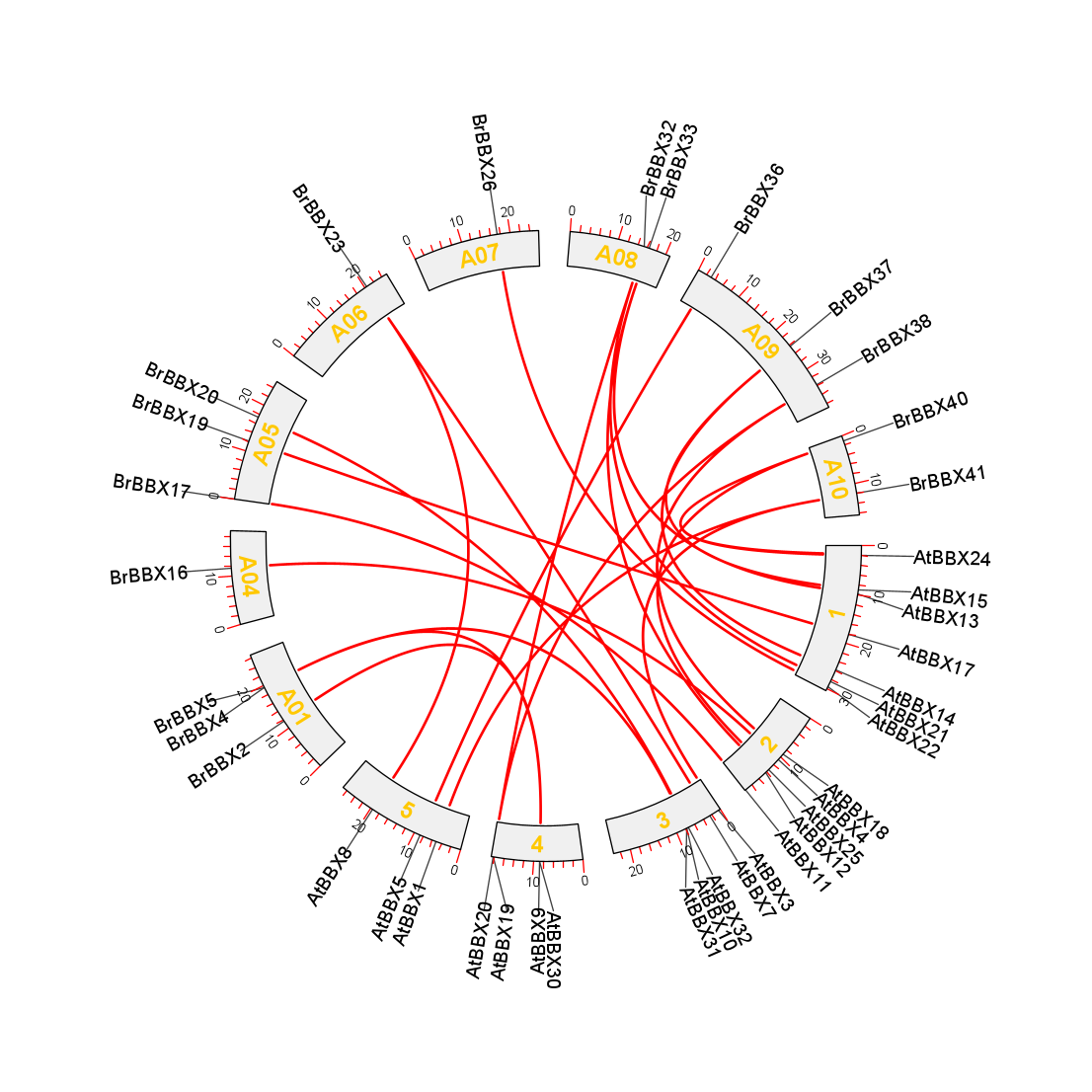


Fig. S6-4


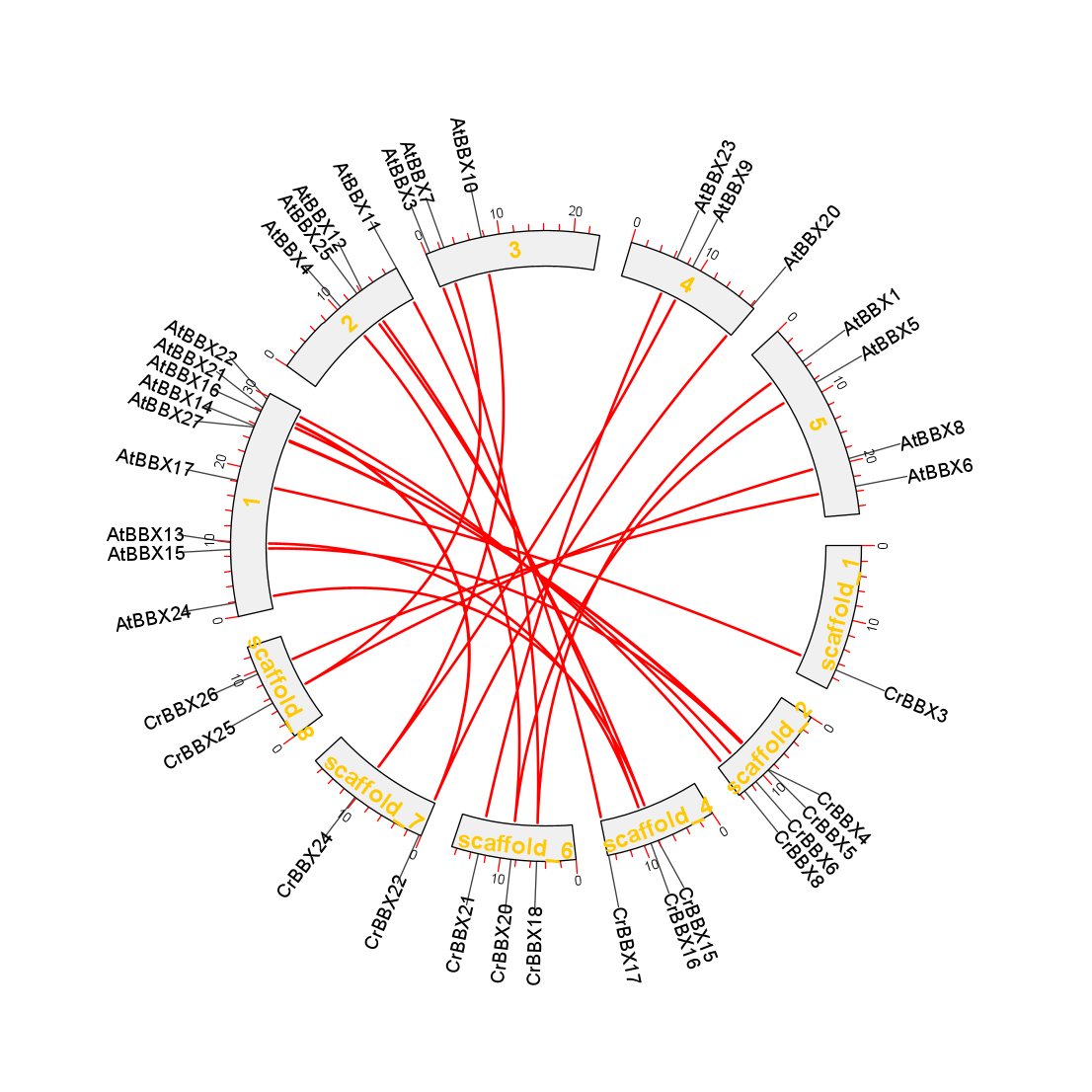


Fig. S6-5


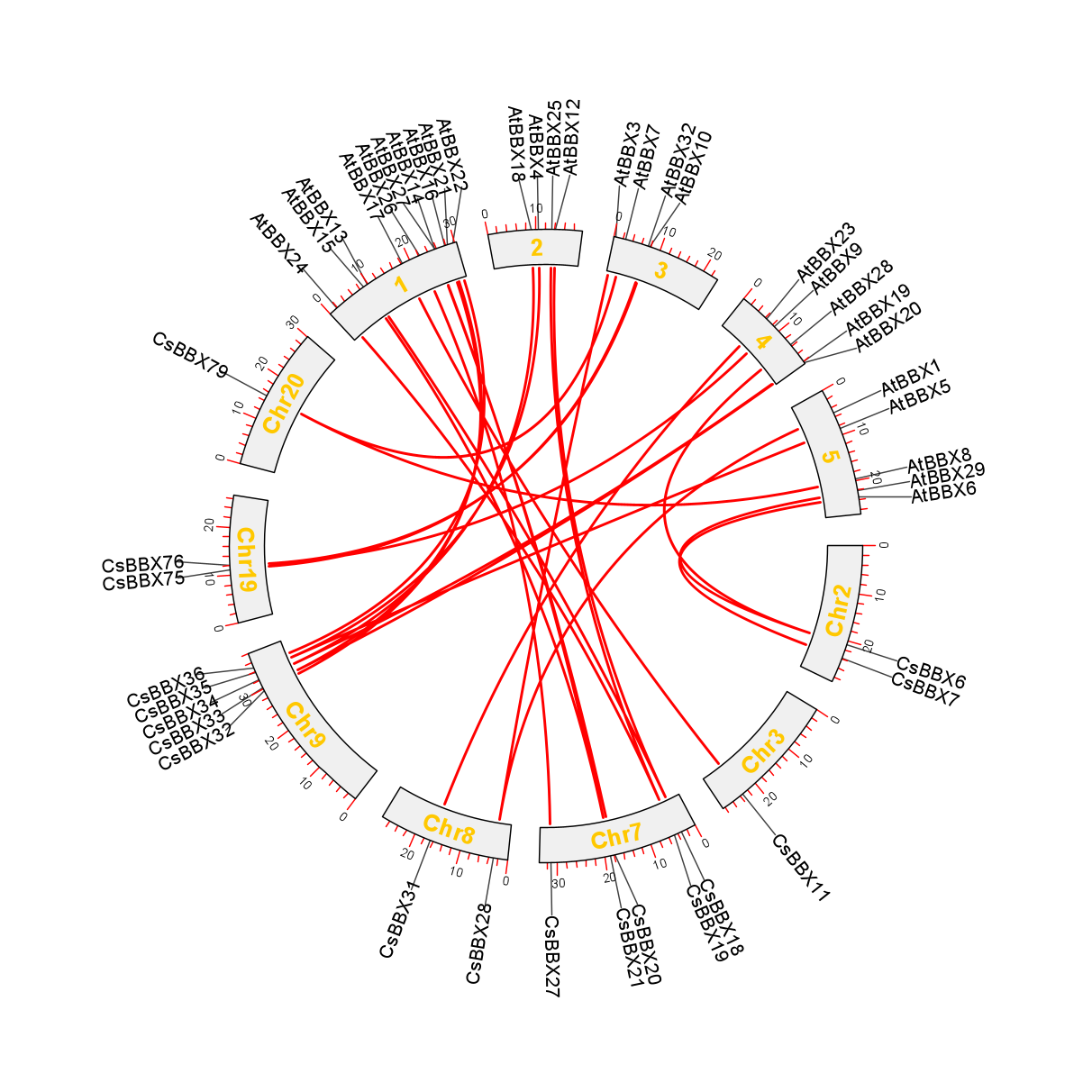


Fig. S6-6
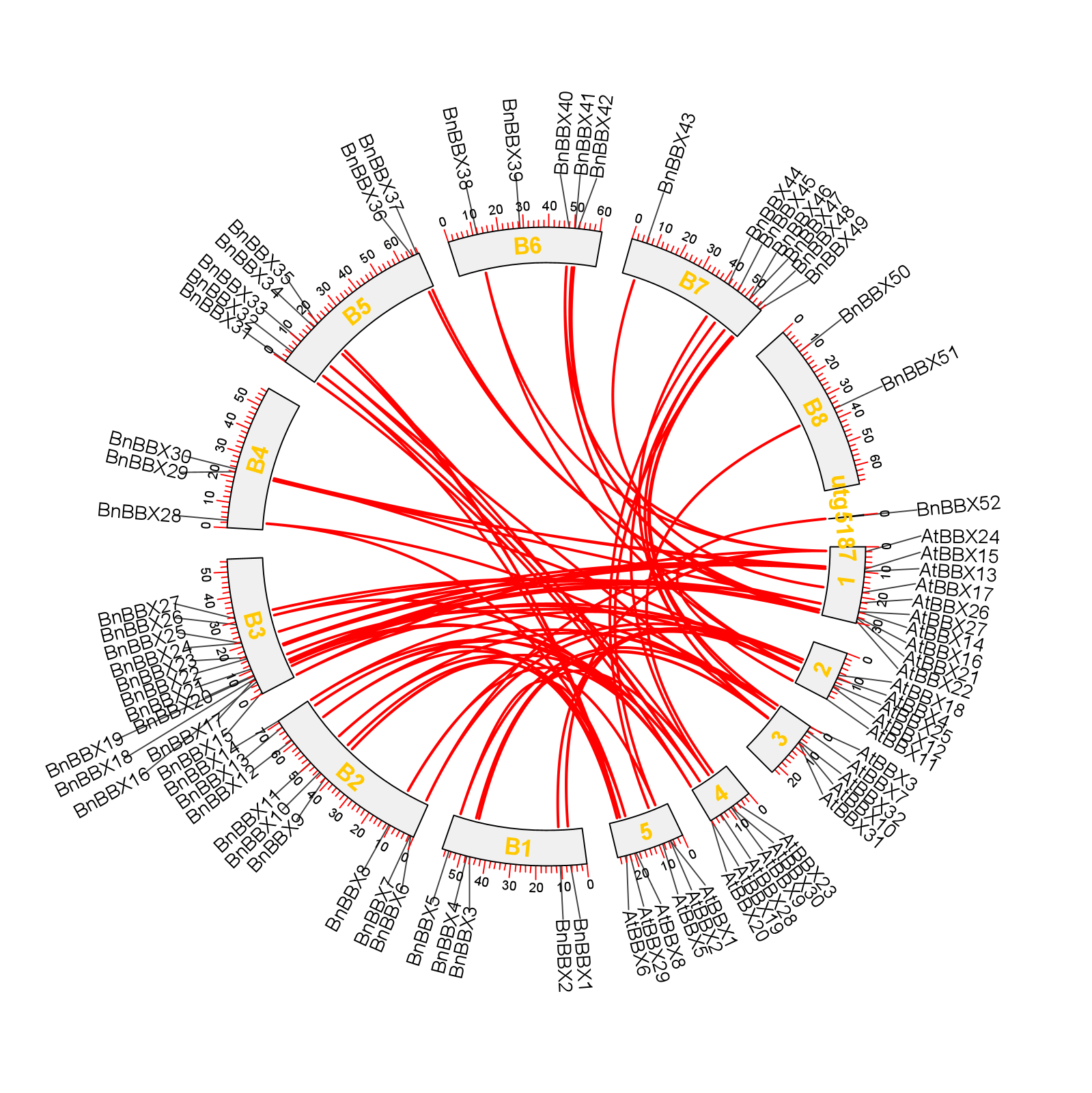


Fig. S6-7


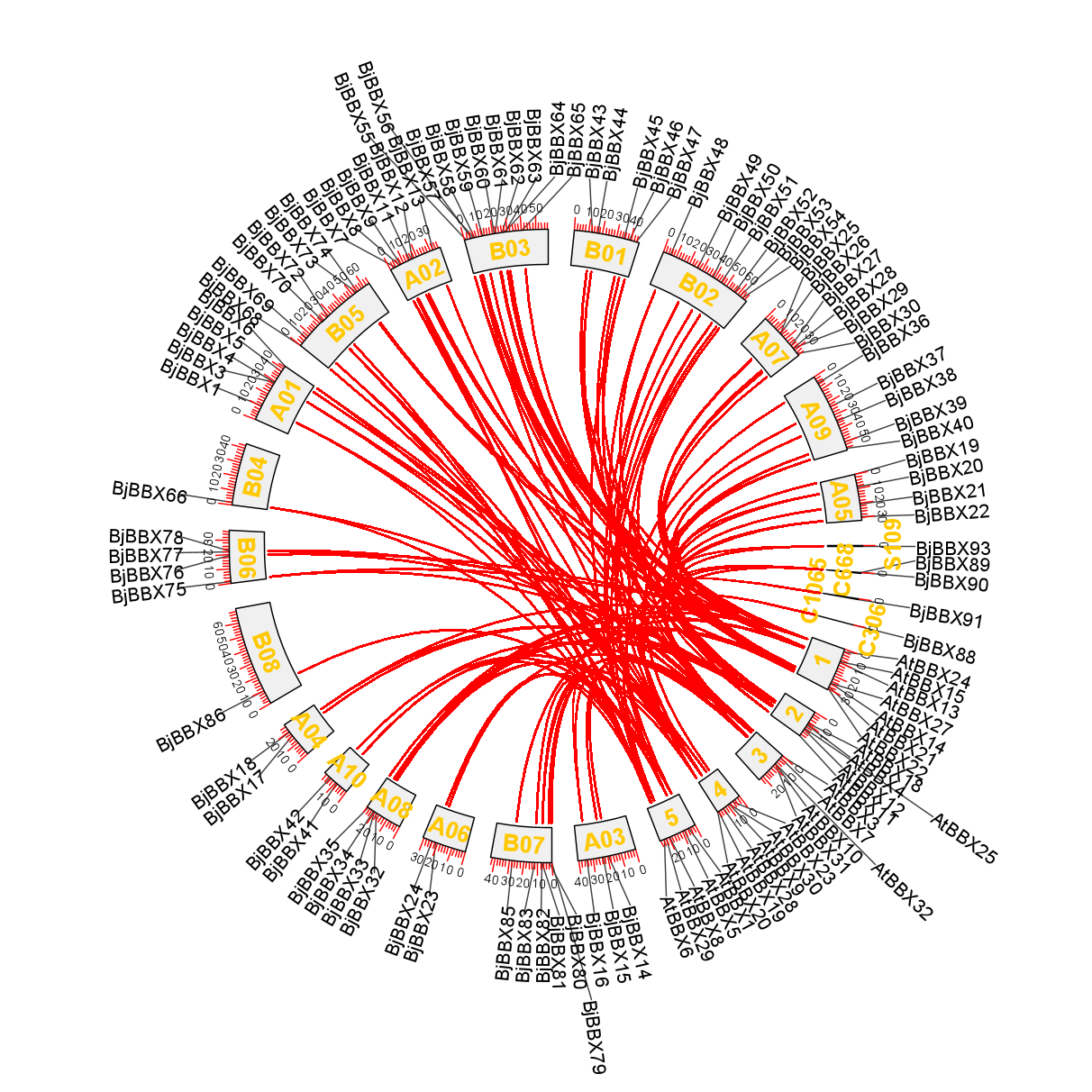


Fig. S6-8


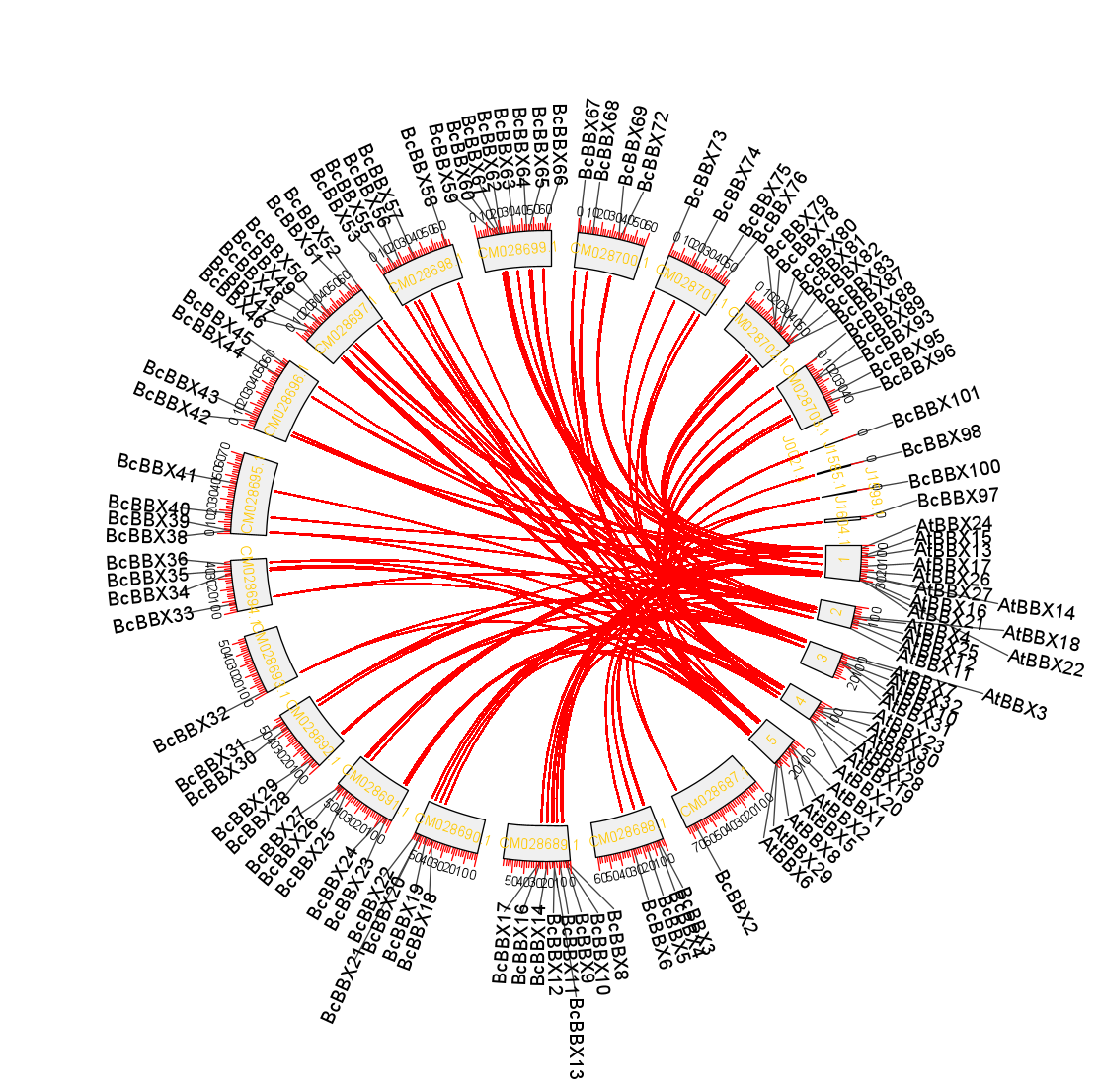

Supplement: Supplementary file 6 — Additional file 6: Figure S6. Synteny of BBX genes between A. thaliana and each other Brassicaceae. Figure S6–1 Synteny of BBX genes between A. thaliana and B. napus. Figure S6–2 Synteny of BBX genes between A. thaliana and B. oleracea. Figure S6–3 Synteny of BBX genes between A. thaliana and B. rapa. Figure S6–4 Synteny of BBX genes between A. thaliana and C. rubella. Figure S6–5 Synteny of BBX genes between A. thaliana and C. sativa. Figure S6–6 Synteny of BBX genes between A. thaliana and B. nigra. Figure S6–7 Synteny of BBX genes between A. thaliana and B.juncea. S109, C668, C1065 and C306 respectively indicates Super_scaffold_109_3169392_4351964_52914_567609, Contig668_1_483987, Contig1065 and Contig306 in the B.juncea genome. Figure S6–8 Synteny of BBX genes between A. thaliana and B. carinata. J0021.1, J1585.1, J1599.1 and J1604.1 respectively indicates JAAMPC010000021.1, J AAMPC010001585.1, J AAMPC010001599.1 and J AAMPC010001604.1 in B. carinata genome. [file 12870_2021_3043_MOESM6_ESM.docx]

Fig. S7-1


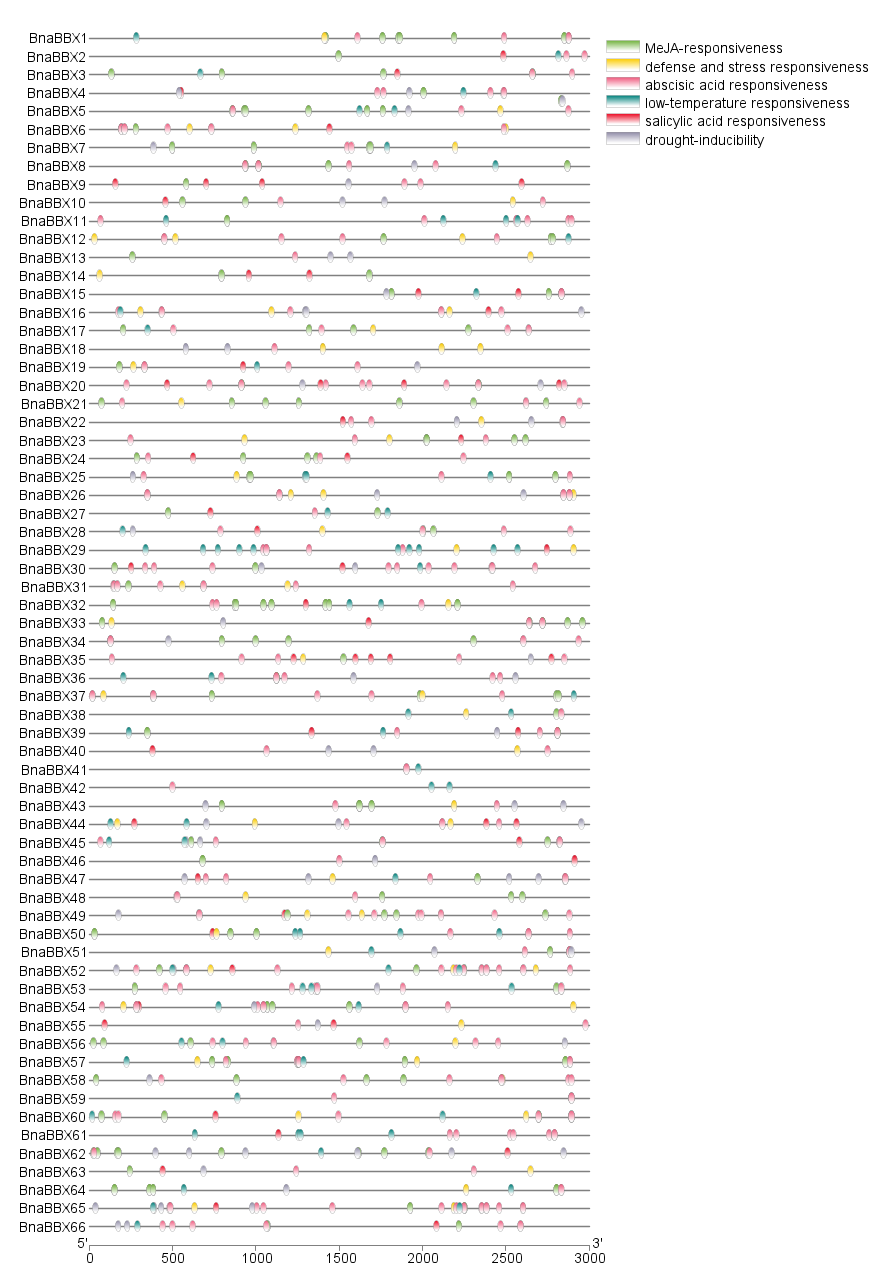


Fig. S7-2


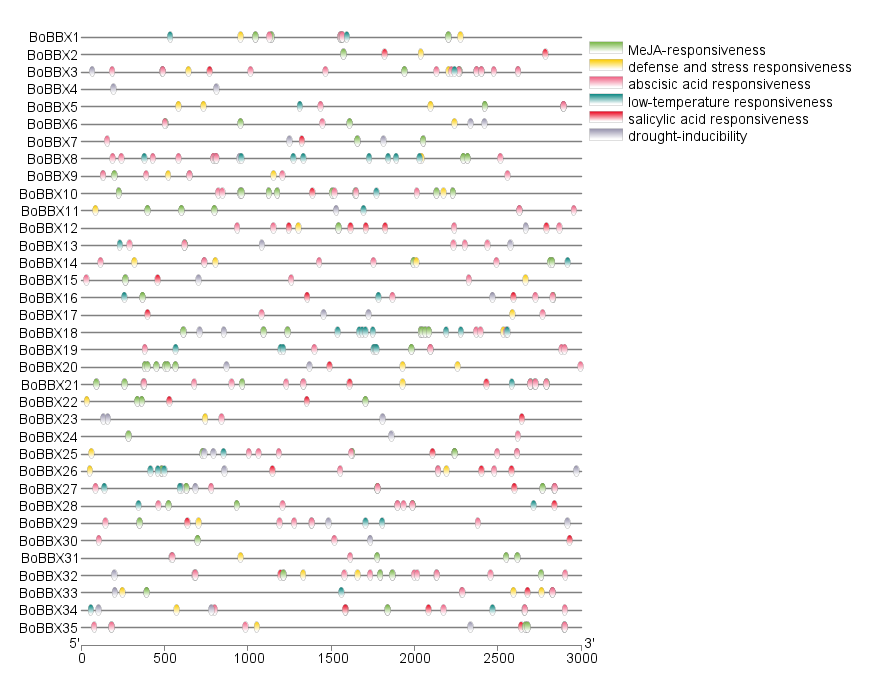


Fig. S7-3


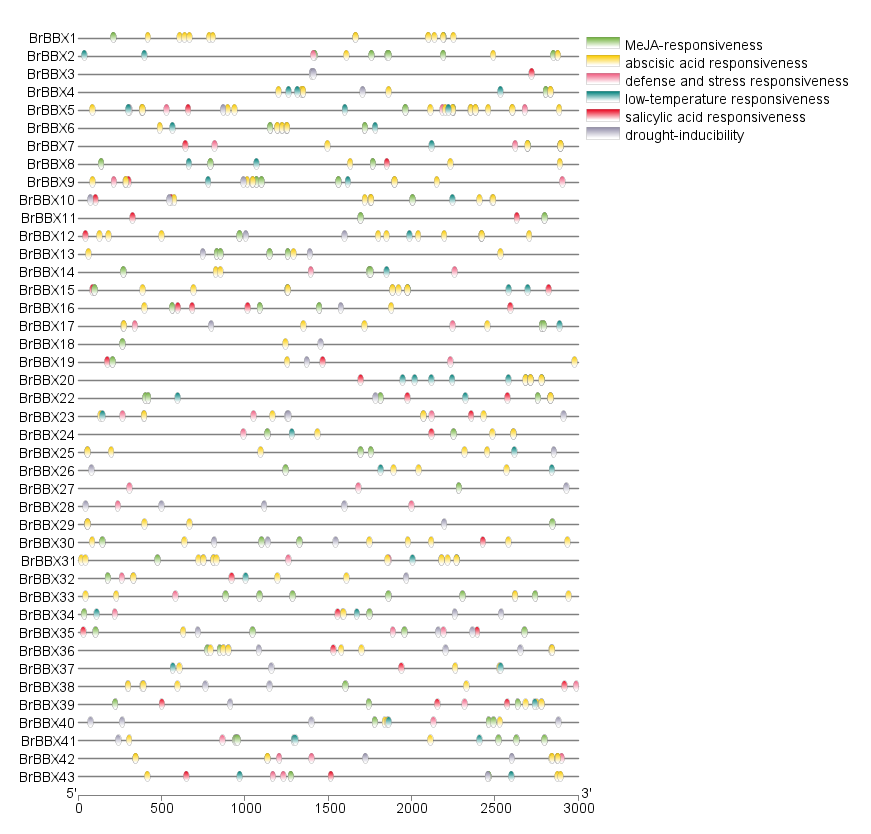


Fig. S7-4


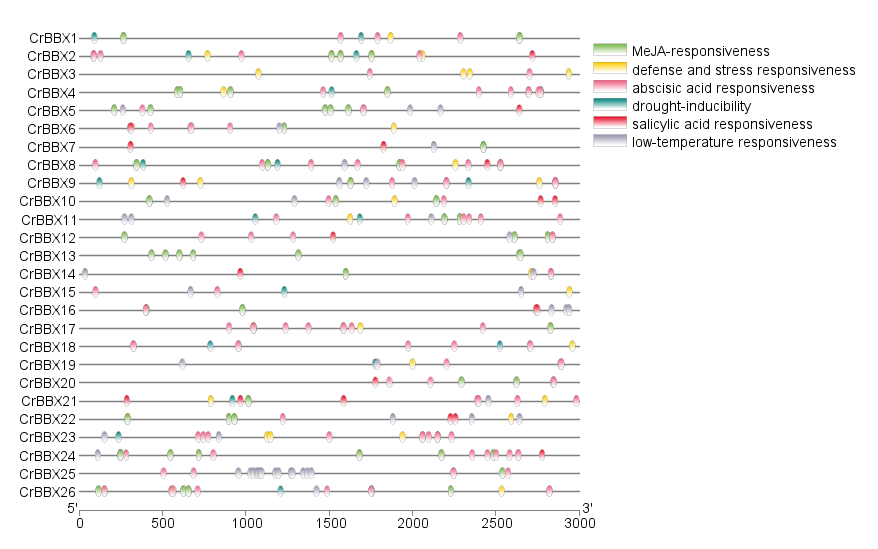


Fig. S7-5


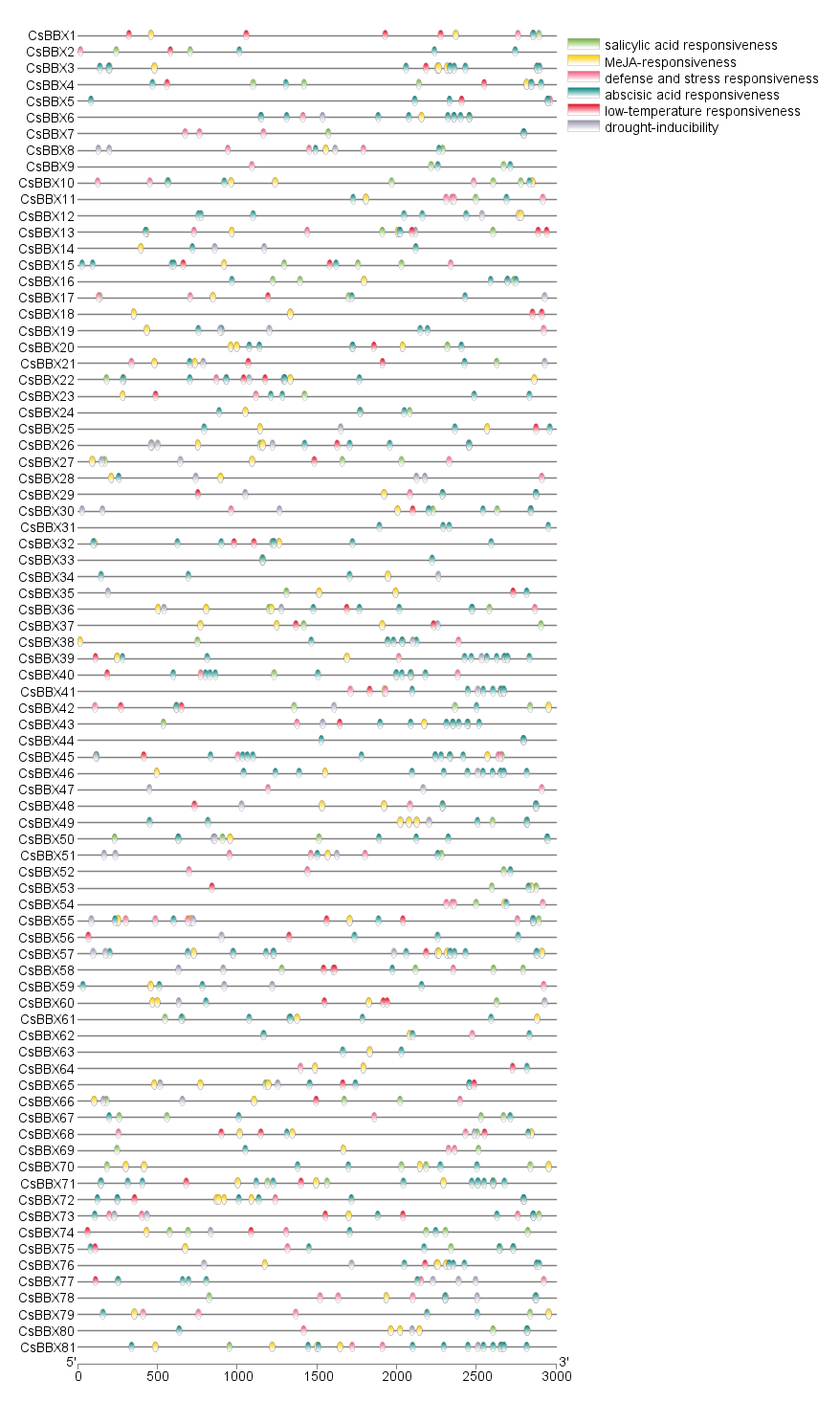


Fig. S7-6


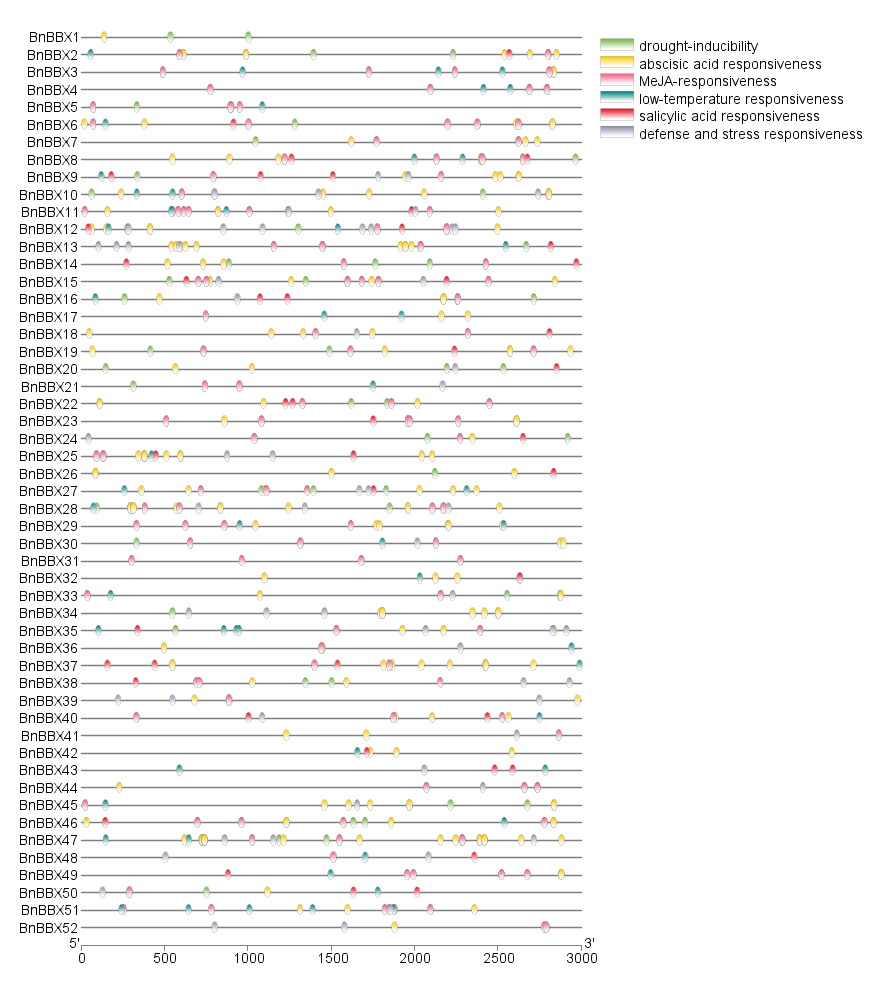


Fig. S7-7


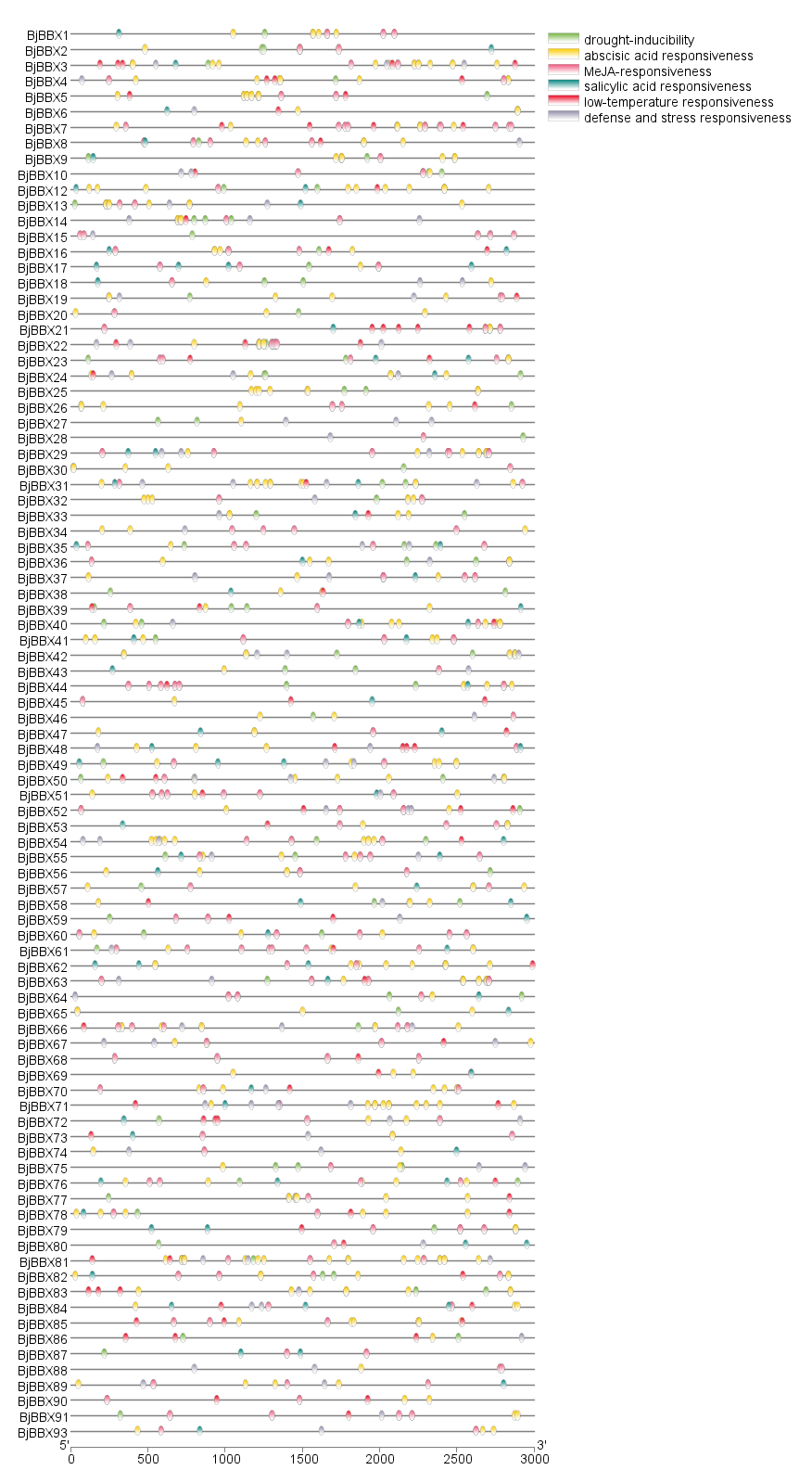


Fig. S7-8


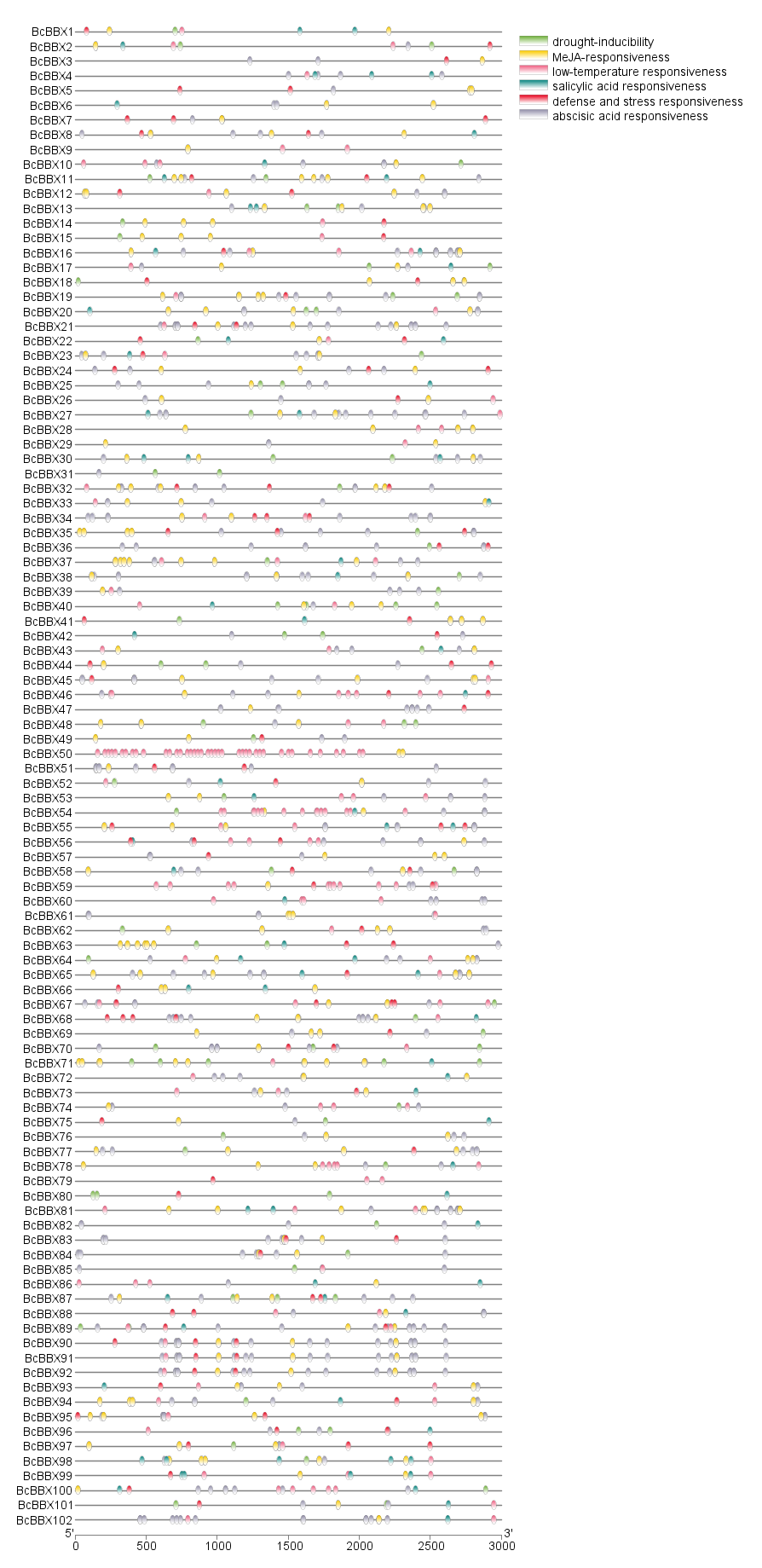

Supplement: Supplementary file 7 — Additional file 7: Figure S7. Promoter analysis of BBX genes. Figure S7–1 Cis-element identified in promoters of BnaBBXs. Figure S7–2 Cis-element identified in promoters of BoBBXs. Figure S7–3 Cis-element identified in promoters of BrBBXs. Figure S7–4 Cis-element identified in promoters of CrBBXs. Figure S7–5 Cis-element identified in promoters of CsBBXs. Figure S7–7 Cis-element identified in promoters of BjBBXs. Figure S7–8 Cis-element identified in promoters of BcBBXs. [file 12870_2021_3043_MOESM7_ESM.docx]
